# Kericho CLinic-Based ART Diagnostic Evaluation (CLADE): Design, Accrual, and Baseline Characteristics of a Randomized Controlled Trial Conducted in Predominately Rural, District-Level, HIV Clinics of Kenya

**DOI:** 10.1371/journal.pone.0116299

**Published:** 2015-02-23

**Authors:** Fredrick K. Sawe, Eunice Obiero, Peter Yegon, Rither C. Langat, Appolonia Aoko, Jemutai Tarus, Ignatius Kiptoo, Raphael K. Langat, Jonah Maswai, Margaret Bii, Samoel Khamadi, Kibet P. Shikuku, Nicole Close, Samuel Sinei, Douglas N. Shaffer

**Affiliations:** 1 Kenya Medical Research Institute/Walter Reed Project, Kericho, Kenya; 2 U.S. Military HIV Research Program, Walter Reed Army Institute of Research, Silver Spring, MD, United States of America; 3 Henry M. Jackson Foundation for the Advancement of Military Medicine, Rockville, MD, United States of America; 4 Kericho District Hospital, Kenya Ministry of Public Health and Sanitation, Kericho, Kenya; 5 HJF Medical Research International, Inc., Kericho, Kenya; 6 EmpiriStat, Mt Airy, MD, United States of America; University of New South Wales, AUSTRALIA

## Abstract

**Background:**

Prospective clinical trial data regarding routine HIV-1 viral load (VL) monitoring of antiretroviral therapy (ART) in non-research clinics of Sub-Saharan Africa are needed for policy makers.

**Methods:**

CLinic-based ART Diagnostic Evaluation (CLADE) is a randomized, controlled trial (RCT) evaluating feasibility, superiority, and cost-effectiveness of routine VL vs. standard of care (clinical and immunological) monitoring in adults initiating dual nucleoside reverse transcriptase inhibitor (NRTI)+non-NRTI ART. Participants were randomized (1:1) at 7 predominately rural, non-research, district-level clinics of western Kenya. Descriptive statistics present accrual patterns and baseline cohort characteristics.

**Results:**

Over 15 months, 820 adults enrolled at 7 sites with 86–152 enrolled per site. Monthly site enrollment ranged from 2–92 participants. Full (100%) informed consent compliance was independently documented. Half (49.9%) had HIV diagnosed through voluntary counseling and testing. Study arms were similar: mostly females (57.6%) aged 37.6 (SD = 9.0) years with low CD4 (166 [SD = 106]) cells/m^3^). Notable proportions had WHO Stage III or IV disease (28.7%), BMI <18.5 kg/m^2^ (23.1%), and a history of tuberculosis (5.6%) or were receiving tuberculosis treatment (8.2%) at ART initiation. In the routine VL arm, 407/409 (99.5%) received baseline VL (234,577 SD = 151,055 copies/ml). All participants received lamivudine; 49.8% started zidovudine followed by 38.4% stavudine and 11.8% tenofovir; and, 64.4% received nevirapine as nNRTI (35.6% efavirenz).

**Conclusions:**

A RCT can be enrolled successfully in rural, non-research, resource limited, district-level clinics in western Kenya. Many adults presenting for ART have advanced HIV/AIDS, emphasizing the importance of universal HIV testing and linkage-to-care campaigns.

**Trial Registration:**

ClinicalTrials.gov NCT01791556

## Introduction

Both CD4+ T-cell count (CD4 count) and plasma HIV-1 RNA (viral load [VL]) are standard of care assessments of immune function and HIV viremia in patients receiving antiretroviral therapy (ART) in resource-rich settings.[1, 2] In resource-limited settings such as sub-Saharan Africa where marked advancements have been made in ART roll-out, ART monitoring has been primarily based upon clinical (i.e. World Health Organization [WHO] Stage) and immunological (i.e. CD4 count) evaluations.[3] The poor predictive value of WHO clinical stage and/or CD4 count in identifying virologic failures, however, has been well documented.[4–8] 2010 WHO guidelines recommended VL where available every 6 months to evaluate viremia and the use of “confirmatory” VL monitoring for suspected treatment failures. [9] More recently, 2013 WHO guidelines recommend VL as the preferred monitoring approach to diagnose and confirm ART treatment failure (strong recommendation, low-quality evidence). [10] In Kenya, Ministry of Health (MoH) guidelines now recommend “targeted” VL monitoring in effort to assess viremia in cases of suspected clinical or immunological treatment failure.[11]

With marked advances in ART roll-out in sub-Saharan Africa planned, it is important to evaluate the role of routine VL monitoring.[12–14] Despite recent guidelines’ recommendations for VL monitoring, data regarding VL monitoring in the relevant clinical settings is limited.[9] Primary data from randomized, controlled trials (RCT) including cost-effectiveness will be critical for policy makers in an effort to assure optimal ART monitoring for best short and long-term outcomes in the face of inherent tensions VL monitoring brings with regard to constrained costs, limited infrastructure, and maximizing access. Here, we describe the design, accrual, and baseline characteristics of a RCT designed to evaluate the feasibility, superiority, and cost-effectiveness of routine VL monitoring in predominately rural, district-level ART clinics in Kenya.

## Methods

### Study Design

The CLinic-based ART Diagnostic Evaluation (CLADE) study is an open-label RCT evaluating the superiority and cost-effectiveness of two VL monitoring approaches in adults initiating first-line ART: 1. routine care (Arm A) consisting of clinical (WHO staging) and immunological (CD4 count) monitoring every 6 months with confirmatory or targeted VL monitoring based upon Kenya MoH and WHO guidelines standard of care; and 2. VL guided care (Arm B) consisting of VL, CD4 count, and clinical evaluations conducted every 6 months.[15]

CLADE has two primary objectives: 1. compare proportions of viral failures defined as an HIV-1 RNA VL greater than 1,000 copies/ml after 18 months of follow-up; and 2. evaluate the cost-effectiveness of routine VL monitoring in addition to CD4 and clinical monitoring by measuring actual health outcome costs. The second primary objective, cost-effectiveness, analysis will be based upon actual costs incurred. Analyses will proceed after all follow-up data are available and based upon established methods.[16–18]. The study includes 12 secondary objectives broadly covering clinical (e.g. disease progression, hospitalization, mortality) and laboratory (e.g. primary and secondary HIV drug resistance and HIV sub-type) secondary endpoints; evaluating differing ART regimens and viral failure criteria; and capacity development with feasibility evaluation of conducting routine VL evaluated by monitoring under a RCT with Good Clinical Practices.

The CLADE sample size is 820 HIV infected, ART naïve adults initiating first line ART. Overall, a total of 734 subjects (367 per arm) were required in order to achieve 80% power (alpha = 5%) with a two-sided z-test with continuity correction to test the null hypothesis. Initial sample size parameter estimates were based upon local viral failure rates from a Phase III therapeutics study (<1%) utilizing routine VL monitoring and early field data from ART roll-out (approximately 12%). For the CLADE study designed to evaluate viral failure rates in a non-research setting, a more conservative delta was utilized with probabilities of viral failure in routine care (Arm A) and viral load (Arm B) arms being 8% and 3%, respectively.

Randomization (1:1) in permuted blocks to arm A or arm B occurred at day of ART initiation at each site using telephone procedures between the site Study Coordinator and data management staff at the Kenya Medical Research Institute/Walter Reed Project (KEMRI/WRP) Clinical Research Center. Randomization was independently verified through data extraction processes and data quality assurance/quality control procedures.

This sample size allowed for one interim analysis conducted when half of the participants completed the final, 18-month visit and a final analysis at the end of the study using an O’Brien-Fleming spending function. The lower and upper boundaries for the interim analysis are z (lower) = -2.96 and z (upper) = 2.96, with a p-value = 0.0031. Two additional inflations were made to the sample size: 5% for lost to follow-up and 5% in anticipation of participants on the routine care arm A receiving confirmatory VL monitoring. This 10% inflation resulted in an overall study sample size of 818.

Inclusion criteria were: HIV-1 infected adults >18 years old who provided written informed consent and started first line ART based upon Kenya MoH guidelines. Exclusion criteria were: pregnancy at time of ART initiation; any reason (medical, social, or other) the clinic felt would prevent the participant from routine ART follow-up; or, any reason that makes risks of study participation outweigh benefits (e.g. HIV partner non-disclosure and concern over participation in research).

### Study Setting

**Fig 1:**
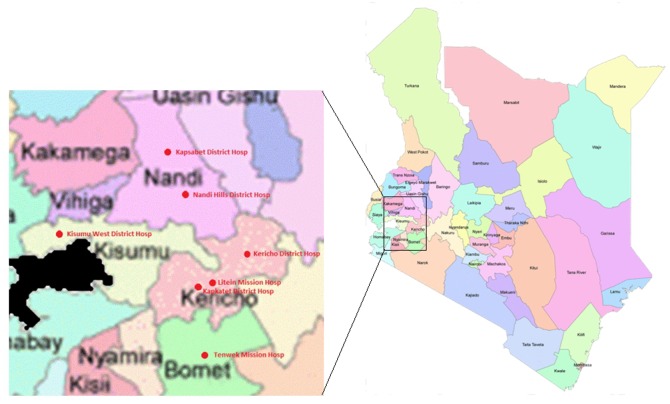
Participating Sites in the Clinic-based ART Diagnostic Evaluation (CLADE) Trial. Map data courtesy of Kenya Open Data (https://opendata.go.ke).

CLADE was conducted at 7 district level ART clinics (5 MoH and 2 Faith-based organization [FBO]) in western Kenya’s Rift Valley (n = 6) and Nyanza (n = 1) Provinces (Fig. 1). Over half (51.4%) of all HIV-infected adults in Kenya live in the Rift Valley and Nyanza Provinces based upon the 2007 Kenya AIDS Indicator Survey.[19] Urban and rural HIV prevalence rates among adults from this national survey were 6.3% and 6.4% for Rift Valley Province and 14.9% and 13.9% for Nyanza Province, respectively.[19] Variability in prevalence exists based upon populations studied and methods utilized. HIV-1 prevalence rates were 14.3% from a community-based cohort study and 31.4% in a high risk cohort study based in the Kericho District of the Rift Valley Province.[20,21] Corresponding 12 month HIV incidence rates were 1.4 and 4.0 per 100 person years, respectively.[21,22]

With approximately 70% of Kenya’s population being rural, a majority of HIV disease burden is in rural settings.[19, 23] All clinics participating in CLADE are in 4 counties of Kenya: Kericho, Nandi, Bomet and Kisumu. While Kericho town has become more densely populated, the county itself and catchment area are 61.3% rural. Nandi county is 86.4% rural, and Bomet county is 85.2% rural. Kisumu county (including Kisumu, Kenya’s third largest city) is urban with a rural population of 47.6%, although Kisumu West District Hospital as a participating clinic falls within the Kisumu rural constituency.[23]

Serving as a regional referral hospital, Kericho District Hospital has an out-patient HIV clinic size of approximately 14,000 patients. Other clinic sizes range from 3,000 to 11,000 HIV infected patients.[24,25] All of the HIV out-patient clinics are staffed primarily by clinical officers and nurses, the primary, health care work force in Kenya. Six of the clinics have medical officers and three have Internal Medicine MMED physicians available for consultation. Each participating site had an existing HIV clinic for 3–5 years prior to study opening.[24, 25]

### Sponsor, Regulatory Oversight, and Community Advisory Board

CLADE is sponsored by the U.S. Office of the Global AIDS Coordinator (#KE-07–0044), and is a registered clinical trial (registration number NCT01791556).[15, 26] The trial was conducted by the Kenya Medical Research Institute/Walter Reed Project (KEMRI/WRP) HIV program in collaboration with the Kenya MoH (now Kenya Ministry of Medical Services) under the auspices of the Kenya Medical Research Institute (KEMRI) and US Military HIV Research Program (MHRP).[24, 25, 27] Monitoring for GCP compliance was conducted by the MHRP Regulatory Office Center focusing upon regulatory documents, clinic study data, and 100% informed consent review.

A seven-member, independent Data Monitoring Committee (DMC) consisting of Kenyan (n = 4) and international (n = 3) experts including the KEMRI/WRP Community Advisory Board (CAB) chairman met prior to protocol submission and every six months (+/- 30 days) after the first participant was randomized. The DMC terms of reference included: 1. examine over time the endpoint data and safety signals, including second line therapy switches; and 2. independently review the general progress and conduct of the CLADE study.

A 13 member, independent CAB oversees and provides guidance on all research and care and treatment activities of the KEMRI/WRP program.

### Ethics Statement

CLADE had full review and approval by 3 institutional review boards and/or ethics review committees: the Kenya Medical Research Institute, Tenwek Mission Hospital, and Walter Reed Army Institute of Research.[28–30] All participants provided written informed consent either in English, Swahili or Dholuo. The latest protocol document approved was version 2.5.

### Study Care

Care for CLADE participants was in accordance with the standard of care as outlined by the Kenya MoH. The MoH in collaboration with KEMRI/WRP under the United States President’s Emergency Plan For AIDS Relief (PEPFAR) program supports HIV care, prevention, and treatment services in the southern Rift Valley Province of Kenya (SRV) with a population of approximately 2.5 million.[24, 25, 27, 31] Beginning with 4 initial HIV treatment sites in 2004, the KEMRI/WRP program coverage now includes 11 district level, primary ART facilities (including 6 of the 7 CLADE sites), 91 lower level facilities (i.e. sub-district hospitals, rural health centers, dispensaries), and over 400 PMTCT sites. The KEMRI/WRP PEPFAR program includes clinical and laboratory program managers and staff as well as an administrative core who support partners in the SRV providing HIV services through PEPFAR funding.

**A. HIV Diagnosis and Pre-ART Evaluations.** HIV diagnosis was established by the use of licensed rapid diagnostic tests (RDT) in accordance with HIV Testing and Counseling (HTC) guidelines.[32–34] Confirmation of HIV diagnosis was a criteria for study eligibility. Evaluation and preparation for ART included components of an “essential care package” for Persons Living With HIV/AIDS (PLWHA): counseling (HIV and general ART) and psychosocial support; nutritional assessment and supplementation; medical evaluation and WHO staging; laboratory evaluation; and, treatment and prophylaxis of opportunistic infections (OIs).[11, 35, 36]

Universal trimethoprim/sulfamethoxazole prophylaxis is used in the region. Screening and prophylaxis for *Cryptococcal* meningitis is conducted in persons with CD4 counts less than 100 cells/mm^3^. Routine screening for Tuberculosis (Tb) is conducted at all sites offering ART, with those suspected of Tb undergoing further diagnostic evaluations based on sputum microscopy and X-ray. Isoniazid preventive therapy (IPT) was being introduced but not routinely conducted at any of the CLADE sites during the study. During pre-ART evaluations, patients were introduced to the CLADE study at participating sites and provided copies of the informed consent. Follow-up discussions with the health care provider and/or site study coordinator were made available.

**B. First Line ART and Treatment of Opportunistic Infections.** ART and treatments for OIs and non-HIV related illness were based upon national guidelines.[11, 35–38] Initially, ART criteria included all patients with WHO Stage III or IV disease as well as those with WHO Stage I or II and a CD4 count less < 250 cells/mm^3^.[35] With newer guidelines, ART was recommended for all patients with a CD4 count < 350 cells/mm^3^ or in those > 350 cells/mm^3^ with Mycobacterium tuberculosis (Tb), HIV/Hepatitis B virus (HBV) co-infection, or HIV nephropathy.[11] First line ART consisted of two nucleoside reverse transcriptase inhibitors (NRTIs) and one non-nucleoside reverse transcriptase inhibitors (nNRTI): stavudine or zidovudine, lamivudine, and nevirapine or efavirenz. This changed when tenofovir became available and stavudine was removed from first line recommendations.[10, 11]

**C. Laboratory Evaluations.** All participating sites conducted MoH recommended ART safety evaluations (alanine aminotransferase/aspartate aminotransferase, creatinine, hemoglobin), and several were capable of more extensive chemistries and hematology tests, and urine pregnancy testing. In addition, sites conducted evaluations for co-infections including but not limited to microscopy for sputum acid fast bacilli and malaria blood parasites, cryptococcal antigen testing and Indian Ink, and hepatitis B serology. Five laboratories performed CD4 count evaluations with two sites relying upon local partner sites (Nandi Hills and Litein relying upon Kapsabet and Kapkatet, respectively). All laboratories were enrolled in External Quality Assurance (EQA programs through Human Quality Assessment Services (HuQAS) conducted quarterly.[39]

The KEMRI/WRP CRC laboratory conducts HIV-1 RNA (VL) testing for all clinics in the SRV program and serves as a back-up laboratory when needed. The Roche Amplicor v1.5 was used for VL testing. The Abbott m2000 system and Roche Taqman were used for back-up. Plasma separator preparation tubes were used for storage and transport of VL specimens to the central laboratory within 24 hours for all sites except for Kericho District Hospital where samples were delivered immediately. The KEMRI/WRP laboratory is enrolled in numerous EQA programs and maintains College of American Pathologist (CAP) accreditation (2008, 2010, and 2012).[40]

### Capacity Development

Key secondary objectives of CLADE focused upon the development of local capacity to incorporate VL monitoring consistent with newer guidelines and improve treatment failure competency. “Treatment failure retreats” were held at least bi-annually with all regional HIV programs. Expert lectures were incorporated. Cognizant of the need for research conducted outside of the traditional research setting and in response to requests for capacity development by the MoH partners, clinical research capacity was developed at each site. General research conduct and GCP were taught with each site having a dedicated Study Coordinator completing human research subjects training.[41] Finally, the Data Manager (PY) with a BS in Computer Science was sponsored by the study for enrollment in the University of Nairobi Medical Statistics Masters degree program with project work tied to secondary objective analysis (early morbidity and mortality).

### Study Data and Analyses

Study participants had a study folder kept at the ART clinic in a locked filing cabinet with access limited to study staff. The CLADE study folder consisted of case report forms (CRFs) mirroring the participant’s clinical record with data extracted as necessary for study analyses. Based upon concerns shared by MoH and FBO partners regarding the time and burden related to collection of study data for this first of its kind clinical trial, two tiers of CRFs were utilized: primary CRFs that included data linked to human subjects protection/GCP and key study objectives including the primary endpoint, for which 100% data capture was expected; and, secondary CRFs that included data captured by the clinic but not essential to the study objectives or primary endpoint (e.g. other clinical laboratory data, demographics), for which 100% was the goal but not absolute. The CLADE study folder had no personal identifying information. The folder was identified by the patient clinic “HAART” number (assigned at all clinics sequentially as patients entered clinics) and 4-digit random Subject Identification Number. All study procedures, including data collection, were conducted under study specific Standard Operating Procedures (SOPs).

Procedurally, extraction of relevant clinic data and population of the study database occurred in 3 steps. First, relevant data were recorded on visit Case Report Forms (CRFs) at the time of routine HIV clinic evaluations. Second, every 2 weeks or at a frequency necessary depending upon site accrual, members of the IT data team reported to study sites to enter CRF data onto password protected study laptops with 128-bit encryption. Finally, data was downloaded to the database at the KEMRI/WRP CRC IT Department daily or at a frequency no greater than once weekly. After central data download, laptop data was erased. No identifying information was kept on the CLADE laptops.

The study database, created and maintained by KEMRI/WRP IT staff, is a relational database management system (RDBMS) with integrated data management and analysis software. The application was a single entry system with programmed logic checks and prompts. The CLADE database is stored on a password protected, secured folder on the CRC research servers. All CLADE data is managed and analyzed in Kenya at the KEMRI/WRP CRC IT Department. For accrual and baseline enrollment data presented, routine, descriptive summary statistics are used. Analyses for data presented here are conducted in Kericho on the database locked January 31, 2013 with statistical validation by EmpiriStat.

## Results

### Enrollment

**Fig 2:**
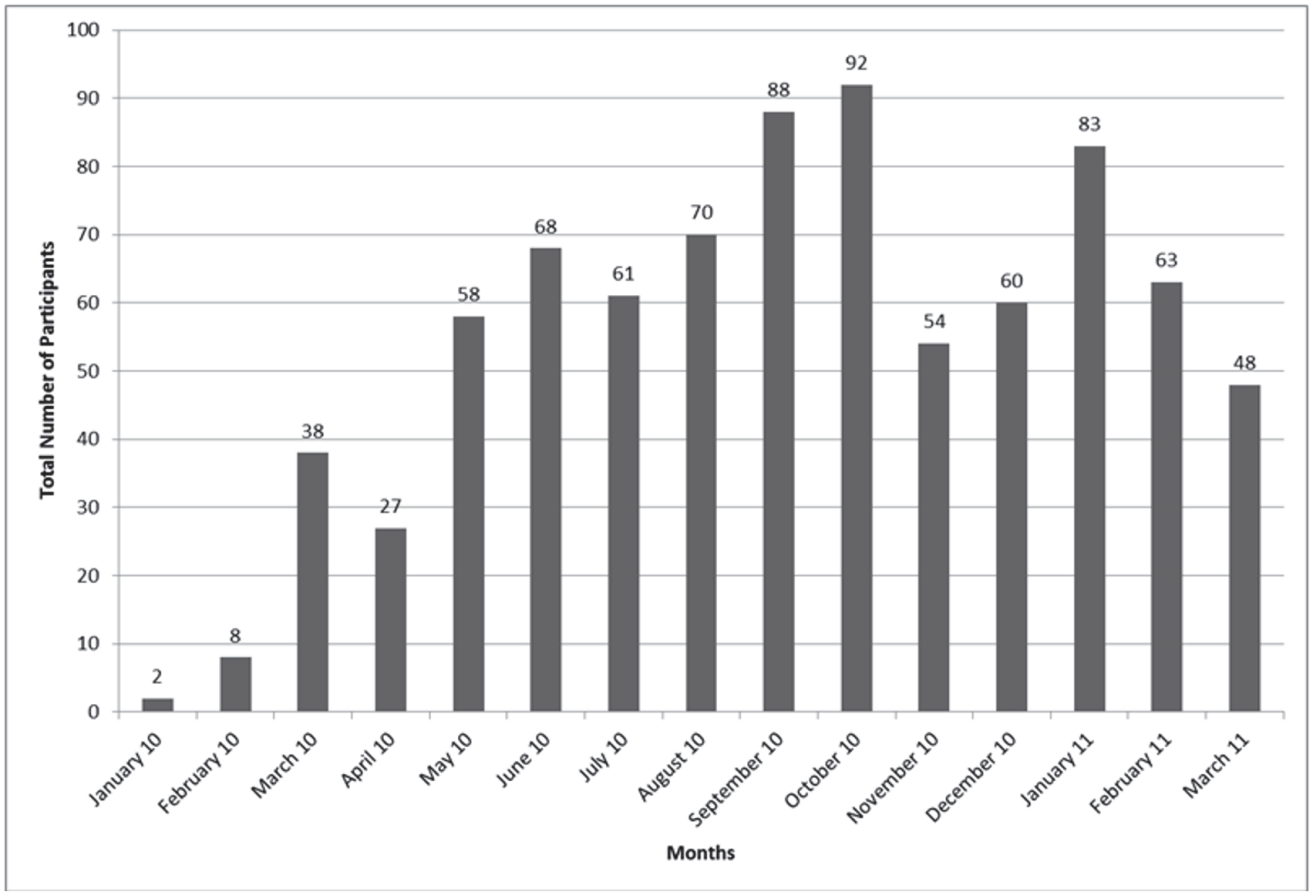
Participant Enrollment by Month in the CLinic-based ART Diagnostic Evaluation (CLADE) Trial (n = 820).

Beginning in January 2010, 820 adults initiating ART were enrolled at 7 sites with 86–152 enrolled per site (Table 1). Time for accrual at each site ranged from 6.6–11.2 months. Three site allocation adjustments were made throughout the enrollment period with sites finally enrolling a range of 71%-133% of their initial allocations. Full enrollment was accomplished in 15 months with monthly accrual ranging from 2 (first site activated) to 92 (all sites activated) participants (Fig. 2).

**Table 1:** Participant Recruitment and Accrual in the Clinic-based ART Diagnostic Evaluation Trial (CLADE).

Site	Start Date	Months of Accrual	Initial Allocation	Final Allocation	Average Enrollment/Month	Percentage of Initial Allocation
**AIC Litein Mission Hospital**	February 26, 2010	13	90	86	6.6	96%
**Kapkatet District Hospital**	January 26, 2010	14	90	120	8.5	133%
**Kapsabet District Hospital**	February 16, 2010	13	110	124	9.5	113%
**Kericho District Hospital**	January 28, 2010	14	150	152	10.8	101%
**Kisumu West District Hospital**	March 8, 2010	12	190	134	11.2	71%
**Nandi Hills District Hospital**	March 3, 2010	12	90	110	9.2	122%
**Tenwek Mission Hospital**	May 21, 2010	10	100	94	9.4	94%

### Baseline Characteristics

The 2 study arms were similar at baseline (Table 2). Overall, the baseline cohort represented mostly females (57.6%), aged 37.6 (SD = 9.0) years with advanced disease evidenced by low CD4 count (166 SD = 106.5 cells/m^3^) and high HIV-1 RNA (Arm B: 234,577 SD = 151,055copies/ml). Notable proportions had WHO Stage III or IV disease (28.7%), BMI <18.5 kg/m^2^ (23.1%), and ongoing Tb treatment at time of ART initiation (8.2%). While not reaching statistic significance, there was a trend in more participants in the routine care arm (7.1%) reporting a history of Tb compared to the VL arm (4.2%) (p = 0.07). Half (49.9%) received HIV testing through established client-initiated, voluntary counseling and testing (VCT) sites. Roughly half had HIV diagnosis established through provider initiated testing and counseling (PITC). The majority (91.5%) received primary (grades 1–8) or secondary (grades 9–12) education. Over half were married and in monogamous marriages (54.0%) with a small but notable proportion as known, discordant couples (7.2%). Half (54.3%) were unaware of their partner’s HIV status. Nearly all had normal or Grade I hemoglobin, liver function (AST/ALT) and renal function (creatinine).

There were no differences in ART initiated between the two arms. With all beginning ART receiving lamivudine as part of the dual NRTI backbone, about half (49.8%) started zidovudine followed by a slightly smaller proportion (38.4%) beginning stavudine as tenofovir was introduced as first line (11.8%). The majority (64.4%) received nevirapine as the nNRTI. Nearly all (97.3%) received trimethoprim/sulfamethoxazole prophylaxis. Few (8.3%) were receiving concurrent treatment for Tb.

**Table 2:** Baseline Characteristics of the CLinic-based ART Diagnostic Evaluation (CLADE) Trial Participants.

	Overall(n = 818)	Routine Care(Arm A)n = 409	Viral Load Guided Care (Arm B)n = 409	Missing Data
**Gender**				0 (0%)
**Male**	347 (42.4%)	172 (42.1%)	175 (42.8%)	
**Female**	471 (57.6%)	237 (57.9%)	234 (57.2%)	
**Age (years)**	37.6 (9.0)	37.5 (8.9)	37.8 (9.1)	0 (0%)
**CD4 count (cells/mm^3^)**	166 (106.5)	164 (109.3)	168 (103.6)	0 (0%)
**< = 50**	143 (17.5%)	76 (18.6%)	67 (16.4%)	
**51–199**	362 (44.3%)	173 (42.3%)	189 (46.2%)	
**200–349**	293 (35.8%)	148 (36.2%)	145 (35.4%)	
**> = 350**	20 (2.4%)	12 (2.9%)	8 (2.0%)	
**HIV-1 RNA (copies/ml)**	-	NA	234,577 (151,055)	-
**WHO Stage**				8 (0.97%)
**I**	302 (37.3%)	145 (35.9%)	157 (38.7%)	
**II**	275 (33.9%)	135 (33.4%)	140 (34.5%)	
**III**	202 (24.9%)	108 (26.7%)	94 (23.1%)	
**IV**	31 (3.8%)	16 (4.0%)	15 (3.7%)	
**Body Mass Index (kg/m** ^**2**^ **)**				12 (1.47%)
**<16**	40 (5.0%)	25 (6.2%)	15 (3.7%)	
**16 - <18.5**	146 (18.1%)	75 (18.6%)	71 (17.7%)	
**18.5 - < 25**	507 (62.9%)	251 (62.1%)	256 (63.7%)	
**25 - < 30**	91 (11.3%)	41 (10.1%)	50 (12.4%)	
**≥ 30**	22 (2.7%)	12 (3.0%)	10 (2.5%)	
**Counseling & Testing/Point of Entry**				0 (0%)
**VCT**	408 (49.9%)	207 (50.6%)	201 (49.1%)	
**PITC**				
***DTC***	126 (15.4%)	64 (15.6%)	62 (15.1%)	
***OPD***	83 (10.1%)	39 (9.5%)	44 (10.7%)	
***In-patient Wards***	71 (8.7%)	34 (8.3%)	37 (9.0%)	
***PMTCT***	16 (1.9%)	8 (1.9%)	8 (1.9%)	
***Tb Clinic***	11 (1.3%)	4 (1.0%)	7 (1.7%)	
***Other PITC***	62 (7.6%)	31 (7.6%)	31 (7.6%)	
**Other**	41 (5.0%)	22 (5.4%)	19 (4.6%)	
**Education Level**				0 (0%)
**None**	21 (2.6%)	8 (1.9%)	13 (3.2%)	
**Primary**	457 (55.9%)	239 (58.4%)	218 (53.3%)	
**Secondary**	291 (35.6%)	136 (33.2%)	155 (37.9%)	
**Tertiary**	49 (6.0%)	26 (6.4%)	23 (5.6%)	
**Marital Status**				0 (0%)
**Single**	94 (11.5%)	45 (11.0%)	49 (11.9%)	
**Married monogamous**	442 (54.0%)	230 (56.2%)	212 (51.8%)	
**Married polygamous**	66 (8.1%)	33 (8.1%)	33 (8.1%)	
**Cohabitating**	29 (3.6%)	16 (3.9%)	13 (3.2%)	
**Separated**	70 (8.6%)	31 (7.6%)	39 (9.5%)	
**Divorced**	10 (1.2%)	6 (1.5%)	4 1.0%)	
**Widowed**	107 (13.1%)	48 (11.7%)	59 (14.4%)	
**Discordant Couple**				0 (0%)
**Yes**	59 (7.2%)	27 (6.6%)	32 (7.8%)	
**No**	315 (38.5%)	156 (38.1%)	159 (38.8%)	
**Unknown**	444 (54.3%)	226 (55.2%)	218 (53.3%)	
**History of Tb Treatment**				0 (0%)
**Yes**	46 (5.6%)	29 (7.1%)	17 (4.2%)	
**No**	763 (93.3%)	374 (91.4%)	389 (95.1%)	
**Unknown**	9 (1.1%)	6 (1.5%)	3 (0.7%)	
**History of Cryptococcus Treatment**				0 (0%)
**Yes**	3 (0.4%)	2 (0.5%)	1 (0.3%)	
**No**	806 (98.5%)	401(98.0%)	405 (99.0%)	
**Unknown**	9 (1.1%)	6 (1.5%)	3 (0.7%)	
**Current TMP/SMX prophylaxis**				0 (0%)
**Yes**	796 (97.3%)	396 (96.8%)	400 (97.8%)	
**No**	9 (1.1%)	6 (1.5%)	3 (0.7%)	
**Unknown**	13 (1.6%)	7 (1.7%)	6 (1.5%)	
**Current Tb Treatment**				0 (0%)
**Yes**	67 (8.2%)	36 (8.8%)	31 (7.6%)	
**No**	738 (90.2%)	366 (89.5%)	372 (90.9%)	
**Unknown**	13 (1.6%)	7 (1.7%)	6 (1.5%)	
**HgB (g/dL)**				161 (19.68%)
**Grade 0 /Normal**	467 (71.4%)	239 (73.3%)	228 (69.5%)	
**Grade 1**	129 (19.7%)	58 (17.8%)	71 (21.6%)	
**Grade 2**	30 (4.6%)	15 (4.6%)	15 (4.6%)	
**Grade 3**	15 (2.3%)	9 (2.8%)	6 (1.8%)	
**Grade 4**	13 (2.0%)	5 (1.5%)	8 (2.4%)	
**ALT/AST**				173 (21.15%)
**Grade 0 /Normal**	618 (95.8%)	302 (95.0%)	316 (96.6%)	
**Grade 1**	21 (3.3%)	12 (3.8%)	9 (2.8%)	
**Grade 2**	6 (0.9%)	4 (1.2%)	2 (0.6%)	
**Grade 3**	0	0	0	
**Grade 4**	0	0	0	
**Creatinine (u mol/L)**				161 (19.68%)
**Grade 0 /Normal**	564 (88.5%)	282 (89.5%)	282 (87.6%)	
**Grade 1**	30 (4.7%)	13 (4.1%)	17 (5.3%)	
**Grade 2**	24 (3.8%)	11 (3.5%)	13 (4.0%)	
**Grade 3**	15 (2.4%)	8 (2.5%)	7 (2.2%)	
**Grade 4**	4 (0.6%)	1 (0.3%)	3 (0.9%)	
**ART Regimen Initiated**				0 (0%)
**D4T/3TC/NVP**	234 (28.6%)	118 (28.8%)	116 (28.4%)	
**D4T/3TC/EFV**	80 (9.8%)	44 (10.8%)	36 (8.8%)	
**AZT/3TC/NVP**	245 (30.0%)	120 (29.3%)	125 (30.6%)	
**AZT/3TC/EFV**	162 (19.8%)	81 (19.8%)	81(19.8%)	
**TDF/3TC/NVP**	47 (5.7%)	22 (5.4%)	25 (6.1%)	
**TDF/3TC/EFV**	50 (6.0%)	24 (5.9%)	26 (6.4%)	

Notes:

1. Data presented as mean (SD) or n (%)

2. Two participants who were randomized but did not continue ART are excluded.

3. Categorical grades based upon the DAIDS Toxicity tables: Grade 1 = mild, Grade 2 = moderate, Grade 3 = severe, Grade 4 = potentially life-threatening

4. Abbreviations: VCT = Voluntary Counseling and Testing, DTC = Diagnostic Testing and Counseling, PITC = Provider Initiated Testing and Counseling; Tb = tuberculosis, PMTCT = Prevention of Mother to Child Transmission, TMP/SMX = trimethoprim/sulfamethoxazole, D4T = stavudine, 3TC = lamivudine, NVP = nevirapine, EFV = efavirenz, AZT = zidovudine, TDF = tenofovir

### Monitoring and Data Quality

Prior to full study accrual, the DMC met 3 times with no data or safety concerns. In addition, each site was monitored at least once with 100% informed consent review. Through enrollment and full accrual, 100% informed consent compliance was documented and two protocol violations occurred. During a period of new guidelines being introduced, two participants (0.2%) started ART with higher CD4 counts who did not meet MoH guidelines but were enrolled and ineligible. Both had ART discontinued, continued routine clinic follow-up and including trimethoprim/sulfamethoxazole prophylaxis and multivitamin supplementation and were excluded from analysis.

Complete baseline CD4+ T-cell and VL (Arm B only) data were obtained and available. At enrollment, all Arm A participants had VLs received in the lab for protocol-defined retrospective analyses. Throughout enrollment, all sites passed quarterly CD4+/CD3+ T cell count external quality assurance (EQA) testing. While one site failed 2 successive EQA tests, it was discovered upon corrective action review that the site transcribed results on the EQA report incorrectly (i.e. CD4+ for CD3+ and vice-versa) and had actually passed after correcting for transcription error. Capture of data linked to secondary CRFs (e.g. aminotransferase/aspartate aminotransferase, hemoglobin, and creatinine) was approximately 80%.

## Discussion

The CLADE trial demonstrates a RCT can be designed and fully enrolled under current Good Clinical Practices (GCP) in a predominately rural, resource limited setting by leveraging upon local capacity and infrastructure. For best generalizability regarding the feasibility, superiority, and cost-effectiveness of routine VL monitoring, the question must be studied in the most relevant clinic setting. Limitations in extrapolating findings generated within the context of a research setting are well described in resource rich settings based upon the differences in care[42, 43] The magnitude of research vs. clinic setting differences are most likely greater in resource limited settings. CLADE provides a clinical trial model for consideration as the role of implementation science expands under PEPFAR and other donor programs.[44]

Data generated from the baseline cohort provide unique information and important opportunities for prevention, care, and treatment interventions. To our knowledge, CLADE offers among the first clinical trial data systematically collected in rural, non-research clinics of Kenya. Furthermore, approximately five years after care and treatment programs were implemented, adults presenting for first line ART have notably advanced HIV/AIDS. Half of those asked regarding the HIV status of their partner reported that they were unaware. These findings emphasize the importance of continued universal HIV testing and linkage to care campaigns as well as couples HIV counseling and sharing of HIV status.

The greatest strengths of CLADE are the design (providing among the strongest quality of data/evidence) and setting (non-research, clinic-based, largely rural where a notable HIV burden exists). With such a RCT never being conducted in rural Kenya (and likely elsewhere in sub-Saharan Africa) before, questions existed regarding the feasibility and practicality of conducting CLADE in non-research, rural, district level facilities. We note evaluating the feasibility of conducting such a trial in this setting was a secondary objective. Concerns related to Good Clinical Practice (GCP) such as informed consent, randomization, and data quality as well as others were found not to be troublesome in the accrual and baseline enrollment. In addition, CLADE has been from its concept largely “owned” by Kenyans with a secondary, supportive role from non-national team members. Last, CLADE leverages largely upon local capacity and infrastructure with strong “buy-in” and support from the MoH, faith-based institutions, and CAB, which should be an expected norm for such research.

The primary limitation is the inability to draw conclusions regarding secondary endpoints including mortality. By design, the CLADE sample size focuses upon the question of superiority routine VL monitoring. We believed that routine viral monitoring would identify patients with detectable viral loads earlier than immunologic and clinical monitoring resulting in earlier adherence interventions ultimately resulting in more long standing viral suppression. We felt 18 months follow-up (3 series of 6 month routine viral load monitoring) was clinically relevant for comparing routine care versus viral load monitoring. It is appreciated that results from secondary endpoints such as mortality will be largely descriptive in nature. Therefore, CLADE will not be able to draw conclusions regarding the relationships between routine VL monitoring and death. However, the relationship between viral failure and subsequent morbidity and mortality is well established.[45, 46] While nearly all data related to the primary objectives and VL endpoint (e.g. CD4 count, VL >98%) were collected, we captured a smaller proportion (approximately 80%) for clinical laboratory evaluations. This reflected the “secondary” data collection approach agreed upon in planning with MoH and FBO implementing partners and suggests the need for additional dedicated staff and funding for future clinic-based clinical trials in such settings.

While CLADE is unique in methods utilized, additional trials evaluating HIV monitoring approaches in differing populations and settings in Africa exist [47–50]. All are important in providing data to inform stakeholders developing future policies and guidelines.

In conclusion, CLADE provides important experience and data regarding the design and successful enrollment of a RCT under GCP in a rural, non-research, resource limited setting. Important opportunities exist for prevention, care, and treatment programs. Key information to come for clinical care and research programs as well as policy makers will be feasibility, superiority, and cost implications of routine VL monitoring.

## Supporting Information

S1 CONSORT Checklist CLADE Consort Checklist. (DOCX)

S1 CONSORT Diagram CLADE Consort Diagram. (TIF)

S1 Consent CLADE ENGLISH ICF_v 2–0 13-February 2012. (PDF)

S2 Consent CLADE KISWAHILI ICF_v 2–0 13-Feb 2012. (PDF)

S3 Consent CLADE DHOLUO ICF v 2_13 February 2012. (PDF)

S1 Protocol CLADE Protocol v2–5_30NOV12. (PDF)

S1 Appendices CLADE Appendices 30_Nov_2012. (PDF)
