# Supplementary figures and images for: Kericho CLinic-Based ART Diagnostic Evaluation (CLADE): Design, Accrual, and Baseline Characteristics of a Randomized Controlled Trial Conducted in Predominately Rural, District-Level, HIV Clinics of Kenya
Source: PLoS One. 2015 Feb 23;10(2):e0116299. doi: 10.1371/journal.pone.0116299 (PMC4338154; doi:10.1371/journal.pone.0116299)

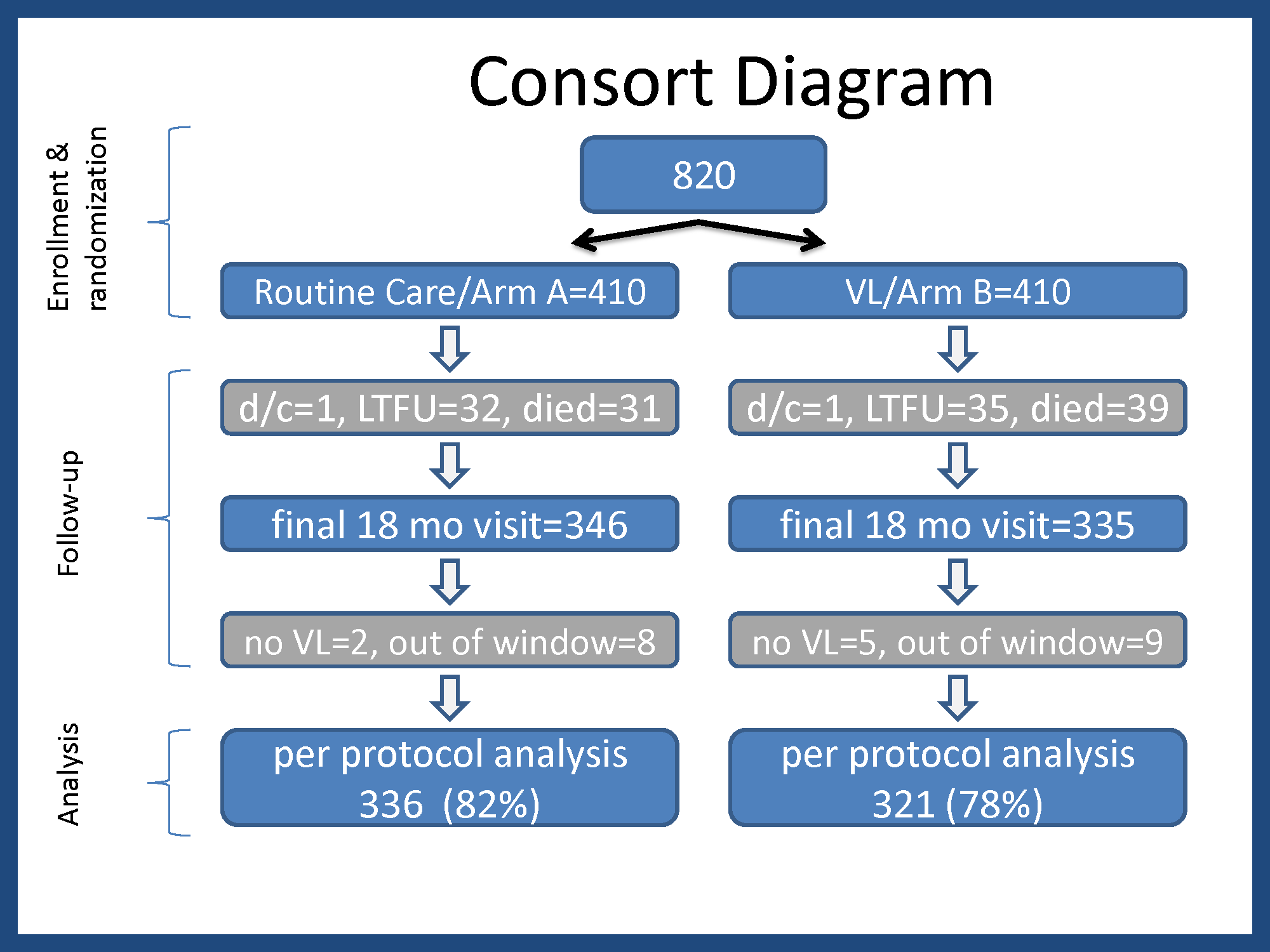

Supplement: S1 CONSORT Diagram — (TIF) [file pone.0116299.s002.tif]
