# Supplementary material for: Kericho CLinic-Based ART Diagnostic Evaluation (CLADE): Design, Accrual, and Baseline Characteristics of a Randomized Controlled Trial Conducted in Predominately Rural, District-Level, HIV Clinics of Kenya
Source: PLoS One. 2015 Feb 23;10(2):e0116299. doi: 10.1371/journal.pone.0116299 (PMC4338154; doi:10.1371/journal.pone.0116299)
Supplement: S1 Consent — (PDF) [file pone.0116299.s003.pdf]

INFORMED CONSENT FORM

THE KENYA MEDICAL RESEARCH INSTITUTE (KEMRI) /
WALTER REED ARMY INSTITUTE OF RESEARCH (WRAIR)/
WALTER REED PROJECT (WRP)

CLINIC-BASED ART DIAGNOSTIC EVALUATION (CLADE)

PUBLIC HEALTH OUTCOMES RESEARCH

Version 2.0 February 13, 2012

INTRODUCTION

This is a consent form for a research study being conducted by the Kenya Medical Research Institute (KEMRI) and Walter Reed Army Institute of Research (WRAIR)/Walter Reed Project (WRP). The study is called, "CLADE", which stands for "**CL**inic-based **A**RT **D**iagnostic **E**valuation."

The CLADE study is sponsored and paid for by the US Office of the Global AIDS Coordinator (O-GAC) / President's Emergency Plan for AIDS Relief (PEPFAR) program. Doctors leading this study at this site are *Dr Fredrick Sawe (telephone numbers: 052-30388; 0724-255-623)* and *Dr. Jonah Maswai (052-30388; 0716-430-217)* of the Kenya Medical Research Institute/Walter Reed Project Clinical Research Center (Hospital Road, PO Box 1357, Kericho-20200, Kenya).

You are being asked to take part in this research study because you are attending an HIV clinic and will be receiving antiretroviral therapy (ART) to manage HIV/AIDS. Before you decide if you want to take part in this study, we want you to know about the study.

This consent form gives you information about this study. The research staff will talk with you about this information. You are free to ask questions about this study at any time. If you agree to take part in this study, you will be asked to sign this consent form and get a copy to keep.

Please know that:

- Participation in this study is entirely voluntary.
- You may stop taking part in the study at any time.
- You will still receive your ART at this clinic like everyone else even if you do not participate in this study.

WHY IS THIS STUDY BEING DONE?

The purpose of this study is to look at two ways the Ministry of Health (MOH) recommends to follow patients receiving ART. One way is by your doctor examining you and checking your CD4 count with targeted viral load test. Another way is by your doctor examining you, checking your CD4 count, and doing a test to check your HIV viral load routinely. Both ways help show how well your ART is working. CD4 cell

counts are checked as part of your routine care at this clinic every 6 months or as your doctor feels necessary. Viral loads are recommended by the MOH but are not checked as part of your routine care at this clinic because of the costs of the test and the equipment is not readily available.

We are not sure if doing the viral load every time your CD4 count is checked will help your doctor in knowing if your ART is working well. That is a reason why this study is being carried out

WHAT IS EXPECTED OF ME IN THIS STUDY?

To join this study, you must be 18 years of age or older. Both males and females can participate. Pregnant women will not be able to join the study, but if a woman becomes pregnant during the study and her doctor decided to continue ART, she may continue on the study. If you decide to join this study, you will need to be sure to come for all of your HIV clinic appointments. This is very important whether or not you decide to be in the study. If you enter the study, you will be expected to come to the clinic for your regularly scheduled ART follow-up. This is about twice a month for the first month. Then, your doctor will likely ask you to come every month for several months. Once you have been on your ART and are doing well, your doctor can increase the time you come back to every 3 to 6 months. How often you come to the HIV clinic can change if you are ill or if your doctor thinks you need to come back more or less. Staying healthy is the main reason you should come back when your doctor asks. Coming back for your appointments is also important for this study because the study team needs to collect information from your medical record to see how you are doing.

You shouldn't have to spend too much extra time at any visit if you participate in this study. You will need to tell the clinic nurse you are in the study when you arrive. Otherwise, your medical care will be the same if you were in the study or not. The study team will review your records after your visit is over. Information the study team will collect from your medical record will include only information that usually is recorded on your medical record (but not your name or your contact information). Examples include how well you feel, your weight, your education level, any illnesses you develop, any admissions to the hospital, how well you are taking your ART and other HIV medicines, lab results, and any medicines or lab tests your doctor may order.

The research team will ensure that any information collected will be kept confidential. To enable us to do this, we will assign you a specific study identification number (SID) that will be used when we collect the information from your treatment file. We will use this number instead of your name when we collect information from your medical record.

At the beginning of the study, you will be assigned to either the ART monitoring that uses only clinical examination and CD4 count with targeted viral load (called "Group A") or the monitoring that uses clinical examination, CD4 count, and the viral load test (called "Group B"). If you are in Group A, you will be examined and get a CD4 count every 6 months (or when your doctor thinks it is necessary) with targeted viral load done

if you are failing treatment. If you are in Group B, you will be examined and get a CD4 count and viral load every six months (or when your doctor thinks it is necessary).

You have equal chance of being in either group, like the flip of a coin. You, your doctor, and the study team will know what group you have been assigned. Neither you, your doctor, nor the research team can change the group once you are assigned.

Please understand too that if you are in the group that does not use viral load (Group A) but your doctor thinks you need a viral load test because he or she thinks your ART may not be working, your doctor can order a viral load test. This will not be done routinely if you are in this group that does not usually order a viral load. If your doctor orders a viral load, you will be told the result.

There are two types of tests that will be done as part of research that are explained below.

1. If you are assigned to the group using clinical examination and CD4 count to follow your ART (Group A), the research team will keep about 5 mls (about a teaspoonful) of blood at the KEMRI/WRP CRC Kericho each time your doctor orders a CD4 count. This should be about once every six months. Within 6 months of the study being over, the lab will use this blood to check and see what the viral load was when you got your CD4. This will also help the researchers see if getting viral loads the same time your doctor orders a CD4 is helpful. Once these tests are run, the results will be given to your doctor who can discuss them with you.

2. For persons in both groups, the research team will keep about 5 mls (about a teaspoonful) of blood at the KEMRI/WRP CRC Kericho when you have laboratory tests run 3 months after starting ART. Within 6 months of the study being over, the lab will use this blood to check and see how much of your HIV virus was suppressed by your ART. This will also help the researchers know how well ART works the first 3 months after starting. Once this test is run, the results will be given to your doctor who can discuss them with you.

No blood will be kept after 6 months from when the study is over and all regular information has been collected from your medical record.

For all persons in this study, your doctor will be able to check to see if you have developed resistance and sub-type diversity to any drug used to treat your HIV. Resistance means that one or more of your drugs to treat HIV does not work. That is, the virus has become "resistant." This resistance testing and sub-type diversity will be done if your doctor feels the ART started is not working and he or she wants to change your ART to a second type of ART. If this is done, your doctor will share the results of this resistance testing and sub-type diversity with you. Also at the end of the study, your doctor will check for resistance to your ART if it is shown that your ART has not suppressed your HIV virus.

Finally, the research team will keep about 5 mls of your blood (about a tea spoon) before you start your ART to see if some of those starting ART have resistance. For this time when we check to see if there is resistance before you start ART, not everyone will have this test done. If a resistance test is done on your blood before you start ART, you will be given the results once they are available. However, it is important to note that this testing will not be done for some time after the study has started.

HOW MANY PEOPLE WILL TAKE PART IN THIS STUDY?

It is thought that about 820 people will take part in this study at 7 ART treatment sites in the Rift Valley and Nyanza Provinces.

HOW LONG WILL I BE IN THIS STUDY?

Once you start ART, we will collect information from your medical records for about 1 and ½ years. This may be a couple of months longer depending upon when your doctor asks you to come back for usual visits. The KEMRI and WRAIR will own the information collected in this study.

Once we stop collecting information and the study is over, you will continue your regular ART care.

REASONS FOR EARLY STUDY DISCONTINUATION

You may be taken off this study early for any of the following reasons:

1. At your own request to stop participating in this study.
2. Your primary doctor or the HIV clinic staff feel the study is no longer in your best interests.

Organizations overseeing this study such as the Institutional Review Boards (KEMRI and/or WRAIR), the Kenya Ministry of Health (MOH), or the study sponsor (Office of the Global AIDS Coordinator/PEPFAR) feel the study should be stopped all together.

A group called the “Data Monitoring Committee” will review progress of the study. That is, they will look at information being collected from your records. This committee will evaluate study progress such as how many persons are enrolling in the study; how many persons may have viral load failures; how many persons may be noted by their doctors to have treatment failure; and, other factors. This committee will not receive any information with participant names. About half way through the study, the committee will look to see if one of the ways of monitoring (Group A or Group B) appears to be better than the other. If one is found better half way through the study, then the committee may recommend that the study be stopped. If this happens, you will be informed of the findings. You will continue to receive care provided by the ART clinic just the same.

WHAT ARE THE RISKS IN THIS STUDY?

Your doctors and the HIV clinic staff will explain to you the risks involved in taking ART. Those risks are present whether you enter the study or not. There are some risks involved in participating in this study. However, your doctor and the research team will do everything to minimize the chances of these risks affecting you. There could be the possible social risk of stigma around HIV and AIDS. Fortunately, your treatment site has been running for at least 3 years and has an identifiable HIV clinic. So, social harm to you from HIV /AIDS related stigma is much less than previously when ART was not easily available. Just like for all patients in the ART clinic, your doctors and health care workers will be sensitive to stigma around HIV/AIDS. If you feel at any time you have been harmed in any way (social or physical), you should let your doctor or the HIV clinic staff or the CLADE study research team know.

There is a chance that you may be harmed any time blood is taken for your laboratory tests. These include discomfort, bleeding or bruising where the needle enters the body, light headedness, and in rare cases fainting or infections. This is the case even if you are not in this study.

Finally, there is a chance that information from your clinical record could be exposed in the process of study data collection. While the team will never use your name or address, we can not guarantee this would never happen at the clinic. Also, we will transport the information from your clinical record to the KEMRI/WRP research center next to Kericho District Hospital. With exception of the information on registration and consenting, we will only transport this on a computer without your name or address. Once the information is received at our research center, we will erase it from the computer used to transport the information. The initial information containing your name and other identifiers taken on the first visit will be stored in a separate database that is password protected with limited access to only the IT manager, his deputy and under the discretion of the PI.

ARE THERE BENEFITS TO TAKING PART IN THIS STUDY?

It is possible that you may receive no benefit from being in this study. If you participate in this study, there may be some benefit to you, but there is no guarantee. By participating in this study, you may learn more about your HIV monitoring than usual. For example, viral loads or resistance testing may not routinely be done. While we are not certain and are trying to better understand from the study, it is possible that you may have a better response to your ART if you have routine viral load monitoring. Lastly, by participating in the study, you may have viral load and resistance testing, which can help in knowing how best to select your ART.

WHAT OTHER CHOICES DO I HAVE BESIDES THIS STUDY?

Participation in this study is entirely voluntary. You can choose not to participate now or anytime later if you decide to participate. You will still get the same ART at this

treatment site like everyone else. Please talk to your doctor about the choices available to you.

WHAT ABOUT CONFIDENTIALITY?

The study team will provide you with a specific study identification number. This identification number (not your name or other information that could be used to identify you) will be used for collecting all information from your clinical record. Your study records will be kept in a locked room. Only the study staff will have the keys. Any publication of this study will not use your name or identify you personally.
Efforts will be made to keep your personal information confidential, but we cannot absolutely guarantee this. Your personal information may be disclosed if required by law. Your records may also be reviewed by the study sponsor, Department of Defense (DoD) / Walter Reed Army Institute of Research (WRAIR), or United States Army Medical Research and Material Command (USAMRMC) Office of Research Protection (ORP) Human Research Protections Officer (HRPO), Kenya Medical Research Institute (KEMRI) or other authorized individuals as outlined in Kenya and US policies.

WILL I RECEIVE ANY PAYMENT?

The leadership at your hospital will decide if you receive compensation for participating in this study based upon practices at your hospital. If the leadership decides that participants in CLADE will receive compensation, you will receive compensation for travel and any additional time that may be imparted by study participation. At the end of each routine clinical/study visits you will receive 400 -500 KSH. For any unscheduled visit, you will receive 200 KSH. The clinic may decide that funds for participation will be kept in order to make improvements in the HIV clinic for all patients. If so, you will not receive compensation for participating in CLADE.

WHAT ARE THE COSTS TO ME?

This study will not levy any charge when you agree to join it and allow the research team to collect information from your records. Please note that the CLADE study will not assume the responsibility of providing you with care and treatment. The cost of your care and treatment will remain your duty or your insurance company, or your health care system. The study will not provide you with funds for buying of anti-HIV drugs or offsetting other unforeseen costs that come up in the course of your HIV treatment. However what we can assure you is that no cost will be passed to you for extra study tests or tracking the results and delivering them to your doctor to use in your treatment.

Please note that your treatment facility may have some charges like patient registration and facility maintenance, these will usually be paid by all persons seeking treatment at that facility including those receiving ART. It will still remain your responsibility to pay such fees.

WHAT HAPPENS IF I AM INJURED?

If you are injured as a result of being in this study, you will be provided free emergency medical care only for that injury. There is no plan for compensation for illness or injury either through the Kenya Medical Research Institute, the Walter Reed Army Institute of Research, or the PEPFAR program. You will be given information on where to get further treatment if needed. You should also understand that this is not a waiver or release of your legal rights. You should discuss this issue thoroughly with the study team before you enroll in this study.

If you are hurt or get sick because of this research study, you can receive medical care at your treatment hospital. You will only be treated for injuries that are directly caused by the research study. The Kenya Medical Research Institute, Walter Reed Army Institute of Research, or PEPFAR program will not pay for your transportation to and from the hospital or clinic.

If you have questions about this medical care, talk to Dr. Fredrick sawe *(telephone numbers: 052-30388; 0724-255 623)* or Dr. Jonah Maswai *(telephone numbers: 052-30388; 0716-430 271).* If you pay out-of-pocket for medical care elsewhere for injuries caused by this research study, contact the Principal Investigator. If the issue cannot be resolved, contact the KEMRI IRB administrator on telephone 020-2722541 or the U.S. Army Medical Research and Materiel Command (USAMRMC) Office of the Staff Judge Advocate (legal office) at (301) 619-7663/2221.

WHAT IS MY RIGHT AS A VOLUNTEER IN A RESEARCH STUDY?

Taking part in this study is completely voluntary. You may choose not to take part in this study or leave this study at any time. You will be treated the same no matter what you decide. We will tell you about new information from this or other studies that may affect your health, welfare, or willingness to stay in this study. If you want the results of the study, please let the study staff know.

WHAT IF I HAVE QUESTIONS OR PROBLEMS?

For any questions about the study or any injuries arising from your participation in the study contact the study medical doctor/researcher:

Dr Fredrick Sawe
Kenya Medical Research Institute/Walter Reed Project Clinical Research Center,
Hospital Road
PO Box 1357,
Kericho-20200, Kenya
Tel: (254-52) 30388/32101
Mobile: (254) 724-255 623
Fax: (254-52) 30662 / 30546
Email: fsawe@wrp-kch.org

In case of any emergencies you may contact your doctor, the HIV clinic staff or the CLADE study doctor on telephone number 0723-226-229 (the emergency line is open 24 hrs). If you cannot reach any of the researchers on the numbers provided above, for any emergencies you may contact Rither Langat the study co-ordinator *(telephone numbers: 052-30388; 0713 603289).*

For any questions about your rights as a participant in the study or any complaints about the study, the KEMRI IRB administrator can be reached on telephone 020-2722541.

SIGNATURE PAGE

If you have read this consent form (or had it explained to you), all your questions have been answered, and you agree to take part in this study, please sign your name below.
A copy of this informed consent will be given to you.

______________________________	______________________________
Participant’s Name (print)	Participant’s Signature or Left Thumb print and Date

____

Participant’s Address

______________________________	______________________________
Study Staff Conducting Consent Discussion (print)	Study Staff Signature and Date
______________________________	______________________________
Witness’s Name (print) (As appropriate)	Witness’s Signature and Date
