# Supplementary material for: Kericho CLinic-Based ART Diagnostic Evaluation (CLADE): Design, Accrual, and Baseline Characteristics of a Randomized Controlled Trial Conducted in Predominately Rural, District-Level, HIV Clinics of Kenya
Source: PLoS One. 2015 Feb 23;10(2):e0116299. doi: 10.1371/journal.pone.0116299 (PMC4338154; doi:10.1371/journal.pone.0116299)
Supplement: S2 Consent — (PDF) [file pone.0116299.s004.pdf]

TAASISI YA UTAFITI WA KIMATIBABU YA KENYA (KEMRI) / TAASISI YA JESHI LA WALTER REED (WRAIR)/ MRADI WA WALTER REED (WRP)

MAKADIRIO YA MATIBABU YA ART KATIKA KLINIKI (CLADE)

Matokeo ya utafiti wa afya ya uma

Toleo la 2.0 Februari 13, 2012

UTANGULIZI

Hii ni fomu ya idhini ya mradi wa utafiti unaoendelezwa na Taasisi ya Utafiti wa Kimatibabu ya Kenya (KEMRI) na Taasisi ya Jeshi la Walter Reed (WRAIR)/ Mradi wa Walter Reed (WRP). Mradi huu unaitwa "CLADE", ambao unamaanisha "Makadirio ya Matibabu ya ART katika Kliniki."

Mradi wa CLADE unafadhiliwa na kugharamiwa na Afisi ya Marekani inayoelekeza mambo kuhusu UKIMWI duniani (O-GAC)/ Mradi wa Raisi wa Mpango wa Dharura katika kufariji mambo ya UKIMWI (PEPFAR). Madaktari wanao uongoza utafiti huu eneo hili ni *Dkt. Fredrick Sawe (nambari ya simu: 052-30388;0724-255-623) na Dkt Jonah Maswai(nambari ya simu:052-30388;0716-430-217)* wa Taasisi ya Utafiti wa Kimatibabu ya Kenya/Kikao cha Utafiti wa Kimatibabu cha Walter Reed (Hospital Road, PO Box 1357, Kericho-20200, Kenya)

Unaombwa kushiriki katika utafiti huu kwa sababu unahudhuria kliniki ya HIV na unapokea matibabu ya (ART) yanayo kabiliana naKIMWI. Kabla ya kufanya uamuzi wa kushiriki katika utafiti huu, tungependa kukupasha habari kamili kuhusu utafiti.

Hii fomu ya idhini inakupa habari kuhusu utafiti huu. Wahudumu katika utafiti watazungumza nawe kuhusu habari hii. Uko huru kuuliza maswali kuhusu utafiti huu wakati wowote. Ikiwa utakubali kushiriki katika utafiti huu, utahitajika kutia sahihi fomu hii ya idhini na utapata nakala ya kujiwekea.

Tafadhali elewa ya kuwa:

- Kushiriki katika utafiti huu ni kwa hiari yako
- Unaweza kuwacha kushiriki katika utafiti wakati wowote
- Utaendelea kupokea matibabu ya ART katika kliniki hii kama mtu yeyote yule hata kama hutashiriki katika utafiti huu

NI KWA NINI UTAFITI HUU UNAFANYWA?

Sababu ya utafiti huu ni kutazama njia mbili zinazopendekezwa na Wizara ya Afya kwa kufuatilia watu wanaopokea matibabu ya ART. Njia moja ni daktari wako kufanya uchunguzi na kipimo cha hesabu yako ya CD4 kinachozingatia matukio muhimu ya

kiafya. Njia nyingine ni daktari kukufanyia uchunguzi, kuangalia hesabu ya chembechembe za CD4 na kuendeleza utaratibu wa vipimo ili kutathmin kiwango cha virusi vya HIV kama inavyofanyika kawaida. Njia hizi mbili husaidia katika kuonyesha utendakazi wa ART. CD4 huchunguzwa kama taratibu ya utunzaji katika kliniki hii kila miezi 6 au kama daktari yako ataona kuwa sawa. Kiwango cha virusi kinapendekezwa na Wizara ya Afya lakini uchunguzi haufanywi kama taratibu ya utunzaji katika kliniki hii kwa sababu ya gharama ya vipimo hivi na vifaa ambazo ni nadra kupatikana.

Hatuna uhakika kama kwamba kuchunguza kiwango cha virusi kila wakati hesabu ya CD4 inapofanywa itasaidia daktari katika kutambua ama utendakazi wa ART ni sawa. Hii ndiyo sababu utafiti huu unaedelezwa.

NI LIPI LINAHITAJIKA KUTOKA KWANGU KATIKA UTAFITI HUU?

Kwa kushiriki kwa utafiti huu, lazima uwe na miaka 18 au zaidi.Wanaume na wanawake wanaweza kushiriki.Wanawake wajawazito hawatashiriki kwa utafiti huu lakini mwanamke akishika mimba wakati wa utafiti na daktari wake aamue kuendeleza dawa ya ART ,ataendelea kushiriki kwa utafiti. Iwapo utajiunga katika utafiti utahitajika kuja katika kliniki kwa ufuatilizi wa ratiba yako ya ART kama ilivyoorodheshwa. Hiinni takriban mara mbili katika mwezi wa kwanza. Kisha daktari wako atakuomba kuja kliniki kila mwezi kwa miezi kadhaa. Mara unapokuwa katika mpango wa ART na unaendelea vyema, daktari wako anaweza kuongeza muda wa kurejea katika kliniki kuwa kila miezi 3 – 6. Takriban ya ziara unazofanya katika kliniki ya HIV inaweza kubadilika iwapo utaugua au iwapo daktari wako atakuhitajika kuja ziara nyingi zaidi au chache. Kuendelea katika hali njema ya afya ni mojawapo ya sababu yako kurejea unapoombwa na daktari wako. Kurejea kwa miadi yako pi ni ya muhimu kwa utafiti huu kwa vile timu ya utafiti inahitaji kuzua habari kutokana na rekodi zako za kimatibabu kuangalia jinsi unavyoendelea.

Huatahitajika kutumia muda mwingi zaidi katika kila ziara iwapo utaamua kushiriki katika utafiti huu. Utahitajika kumjulisha muuguzi katika kliniki hii kuwa wewe ni mshiriki mara unapofika. Hata hivyo, huduma zako za kimatibabu zitakuwa sawa ikiwa unashiriki au hata kama wewe sio mshiriki. Timu ya utafiti itapitia rekodi zako baada ya ziara yako kukamilika. Habari timu ya utafiti itazua kutoka katika rekodi zako za kimatibabu ni ile tu inayorekodiwa katika rekodi zako za kimatibabu (lakini sio jina lako wala habari za mawasiliano). Mifano ni kama vile jinsi unavyohisi, uzito wako, kiwango cha elimu, ugonjwa wowote unaozuka, kulazwa hospitalini, jinsi unavyofuatiliza matibabu yako ya ART na madawa mengineyo ya HIV, matokeo ya mahabara, na madawa yoyote au vipimo vyovyote vilivyoagizwa na daktari wako.

Timu ya utafiti itahakikisha kuwa habari zozote zitahifadhiwa kisiri. Ili kuwezesha hili kufanyika, utapewa nambari maalum ya kukutambua ya utafiti itakayotumiwa katika kuzua habari kutoka faili yako ya matibabu. Hii ndiyo number itakayotumika badala ya jina lako tutakapozua habari kutoka kwa rekodi zako za kimatibabu.

FOMU YA IDHINI

Hapo mwanzo wa utafiti, utaelekezwa aidha kwa ufuatilizi wa ART unaotumia uchunguzi wa kimatibabu na kipimo cha hesabu ya CD4 kinachozingatia matukio muhimu ya kiafya (kikundi A) au ufuatilizi unaotumia uchunguzi wa kimatibabu, hesabu ya CD4 na pia vipimo vya kiwango cha virusi kama inavyofanyika kawaida (kikundi B). Iwapo u katika kukundi cha A, utafanyiwa uchunguzi na pia kipimo cha hesabu ya CD4 kinachozingatia matukio muhimu ya kiafya iwapo matibabu yanafeli. Hili litafanywa kila baada ya miezi 6 (au kama daktari wako anavyoonelea kuwa fasaha). Iwapo u katika kikundi cha B, utafanyiwa uchunguzi na pia hesabu ya chembechembe za CD4 na kiwango cha virusi kufanywa kila baada ya miezi 6 (au kama daktari wako anavyoonelea kuwa fasaha).Utapata nafasi sawa ya kuwa aidha katika kikundi chochote kile, kama vile kurusha kwa sarafu. Wewe, daktari wako na mhudumu katika timu ya utafiti mtatambua ni kikundi kipi ulichoelekezwa Lakini wewe, daktari wako au timu ya utafiti hana uwezo wa kubadili nafasi uliyoelekezwa.

Tafadhali elewa pia, kuwa iwapo utakuwa katika kikundi ambacho hakitumii kiwango cha virusi (Kikundi A) lakini daktari wako anafikiria kuwa unahitaji vipimo vya kiwango cha virusi kwa ajili anafikiria kuwa utendakazi wa ART sio sawa, daktari wako anaweza kuagiza vipimo vya kiwango cha virusi. Hili halitafanywa kama taratibu lakini tu iwapo u katika kikundi hiki ambacho kwa kawaida hakitumii kiwango cha virusi. Iwapo daktari wako ataagiza kufanyiwa vipimo vya kiwango cha virusi, basi utajulishwa matokeo yake.

Ni aina mbili ya vipimo itakayofanywa katika utafiti huu kama ilivyoelezewa hapo chini.

1. Iwapo utaelekezwa katika kikundi kinachotumia utunzaji wa kimatibabu na hesabu ya CD4 kwa ufuatilizi wa ART (kikundi A), timu ya utafiti itahifadhi takriban mililita 5 (kijiko kimoja cha chai) ya damu pale KEMRI/WRP CRC Kericho kila wakati daktari wako anapoagiza hesabu ya chembechembe za CD4. Hii ni karibu mara moja kwa kila miezi sita. Katika kipindi cha miezi sita baada ya utafiti kukamilika, mahabara itatumia damu hii kuchunguza kiwango cha virusi wakati ulipofanyiwa hesabu ya CD4. Hii pia itasaidia watafiti kutambua iwapo kutambua kiwango cha virusi wakati daktari anapoagiza hesabu ya CD4 kufanywa ni ya muhimu. Japo vipimo hivi vinapofanywa, matokeo yatapokezwa kwa daktari wako anayeweza kujadiliana nawe.

2. Kwa watu ambao wamo katika vikundi vyote viwili, timu ya utafiti itahifadhi takriban mililita 5 (kijiko kimoja cha chai) ya damu pale KEMRI/WRP CRC Kericho wakati vipimo vya mahabara vitakapofanywa miezi mitatu baada ya kuanzishiwa ART. Katika kipindi cha miezi sita baada ya utafiti kukamilika, mahabara itatumia damu hii kuchunguza kiwango cha virusi vya HIV kilichogandamizwa kutokana na matumizi ya ART. Hii pia itasaidia watafiti kutambua utendakazi wa ART katika kipindi cha miezi 3 ya kwanza baada ya kuanzishwa matibabu haya. Japo kipimo hiki kitakapofanywa, matokeo yatapokezwa kwa daktari wako anayeweza kujadiliana nawe.

Hakuna damu itahifadhiwa miezi 6 baada ya kukamilika kwa utafiti na baada ya kuzua habari zote muhimu kutoka kwa rekodi zako za kimatibabu.

Kwa watu wote walio katika utafiti huu, daktari wako ataweza kufanya uchunguzi kutambua iwapo kumezuka pingamizi na aina ndogo tofautitofauti za HIV tokana na matumizi ya dawa yoyote unayotumia kwa matibabu ya HIV. Pingamizi inamaanisha kuwa moja au zaidi ya madawa unayotumia katika matibabu ya HIV hayana utendakazi. Yaani, kirusi kimezua “pingamizi.” Vipimo hivi vya kunguza pingamizi na aina ndogo tofautitofauti za HIVvitafanywa iwapo daktari wako anahisi kuwa ART ulioanzishiwa haina utendakazi na anataka kubadilisha ART kwa aina nyigine ya pili. Iwapo hili litafanywa, daktari wako atawasiliana nawe kuhusu matokeo ya vipimo hivi. Pia baada ya kukamilika kwa utafiti, daktari wako atachunguza pingamizi dhidi ya ART iwapo itaonekana kuwa virusi vya HIV havijagandamizwa.

Kwa kukamilisha, timu ya utafiti itahifadhi takriban mililita 5 ya damu yako (takriban kijiko kimoja cha chai) kabla ya kuanza matibabu ya ART kuchunguza iwapo kati ya wale wanaoanzishiwa ART wanapingamizi. Kwa wakati huu, tunapochunguza na kutambua kuwa kuna pingamizi na aina ndogo tofautitofauti za HIV kabla ya kuanzishiwa ART, sio kila mtu atapata kufanyiwa. Iwapo kipimo cha pingamizi kitafanywa kwa damu yako kabla ya kuanzishiwa ART, utapata matokeo japo yanapotokea. Hata hivyo ni muhimu kutambua kuwa vipimo hivi havitafanywa kwa muda baada ya utafiti kuanzishwa.

NI WATU WANGAPI WATASHIRIKI KATIKA UTAFITI HUU?

Inafikiriwa kuwa takriban watu 820 watashiriki katika utafiti huu katika vikao 7 vya matibabu katika mikoa ya Rift Valley na Nyanza.

NITAKUWA KATIKA UTAFITI HUU KWA MUDA GANI?

Japo utakapoanzishiwa matibabu ya ART, tutazua habari kutoka kwa rekodi zako za kimatibabu kwa muda wa mwaka 1 ½ . Hii huenda ikawa miezi kadhaa zaidi kulingana na wakati daktari anakuomba urejee kwa ziara zako za kawaida.

Japo tutakapokoma kuzua habari na utafiti kukamilika, utaendelea na matibabu yako ya ART kama kawaida.

.

SABABU ZA KUONDOLEWA KATIKA UTAFITI MAPEMA

Unaweza kuondolewa katika utafiti huu mapema kwa sababu zifuatazo:

1. Kwa ombi lako kuacha kushiriki katika utafiti huu.
2. Daktari wako na mhudumu katika kliniki ya HIV kuhisi kuwa utafiti hautilii maanani maslahi yako.

Mashirika yanayochunguza utafiti huu kama vile Bodi za Ukaguzi (KEMRI na/ AU WRAIR), Wizara ya Afya ya Kenya, mfadhili wa utafiti (Ofisi ya kuelekeza mambo ya UKIMWI Duniani/PEPFAR) wakihisi ni vyema kusitisha utafiti kwa pamoja.

Kikundi kinachoitwa “Kamati ya kuchunguza Deta” itafuatilia maendeleo ya utafiti. Yaani, itachunguza habari zinazotolewa kutoka kwa rekodi zako. Kamati hii itatathmin maendeleo ya mradi kama vile, ni watu wangapi wanajisajilisha katika utafiti, ni watu wangapi wanafeli katika uchunguzi wa vipimo vya kiwango cha virusi, ni watu wangapi wanatambulika na daktari kuwa na matibabu yanayofeli; na mambo mengineyo. Kamati hii haitapokea habari zozote zilizo na majina ya washiriki. Takriban nusu ya muda wa utafiti, kamati hii itaangalia iwapo njia moja ya ufuatilizi (kikundi A au kikundi B) huenda ikawa ya manufaa zaidi kuliko kinginecho. Iwapo kimoja kitakuwa cha manufaa zaidi nusu ya kipindi cha utafiti, kamati huenda ikapendekeza utafiti kusitishwa. Iwapo hili litafanyika, utaelezewa kuhusu matokeo. Utaendelea kupokea utunzaji katika kliniki ya ART kama kawaida.

NI ZIPI ATHARI ZA KUSHIRIKI KATIKA UTAFITI HUU?

Daktari wako na wahudumu katika kliniki ya HIV watakuelezea kuhusu athari zinazohusika katika kutumia ART. Hizi athari zipo hata ujiunge au usijiunge na utafiti. . Hata hivyo, daktari wako na timu ya utafiti itafanya lolote ili kupunguza nafasi za madhara haya kukuathiri.

Huenda kuna uwezekano wa athari ya kijamii ya kutengwa kwa sababu ya HIV na UKIMWI. Kwa bahati, kikao chako cha matibabu kimekuwa kikiendelezwa kwa takriban miaka 3 na kina kliniki maalum ya HIV. Hivyo basi, athari ya kijamii kwako kutokana na HIV au UKIMWI inayohusika na kutengwa ni wa kiwango cha chini sio kama hapo awali wakati ART hazikupatikana kwa urahisi. Kama kwa wagonjwa wote katika kliniki ya ART, daktari wako na watunzaji wa kiafya watakuwa na umakinifu katika kutengwa dhidi ya HIV na UKIMWI. Iwapo kwa wakati wowote umeathirika kwa njia yoyote ile (Kijamii au kimwili), unafaa umjulishe daktari wako au mhudumu katika kliniki ya HIV au wahudumu katika timu ya utafiti ya CLADE.

Kuna uwezekano kuwa huenda ukaathirika wakati wowote damu inapotolewa ili kufanyia vipimo vya mahabara. Haya yanahusisha kuhisi udhiko, kutokwa kwa damu au kuchibuka mahali sindano inapoingizwa mwilini, kuhisi kizunguzungu na kwa nadra kuzimia au kupata ambukizo. Haya ndiyo matokeo hata kama haupo kwenya utafiti.

Kwa kukamilisha, huenda habari kutoka kwa rekodi zako za matibabu zikatambulika katika utaratibu wa kuzua deta. Huku ikisisitizwa kuwa timu haitatumia jina au anwani yako, hatuwezi kuhakikisha asilimia mia kuwa hili halitafanyika kwenye kliniki. Pia, tutasafirisha habari kutoka kwa rekodi zako za kliniki hadi kikao cha utafiti cha KEMRI/WRP karibu na Hospitali ya Wilaya ya Kericho. Kando na habari za usajili na

idhini, tutasafirisha habari hizi kwa kutumia kompyuta bila kutumia jina au anwani yako. Punde habari hizi zitakapopokelewa katika kikao chetu cha utafiti, tutafuta maelezo katika kompyuta iliyotumika katika kusafirisha habari hizo. Habari iliyochukuliwa mwanzoni iliyo na jina na vitambulishi vinginevyo iliyochukuliwa katika ziara ya kwanza itahifadhiwa katika sehemu tofauti iliyolindwa kwa kutumia neno maalum ambalo linatumika na meneja wa deta, naibu wake chini ya uelekezi wa mkuu wa utafiti.

KUNA FAIDA ZOZOTE ZA KUSHIRIKI KATIKA UTAFITI HUU?

Kuna uwezekano kusiwe na faida ya moja kwa moja kwa kuwepo katika utafiti huu. Iwapo utashiriki katika utafiti huu, huenda ukanufaika wewe mwenyewe, lakini hili sio hakikisho kamili. Katika kushiriki katika utafiti huu, huenda ukajifunza zaidi kuhusu ushauri na ufuatilizi wa hali yako ya virusi kuliko kawaida. Kwa mfano, kiwango cha virusi au vipimo vya pingamizi huenda zisifanywe kama mojawapo ya taratibu. Bali na kuwa na uhakika na huku tukijaribu kuelewa kutokana na utafiti huu, huenda kukawa na matokeo mazuri zaidi unapotumia ART huku ukifanyiwa taratibu za kufuatilia kiwango cha virusi. Kwa tamati, katika kushiriki katika utafiti huu, huenda ukapimwa kiwango cha virusi na uzuizi wa virusi ambapo itasaidia kuchuwa ni dawa gani ya ART inakufaa.

NI LIPI CHAGUO LINGINE BALI NA KUSHIRIKI KWANGU KATIKA MRADI?

Kushiriki katika utafiti huu ni kwa hiari. Unaweza kuamua kutoshiriki kwa sasa au wakati wowote baadaye iwapo utaamua kushiriki sasa. Utaendelea kupokea matibabu ya ART katika kikao hiki kama mtu mwingine yeyote.

Tafadhali zungumza na daktari wako kuhusu haya na chaguo lingine ulilo nalo.

ITAKUAJE KUHUSU ULINZI WA SIRI?Timu ya utafiti watakupa nambari maalum ya utafiti ya kujitambulisha. Nambari hii ya kujitambulisha (sio jina lako wala habari yoyote inayoweza kutumiwa katika kukutambua) itatumiwa kuzua habari kutoka kwa rekodi zako za kimatibabu. Rekodi zako zitawekwa katika chumba kilichofungwa. Ni wahudumu wa utafitii pekee watakaokuwa na funguo. Hakuna chapisho la utafiti huu litakalotumia jina lako au kukutambulisha kibinafsi.

Juhudi zitafanywa kuweka siri habari zakoza kibinafsi, hata hivyo hatuwezi kutoa hakikisho la ulinzi mkamilifu wa siri. Habari za kibinafsi huenda zikafumbuliwa iwapo itahitajika kisheria. Rekodi zako pia huenda zikakaguliwa na mfadhili wa utafiti huu, Idara ya Ulizi (DoD)/ Taasisi ya Utafiti ya Jeshi la Walter Reed (WRAIR), kitengo cha utafiti cha jeshi la merikani na makamanda(USAMRMC), ofisi inayo shughulikia utafiti wa wanadamu na ulinzi(ORP) ,afisa mkuu anaye simamia ulinzi na utafiti kwa mwanadamu(HRPO),Taassi ya utafiti wa kimatibabu ya Kenya(KEMRI) na watu wengine waliyo na mamlaka kama ilivyoorodheshwa katika sera za Kenya na Amerika.

NITAPATA MALIPO YOYOTE?

Wasimamizi wa hospitali wataamua iwapo utapata fidia kwa kushiriki katika utafiti huu kulingana na desturi ya hospitali yako. Iwapo wasimamizi wataamua kuwa washiriki katika mradi wa CLADE watapata fidia, utapata fidia kwa usafiri na muda wowote uliowekwa kwa ajili ya kushiriki katika utafiti.
Kila mwisho wa ziara za kawaida za kliniki au utafiti utapokea shilingi 400-500 za Kenya. Kwa ziara zozote zisizopangiwa, utapewa shilingi 200 za Kenya.Kliniki yaweza kuamua kuwa fedha za ushiriki zitawekwa kwa ajili ya uimarishaji katika kliniki ya HIV kwa wagonjwa wote.Iwapo itakuwa hivyo, hautapata fidia kwa kushiriki katika CLADE.

GHARAMA KWANGU NI IPI?

Utafiti huu hautakulipisha chochote iwapo utakubali kujiunga na kukubali timu ya utafiti kuzua habari kutoka kwa rekodi zako. Tafadhali elewa ya kuwa utafiti huu wa CLADE hautachukua jukumu la kutoa huduma zako za utunzaji au matibabu. Gharama ya utunzaji na matibabu yako bado ni jukumu lako au la kampuni inayokupa bima, au mfumo wa kutoa huduma za afya. Utafiti huu hautakupa pesa zozote za kununua madawa dhidi ya HIV au kugharamia jambo lisilotarajiwa katika muda unaopokea matibabu ya HIV. Hata hivyo tunatoa hakikisho kuwa hakuna gharama itakayopitishwa kwako kwa vipimo vyovyote vya utafiti au ufuatilizi wa matokeo na katika kupokeza daktari wako kwa matumizi ya matibabu yako.

Tafadhali elewa ya kuwa kikao chako cha matibabu huenda kikawa na gharama kama vile kusajili kwa wagonjwa na utunzaji wa kikao, haya malipo ni kwa kila mtu anayepokea matibabu katika kikao hiki hata wale wanaopokea ART. Hili litakuwa jukumu lako kutoa malipo haya.

NI LIPI LITAFANYIKA IWAPO NITAJERUHIWA?

Iwapo utapata majeraha yanayotokana moja kwa moja na kushiriki kwako katika utafiti huu, utapata matibabu ya dharura bila ya gharama yoyote, kwa jeraha hilo. Hakuna mpango wa kukupa fidia kwa ugonjwa au huera lolote aidha kupitia Taasisi ya Utafiti wa Kimatibabu ya Kenya, Taasisi ya Utafiti katika Jeshi la Walter Reed, au mradi wa PEPFAR. Utapata abarrí kuhusu mahali unaweza kupokea matibabu zaidi. Pia elewa kuwa hii sio njia ya kuhepa au kukupokonya haki zako za kisheria. Ni vizuri kujadiliana swala hili kwa undani na timu ya utafiti kabla ya kusajili katika utafiti.

Iwapo utajeruhiwa au kuwa mgonjwa kutokana na utafiti huu, utapokea huduma za kimatibabu katika hospitali yako unapopokea matibabu. Utapokea matibabu tu kwa majeraha yanayotokana moja kwa moja na kushiriki katika utafiti. Taasisi ya Utafiti wa Kimatibabu ya Kenya, Taasisi ya Utafiti katika Jeshi la Walter Reed, au mradi wa PEPFAR halitagharamia usafiri wako kwenda na kutoka hospitali au kwenye kliniki

Iwapo una maswali kuhusu huduma zako za kiafya, zungumza na Dkt. Fredrick Sawe,

(nambari za simu: 052-30388; 0724-255623) au Dkt Jonah Maswai(nambari ya simu 052-30388;0716- 430271) Iwapo utagharamia huduma zakoza kiafya kwingineko kwa majeraha yaliyosababishwa na kushiriki katika utafiti, wasiliana na msimamizi mkuu.

Iwapo jambo hili haliwezi kusuluhishwa, wasiliana na mkuu wa Bodi ya Ukaguzi katika Taasisi ya KEMRI kupitia nambari ya simu 020-2722541 au Taasisi ya Utafiti wa Kimatibabu na Usimamizi wa Bidhaa katika Jeshi la Marekani (USAMRMC) Afisi ya Wakili kwa nambari ya simu (301) 619-7663/2221.

NI NINI HAKI YANGU KAMA MSHIRIKI KATIKA UTAFITI?

Kushiriki katika utafiti huu ni kwa hiari. Unaweza kuchagua kutoshiriki katika utafiti huu au unaweza kuacha kushiriki wakati wowote. Utapata huduma sawa bila ya kujali uamuzi wako.

Tutakuelezea kuhusu habari mpya kutokana na utafiti huu au tafiti zingine ambayo inaweza kuathiri afya yako, ustawi, au hiari yako ya kutaka kusalia kwenye utafiti. Iwapo utahitaji matokeo ya utafiti huu, tafadhali julisha muhudumu wa utafiti.

NITAFANYA NINI IWAPO NINA MASWALI AU MATATIZO?

Kwa maswali yoyote kuhusu utafiti au majeraha yanayotokana na kushiriki kwako katika utafiti wasiliana na daktari katika utafiti/mtafiti:

Dkt Fredrick Sawe
Taasisi ya Utafiti wa Kimtabibu Ya Kenya / Kituo cha Utafiti wa Kimatibabu cha Mradi wa Walter Reed,
Hospital Road
PO Box 1357,
Kericho-20200, Kenya
Nambari ya simu: (254-52) 30388/32101
Simu ya rununu: (254) 724- 255623
Tarakilishi: (254-52) 30662 / 30546
Barua pepe: fsawe@wrp-kch.org

Kwa jambo lolote la dharura unaweza kuwasiliana na daktari wako, mhudumu katika kliniki ya HIV au daktari katika utafiti wa CLADE kwa nambari ya simu 0723-226-229 (laini hii ya dharura i wazi masaa 24)

Iwapo huwezi kuwafikia watafiti wowote kwa nambari zilizoorodheshwa hapo juu, kwa jambo lolote la dharura waweza kuwasiliana na Rither Langat mwelekezi wa utafiti *(nambari za simu: 052-30388; 0713603289).*

Kwa maswali yoyote kuhusu haki zako kama mshiriki katika utafitii huu au malalamishi

yoyote kuhusu mradi, msimamizi mkuu wa bodi ya ukaguzi wa taasisi ya KEMRI aweza kufikiwa kwa nambari ya simu 020-2722541.

UKURASA WA SAHIHI

Ikiwa umesoma hii fomu ya idhini (au umeelezewa juu yake), maswali yako yote yamejibiwa, na unakubali kushiriki katika utafiti huu, tafadhali tia sahihi jina lako hapa chini.

____

Jina la mshiriki (chapa) Sahihi ya mshiriki
Au alama ya kidole gumba na tarehe

____

Anwani ya mshiriki

____

Jina la mhudumu wa utafiti anayeendesha majadiliano ya idhini (chapa) Sahihi ya mhudumu na tarehe

____

Jina la shahidi (chapa) (kama inavyostahili) Sahihi ya shahidi na tarehe
