# Supplementary material for: Kericho CLinic-Based ART Diagnostic Evaluation (CLADE): Design, Accrual, and Baseline Characteristics of a Randomized Controlled Trial Conducted in Predominately Rural, District-Level, HIV Clinics of Kenya
Source: PLoS One. 2015 Feb 23;10(2):e0116299. doi: 10.1371/journal.pone.0116299 (PMC4338154; doi:10.1371/journal.pone.0116299)
Supplement: S3 Consent — (PDF) [file pone.0116299.s005.pdf]

THE KENYA MEDICAL RESEARCH INSTITUTE (KEMRI) / WALTER REED ARMY INSTITUTE OF RESEARCH (WRAIR) / WALTER REED PROJECT (WRP)

CLINIC-BASED ART DIAGNOSTIC EVALUATION (CLADE)

NONRO MAR DUOKO MAR NGIMA MAR GALAMORO

Otas mar 2.0, February 13, 2012

WECHE MOTELO

Ma en otas mar yierrouk mar nonro ma itimo gi Jo Kenya Medical Research Institute (KEMRI), kod Walter Reed Army Institute of Reseach (WRAIR) / Walter Reed Project (WRP). Nonro ni iluongo ni 'CLADE' mochungne "**CL**inic-based **A**RT **D**iagnostic **E**valuation" matiende ni "ng'eyo kendo ngiyo kaka yien mag ayaki tiyo kaluwore gi kaka ichiwo gi kar thieth."

Nonro ma iluongo ni CLADE ochung'ne kendo ichulo gi US Office of the Global AIDS Coordinator (O-GAC) / Presidents Emergency Plan for AIDS Relief (PEPFAR) Program. Laktare motelo ne nonroni e kar timo nonroni en Laktar Fredrick Sawe (052-30388; 0724-255-623) kod Laktar Jonah Maswai (052-30388; 0716-430-217) mar Kenya Medical Research Institute/Walter Reed Project Clinical Research Center (Hospital Road, namba mar Barua 1357, Kericho-20200, Kenya)

Ikwayi ni mondo idonjie nonroni nikech ibiroga e klinik mar joma ni gi kute mag ayaki to kendo ibiro bedo ka iyudo yien mag Ayaki kanyo. Ka pok iyiero mar donjo e nonroni dwaher mondo ibed gi ng'eyo ewi nonroni.

Otas yierruokni lero weche mag nonroni. Jatij nonro biro lerore kodi ewi wechegi. In thuolo mar penjo penjo ewi nonroni e saa a saya. Ka iyie donjo e nonroni ibiro kwayi mondo igo seyi e otas lerruokni kendo ibiro miyi otas machal kod migoye seyi mondo ikan.

Kuom yie ng'e ni:

- Bedoni e nonroni en kuom chiwruok mari.
- Inyalo weyo bedo e nonroni e saa asaya.
- Pod ibiro yudo yedheni mag kute mag Ayaki e klinik ka kaka ngata ngata machielo kata kapo ni ok idonjo e nonroni.

ANG'O MA OMIYO ITIMO NONRONI?

Gima omiyo itimo nonroni en mondo warang' ane yore ariyo ma migawo mar thieth (MOH) oseyango mar luwo jotuo ma yudo yien mag kute mag Ayaki. Yo achiel en ka Daktachi pimo dendi kendo ka orango Kwan mar CD4 (askache meg dendi) kopimo kod nge'ny mag kute mag Ayaki moyangi.Yo machielo en ka Daktachi pimo dendi, orango

kwan mar CD4 kendo otimo pim mar ng'iyo ng'eny mag kute ma iluongo ni Viral Load mapile. Yore gi duto konyo nyiso ber mar tich mag yedhegi mag kute mag Ayaki. Kwan mar CD4 irango kaka achiel kuom chenro mar thieth mapile e klinik ka bang dweche auchiel ka auchiel kata kaka Daktachi diher mondo opime. Kwan mar ng'eny mar kute osepuodhi gi migawo mar thieth to ok range kaka achiel kuom chenro mar thieth mapile e klinik ka nikech neng'o mar pim ni kendo gik pimo ok yudre eyo mayot.

Ok wang'eyo ka pimo ng'eny mar kute mag ayaki saa asaya ma ipimo Kwan mar CD4 biro konyo Daktachi ng'eyo ka yedhegi mag kute mag Ayaki tiyo eyo maber. Ma ema omiyo itimo nonroni.

ANG'O MA IDWARO NI MONDO ATIM E NONRONI?

Mondo idonj e nonroni, nyaka ibed gi higni 18 kadhi nyime. Joma chwo gi mine nyalo donjoe. Mine ma pek ok nyal donjo e nonroni, makamana ka miyo obedo mapek e ndalo mag nonro to Daktachi oyiero Ni pod idhi nyime gi yedhe mag kute mag Ayaki, pod inyalo dhi nyime gi nonro. Ka iyiero mar donjo e nonroni, nyaka ine ni ibiro e limbegi duto ma ochiki mag thieth. Ma en gima duong' moloyo kata ka ichano bedo kata wuok e nonro. Ka idonjo e nonro, ibiro dwari ni ibi kar thieth e limbegi ma ochiki mag kawo yedheni. Ma en diriyo e dwe kuom dwe mokuongo. Bang'e, to laktachi nyalo chiki ni mondo ibi dwe ka dwe kuom dweche mang'eny. Ka isebedo ka itiyo gi yedhegi kendo koro idhi maber, laktachi nyalo medo thuolo mar biro e limbe mochiki ekind dweche 3 kata 6. Ndalo magi mag biro e klinik nyalo lokore ka ituo kata kaka laktachi paro ni mondo iduogi kuom thuolo malach kata machuok. Bedoni mangima e gima duong' ma onego mi iduogi ka laktachi dwari. Duogo e limbegi mochiki en gima duong' ne nonroni nikech jotij nonro diher mar choko weche koa kuom otase gi mag limbe mondo ging'e godo kaka idhi.

Ok onego bed ni itiyo gi seche mang'eny momedore e saa a saya mar limbe ka idonjo e nonroni. Onego inyis sista mar klinik ni in e nonro sama ichopo. Katakamano sechegi mag limbe biro bedo machalre kata ka in e nonro kata ka ionge. Jotij nonro biro rango wechegi mochoki ka limbeni oserumo. Weche ma jotij nonro biro kawo e otasegi mag limbe gin mana wechegi maondiki e otesegi kende (to ok nyingi kat kuma iaye) kuom ranyisi kaka iwinjo, ratil mari, rang'iny mar somo mari, tuo moro amora ma in godo, ka ne oruaki ma inindo ei wuod, kaka imuonyo yedhegi mag kute mag Ayaki to gi yedhe mamoko mag Ayaki, dwokogi mag pim, to gi yedhe mamoko kata pim ma laktachi nyalo kwayo mondo otimni.

Jotij nonro biro ng'iyo ni weche mochok kuomi okan e yo malingling. Mondo omi watim ma , wabiro miyi namba mar nonro (SID) ma ibiro tiyogo e kinde ma wachoko wechegi koa e otesegi mag thieth. Wabiro tiyo mana gi nambani to ok nyingi ka wachoko wechegi mondiki ei otesegi mag thieth.

Ka nonro chakore , ibiro keti kor ka ng'iyo kaka yedhe mag kute mag ayaki tiyo ka itiyo gi rango dendi kod kwan mar CD4 kipimo godo nge'ny mag kute mag Ayaki moyangi(miluongo ni 'kanyakla mar A') kata ng'iyo kaka yedhe mag kute mag ayaki

tiyo ka itiyo gi rango dendi kod kwan mar CD4 kod kwan mag kute ma ilungo ni Viral Load (miluongo ni ‘kanyakle mar B’). Ka in e kanyakla mar A, ibiro pimi kendo ibiro yudo pim mar CD4 dweche auchiel ka auchiel (kata sama laktachi oneno ni owinjore) kipimo godo nge’ny mag kute mag Ayaki moyangi kaponi yien ma imwonyo ok ti eyo makare. Ka in e kanyakla mar B, ibiro pimo dendi kendo timo kwan mar CD4 kod ng’eny mar kute mag ayaki bang dweche auchiel ka auchiel (kata sama laktachi oneno ni owinjore).

In gi thuolo mar bedo e kanyakla moro a mora, kaka diro otonglo malo. In kata laktachi, gi jotij noro ok bi ng’eyo kanyakla maintiere. In kata laktache kata jotij nonro ok nyal loko kanyakla maintiere.

Yie inge ni ka in e kanyakla ma ok pim ng’eny mar kute mag ayaki (kanyakla A) to laktachi neno ni onego otimni pim mar ng’eny mar kute mag ayaki kata ka oparo ni yedheni ok ti maber, laktachi pod nyalo pimo ng’eny mar kute mag ayaki. Ma ok bi tim pile ka in e kanyakla maok pim ng’eny mar kute mag ayaki. Ka laktachi okwayo pim mar ng’eny mar kute mag ayaki, to ibiro miyi dwoko.

Nitie kido ariyo mag pim ma ibirotim kaka dwaro mar nonro kendo olergi piny kanyo.

1. ka orwaki e kanyakla ma itimonegi pim mar del kod kwan mar CD4 mondo ong’e godo kaka yath tiyo (kanyakla A), jo nonro biro kano remo maromo milimita 5 (madirom kijiko achiel mar chaye) e kar KEMRI/WRP CRC Kericho saa asaya ma laktachi okwayo pim mar CD4. Ma en kaka dichiel bang dweche auchiel. E kinde mag dweche auchiel ka nonro oserumo, jopim biro tiyo gi remogi mondo gi ne kendo gi ng’e kwan mar kute mag ayaki sama opim CD4. Ma bende biro konyo jotim nonro ng’eyo ka pimo ngeny mar kute mag ayaki sa asaya ma ipimo CD4 konyo. Ka osetim pimgi, dwokogi ibiromiyo laktachi ma biro wuoyo kodi ewigi.
2. Kuom joma nitiere kanyaklagi duto, jononro biro kano milimita 5 mar remo (madirom kijiko achiel mar chaye) e kar KEMRI/WRP CRC Kericho mibirotim bang dweche adek ka isechako tiyo gi yath. E kinde mag dweche auchiel ka nonro oserumo, jopimo biro tiyo gi rembi ka ging’iyo Kwan mag kute mag ayaki ma yath osedwoko chien. Ma bende biro konyo jononro ng’eyo kaka yadh kute mar ayaki tiyo maber e thuolo mar dweche adek mokwongo ka osechak tiyo kode. Bang ka osetim pim, dwokogi ibiromiyo laktachi ma biro wuoyo kodi ewigi.

Onge remo ma ibirokan bang dweche auchiel bang ka nonro oserumo kendo weche duto mag kinde ka kinde osechoki koa ei otesegi mag thieth.

Ne ji duto manitiere e nonroni, daktachi biro ng’iyo ka dendi otamre tiyo gi yath kod kidieny mar yath moro a mora mar thiedho kute mag ayaki. Ka dendi otamre tiyo gi yath nyiso ni yath achiel kata moloyo achiel mitiyogo kuom thiedho kute mag ayaki ok ti kuom thiedhi. Ma tiende ni kute mag ayaki koro ‘ok winj yath’. Pim mar ng’eyo ka kute otamore tiyo gi yath kod kidieny mar yath bende ibiro tim ka daktachi paro ni yien mane ichakogo otamre tiyo to odwaro ni mondo olokni yath mondo ichak tiyo gi sidieng’ mar

ariyo mar yath . Ka ma otimore to daktachi biro nyisi dwoko mar pimni. Kendo e giko mar nonro, daktachi biro ng'iyo ka dendi otamore tiyo gi yath kod kidieny mar yath, to kendo ka pim nyiso ni yadhi ok dwok chien kutegi mag ayaki.

Mogik, jotij nonro biro kano remo maromo mililita 5(madirom kijiko achiel mar chai) kapok ichako yedhegi mag kute mag ayaki mondo ong'e godo ka moko kuom yedhe ma ichakogo ok ti. Kawatimo pim ni mar ng'eyo ka dendi otamre tiyo gi yath, ji duto ok bi yudo pim ni. Ka pim mar tamruok mar dendi tiyo gi yath otim kapok ichako yath, ibiro miyi dwoko gi ka oseyudgi. Kata kamano onego ing'e ni pim ni ok bi tim kuom ndalo bang' ka nonro osechakre.

JI ADI MABIRO BEDO E NONRONI?

Iparo ni kwan mar ji madirom 820 biro donjo e nonro e kuonde 7 mag kar thieth mag kute mag ayaki e hoho mar okak (rift valley) kod nyanza.

ABIRO BEDO E NONRONI KUOM THUOLO MAROMO NADE?

Ka isechako muonyo yath, wabiro choko wechegi mowuok e otesegi mag thieth kuom thuolo maromo higa achiel gi nus. Ma nyalo bedo kuom dweche mathoth kaluwore gi thuolo ma daktachi ochiki ni mondo iduogie limbe mochiki. Jo KEMRI kod WRAIR biro kawo wechegi mochoki e nonroni.

Bang' ka waseweyo kawo wechegi mochoki kendo nonro oserumo, ibiro dhi nyime e thuolo ma pile gi thieth mar geng'o tuo mar ayaki.

GIMA OMIYO INYALO GOLI E NONRO KAPOK NONRO ORUMO.

Inyalo golie e nonroni kapok nonro orumo kaluwore gi weche manitiere piny kae:

1. Ka ikwayo ni mondo ogoli e nonro.
2. Ka daktachi marangi kata jatij kar thieth paro ni nonroni koro ok ber kodi.

Riwruoge mang'iyo nonroni kaka Institutional Review Boards (KEMRI kod WRAIR), migawo mar thieth mar Kenya (MOH), kata jachul nonro (Office of the Global AIDS Coordinator/PEPFAR), dwaro ni mondo nonro ochungi.

Kanyakla miluongo ni "Data Monitoring Committee" biro ng'iyo kaka nonro dhi. Gi biro ng'iyo wechegi michoko koa e otesegi mag thieth. Jobura gi biro ng'iyo kaka nonro dhi ka ging'iyo weche kaka; ji adi madonjo e nonro; ji adi ma okbi kalo pim mar ng'eny mar kute mag ayaki; ji adi ma daktache gi nyalo neno ni ok dhi maber gi thieth; kod gik mamoko. Jobura gi ok bi yudo wach moroamora manigi nying jachiwre. Ka nonroni ochopo e diere jobura biro ng'iyo ka achiel kuom yore mag rango (kanyakla mar A kata kanyakla mar B), nenore ni dhi maber moloyo nyawadgi. Ka oyudore ni achiel ber moloyo ka nonro osegik e diere, to joburagi nyalo puodho ni mondo nonro ochungi. Ka ma otimore, to ibiro nyisi duoko. Ibiro dhi nyime gi yudo yedhe ma geng'o tuo mar ayaki kaka pile e klinik.

EN RACH MAGE MANTIERE E NONRONI?

Daktachi kod jotij klinik biro nyisi rach ma inyalo bedo godo ka itiyo gi yedhe mag gengo kute mag ayaki. Rachgi nyalo bedo kata idonjo kata ok idonjo e nonroni. Rach mamoko nyalo betie kuom bedo e nonroni. Kata kamano, daktachi kata jotij nonro biro temo matek mondo giduok piny thuolo mari mar bedo gi rach. Inyalo bedo gi rach e wi tudruok gi ji kaka akwede majabedoga nikech kute mag ayaki kata ayaki. Kuom hawi maber, kari mar thieth osebedo katiyo kuom higni 3 kendo koro ji ongeye kaka kar thieth mar kute mag ayaki. Koro, chandruok mar tudruok gi ji mabiro nikech kute mag ayaki kata ayaki koro osedok chien kipimo gi ndalo mane yath mar thieth ne pod ok yudre eyo mayot. Kaka jotuo duto manitiere e klinik mar yath, daktache kod jomathietho biro bedo motang gi chandruok mar tudruok mabiro nikech kute mag ayaki kata ayaki. Ka iwinjo ni in gi hinyruok e saa asaya eyo moro amora (kor ka tudruok kata e dendi), onego inyis daktachi kata jatij klinik kata jatij nonro mar <u>CLADE</u> mondo ong'e.

Nitie thuolo ni inyalo bedo gi hinyruok e saa asaya ma rembi okaw mondo odhi opim e kar pim. Ma en kaka rem, chwer, kata ridhruok kama ochwo sindan, kawirawira, kod matin ahinya rumo gi muya kata yudo touché. Ma nyalo timore kata ok in e nonro.

Mogik, nitie thuolo ni weche mochoki koa e otesegi mag thieth nyalo nenore e sama ichokogie. Kata ka jotij nonro ok bi tiyo gi nyingi kata adresi, ok wanyal bedo ga diera ni ma ok nyal timore e klinik. Kendo wabiro tero wechegi mochoki koa e otesegi mag thieth e kar nonro mar KEMRI/WRP ma okiewogi Kericho District Hospital. Kopogre gi weche mag Kwan ji kod mag yieruok modong' wabiro oro ei komputa maonge nyingi kata adresi. Ka wechegi osechopo e kar nonro, wabiro rucho gi koa kuom komputa mane otigo kuom kowogigo. Weche mokuongo maneochoki manyiso nyingi kod yo moro amora mainyalo ng'eyigo ibiro kan e kanyakla mag weche kuma opogre ma orit gi ndiko kendo ji matin e ma nyalo nenogi kaka jatend weche, jalupne kod ng'ama oyiene gi jatend nonro.

BENDE NITIERE BER KUOM BEDO E NONRONI?

Nyalore ni ok inyal yudo ber moro amora kuom bedo e nonroni. Ka idonjo e nonroni inyalo bedo gi ber moko to kata kamano onge adiera. Kuom bedo e nonroni, inyalo bedo gi ng'eyo malach e yore mag rango kaka kute gi mag ayaki ing'iyo mopogre gi kaka pile; Kuom ranyisi, ng'eny mag kute kata pim mar tamruok del tiyo gi yath ma ok tim ga pile. kaka pod ok wang'eyo kendo watemo ng'eyo kuom timo nonroni nitie thuolo ni inyalo bedo gi ber kuom tiyo gi yedhegi ka timo ni pim mar ng'iyo kwan mar kute mag ayaki. Mogik kuom bedo e nonroni, inyalo bedo gi pim mar ng'eyo kwan mar kute mag ayaki kod pim mar ng'eyo ka del otamre tiyo gi yath, manyalo konyo kuom ng'eyo yo maber mar yiero yien mabeyo monego itigo.

GIN YIERO MAGE MA ANGO MA OPOGORE GI NONRONI?

Bedo e nonroni en kuom yiero mari. Inyalo yiero chiwri sani kata bang'e ka ihero chiwri. Pod ibiro yudo yedheni e kar thieth ma ka kaka ng'at ang'ata machielo. Yie iwuo kod daktachi e kuom yiero ma intiere godo.

TO BENDE NITIERE YORE MALING'LING'?

Jotij nonro biro miyi namba mar nonro ma ing'eyigo. Nambani (ma ok en nyingi kata yo moro amora ming'eyigo) ibiro tiyogo kuom choko weche moa e otesegi mag thieth. Otesegi mag thieth ibiro kano kuma ogo kiful. Jotij nonro kende ema nigi kifungune. Otas moro amora mondiki mar nonro ok bi tiyo gi nyingi kata yo ming'eyigo. Ibiro tem matek mondo okan wechegi eyo maling'ling', to kata kamano ok wanyal bedo gi adiera. Wechegi inyalo gol oko kachik mar piny dwaro ni mondo otim kamano. Wechegi bende ibiro rangi gi jachul nonro, migawo mar lweny (DoD)/Walter Reed Army Institute of Research (WRAIR) kata United States Army Medical Research and Material Command (USAMRMC), Office of Research Protection (ORP), Human Research Protections Officer (HRPO), Kenya Medical Research Institute (KEMRI), kata jok ma oyienegi gi chike mag jo Kenya kata Amerka.

BENDE ABIRO YUDO CHUDO MORO AMORA?

Jatelo mar osiptandu biro chano kapo ni inyanlo yudo chudo kuom bedo e nonroni kaluure gi chenro mar osiptandu. Ka jatelo ochano ni jogo man e nonro mar CLADE biro yudo chudo, ibiro yudo chudo mar wuoth kod kinde ma ikawo ka ibiro e nonro. E giko mar limbe mar nonro mar kinde kakinde ibiro yudo siling Ksh 500. E limbe ma ok ochiki ibiro yudo siling Ksh 200. Klinik nyalo yie mar kano omwom ma onego ochiw ni joma ochiwore ne nonro mondo omed loso go ber mar klinik mar jo HIV ni jotuo duto. Ka mano otimre, to okibi yudo chudo kuom bedo e nonro mar CLADE.

BENDE NITIE GIMA ABIRO CHULO?

Nonroni ok bi bedo gi chudo moro amora kuomi ka iyie donje kendo imiyo jo nonro thuolo mar choko weche moa e otesegi mag thieth. Onego ing'e ni nonro mar CLADE ok bi kawo ting mari mar rit kod thieth. Nengo mar rit kod thieth biro bedo mari kata mar kambi moriti, kata mar kuma iyudoe thieth. Nonro ok bi miyi pesa mar nyiewo yien makedo gi kute mag ayaki kata mar chulo weche manyalo biro e ndalogi mag thieth. Kata kamano, dwaher mar nyisi ni onge chudo moro amora ma ibiro ketoni kuom timo pim mag nonro momedore kata manyo duoko kod chiwogi ne daktachi mondo otigo kuom thiedhi.

Yie ing'e ni kari mar thieth nyalo bedo gi chudo moko kaka mar ndiko nying kod pesa mar rito osiptal, magi gin chudo ma ichulo gi ji duto mamanyo thieth kanyo koriwo nyaka jok ma imiyo yedhe mag kute mag ayaki. En ting'ni mondo ichul chudo makamago.

ANG'O MABIRO TIMORE KA AYUDO HINYRUOK?

Ka ihinyori nikech bedo e nonro, ibiro miyi thieth ma piyo ma ikonyogodo ngimani ma

onge chudo kuom hinyruokno kende. Onge chenro mar chudo ne tuo moro amora kata hinyruok ka okalo kuom Kenya Medical Research Institute, jo Walter Reed Army Institute of Research kata chenro mar PEPFAR. Ibiro nyisi kaka inyalo yudo thieth ma omedore kapo ni pod in gi dwaro mar thieth momedore. Onego ing'e ni ma ok en wito kata mayi adiera mari. Onego iwuoye wachni eyo matut gi jo tij nonro kapok idonjo e nonro.

Kapo ni ihinyori kata ituo nikech nonroni, inyalo manyo thieth e osiptandi ma pile. Ibiro thiedhi mana nikech hinyruok ma okel gi nonroni. Jo Kenya Medical Research Institute, Walter Reed Army Institute of Research, kata chenro mar PEPFAR ok bi chulo pesa ma itiyogo e wuoth ka ibiro kata ia e klinik kata osiptal.

Ka intie gi penjo ewi rit mar thiethni, wuogi laktar Fredrick Sawe (namba mar simu 052-30388; *0724-255 623*) kata laktar Jonah Maswai (namba mar simu 052-30388*; 0716-430 271*). Ka igolo pesa e mifuki ma ichulo ne rit mar thieth kamoro machielo nikech hinyruok mokelo gi nonroni to tudri gi laktar maduong' mar nonro. Ka wachno ok nyal losi, to tudri kod jatelo mar KEMRI IRB e simu 020-2722541 kata U.S. Army Medical Research and Material Command (USAMRMC), Office of the Staff Judge Advocate (ofis mar chik) e namba (301) 619-7663/2221

ADIERA MARA KAKA JACHIWRE E NONRO EN ANG'O?

Bedo e nonroni en kuom chiwruok mari. Inyalo yiero mondo kik idonj e nonroni kata weyo nonroni e sa asaya. Ibiro riti kaka pile ma ok odewo yiero mari. Wabiro nyisi weche moa e nonroni kata e nonro machielo manyalo kelo lokruok e ngimani, aritani kata dwaro mari mar dhi nyime gi nonro. Ka idwaro duoko mar nonroni to yie inyis jo tij nonro.

TO KA AN GI PENJO KATA CHANDRUOK?

Kuom penjo moro amora ewi nonro kata hinyruok mabiro nikech bedo ni e nonro, tudri gi daktach/ jatim nonro:

Laktar Fredrick Sawe
Kenya Medical Reseaech Institute/ Walter Reed Project Clinical Research Center,
Hospital Road
PO Box 1357,
Kericho-20200, Kenya
Simo: (254-52)30388/32101
Simo mar lwedo: (+254) 724-255 623
Fax: (254-52) 30662/30456
Email: fsawe @wrp-kch.org

Ka nitie gimoro ma apoya to tudri gi daktachi, jatij klinik, kata laktar mar nonro mar CLADE e namba 0723-226-229 (nambani tiyo seche 24). Ka ok inyal tudori gi jononro e namba mag simu ma ochiwgo, to nitie gimoro ma apoya inyalo tudori gi Rither Langat majatud weche mag nonro (namba simu 052-30388; 0713 603 289).

Kuom penjo moro amora ewi adiera mari kaka jachiwre e nonro kata wach ma in go ewi nonro, tudri gi jatelo mar KEMRI IRB e namba 020-2722541.

OTAS MAR SEYI.

Ka isesomo otas mar lerruokni (kata oselerni godo), penjoni duto oseduoki moromi, kendo iyie bedo e nonroni, yie igo seyi piny ka.
Otas machal kama ibiro miyi.

____________________	____________________
Nying' Jachiwre(mondiki)	Seyi jachiwre kod tarik

____

Adres mar Jachiwre

____________________	____________________
Nying' Jatij nonro malero wechegi(mondiki)	Seyi jatij nonro kod tarik
____________________	____________________
Nying janeno (mondiki)<br>(Kaka owinjore)	Seyi janeno kod tarik
