# Supplementary material for: Kericho CLinic-Based ART Diagnostic Evaluation (CLADE): Design, Accrual, and Baseline Characteristics of a Randomized Controlled Trial Conducted in Predominately Rural, District-Level, HIV Clinics of Kenya
Source: PLoS One. 2015 Feb 23;10(2):e0116299. doi: 10.1371/journal.pone.0116299 (PMC4338154; doi:10.1371/journal.pone.0116299)
Supplement: S1 Protocol — (PDF) [file pone.0116299.s006.pdf]

CLinic-based ART Diagnostic Evaluation (CLADE)

A Public Health Evaluation
Sponsored by

The President's Emergency Plan for AIDS Relief (PEPFAR),
Office of the Global AIDS Coordinator

Conducted by

The Kenya Medical Research Institute/Walter Reed Project,
Kenya Ministry of Health,
United States Army Medical Research Unit-Kenya,
and the
Division of Retrovirology, Walter Reed Army Institute of Research

Version 2.5
November 30, 2012

O-GAC #KE-07-0044

KEMRI SSC# 1717

WRAIR# 1591

RV#257

HRPO #A-14328

TABLE OF CONTENTS

I. INVESTIGATORS AND INSTITUTIONAL AFFILIATION 5

II. STUDY CONSULTANTS 6

III. STUDY MANAGEMENT 7

IV. ABBREVIATIONS 9

V. ABSTRACT/SCHEMA 11

VI. HYPOTHESES 13

1. Primary Hypotheses 13
2. Secondary Hypotheses 13

VII. OBJECTIVES 14

1. Primary Objective 14
2. Secondary Objectives 14

VIII. INTRODUCTION AND BACKGROUND 16

1. Background and Significance 16
2. HIV/AIDS and Care and Treatment in Kenya and Southern Rift Valley Province. 22
3. Study Area 24
4. Collaborating Institutions 25

IX. EVOLVING TREATMENT AND MONITORING GUIDELINES 26

X. DESIGN 29

XI. STUDY SITES AND ALLOCATED ENROLLMENT 31

XII. ANTIRETROVIRAL THERAPY AT STUDY SITES 32

XIII. CLINICAL CARE PROVIDED AT STUDY SITES 33

1. Pre-ART (Pre-Randomization) Care 34
   - A. Diagnosis of HIV infection 34
   - B. Patient evaluation and preparation for ART 34
   - C. Patient education and psychosocial assessment & preparation for ART 36
2. ART (On-Study) Care 36
   - A. Clinical monitoring 36
   - B. Laboratory testing 37
   - C. Adherence assessment and strengthening 37
   - D. Psychosocial assessment 37
   - E. Medication prescribing and dispensing 37
3. Treatment Failure 38
   - A. Routine Care (Arm A) Treatment Failure Assessment and Management 39
   - B. Viral Load Guided Care (Arm B) Treatment Failure Assessment and Targeted Viral Load (Arm A) Algorithm and Management 41
4. Additional Clinical Care 42

XIV. LABORATORY EVALUATIONS PROVIDED AT STUDY SITES AND ADDITONAL STUDY EVALUATIONS 42

1. HIV pre-ART and Routine Follow-up Laboratory Evaluations 42
2. CD4 Testing 43
3. Viral Load Monitoring 44
4. HIV Genotypic Resistance Testing 45

XV. CRITERIA FOR STUDY DISCONTINUATION 47

XVI. STATISTICAL CONSIDERATIONS 48

1. General Design 48
2. Inclusion and Exclusion Criteria 48
   A. Inclusion Criteria 48
   B. Exclusion Criteria 48
3. Endpoints 49
   A. Primary Endpoint 49
   B. Secondary Endpoints 49
4. Randomization and Stratification 50
5. Sample Size 50
6. Accrual and Replacement of Study Participants 56
7. Analyses 57
   A. Primary and Secondary Endpoints 57
   B. Cost Effectiveness Analyses 62

XVII. DATA MANAGEMENT 65

1. Data Storage and Collection at Study Sites 65

XVIII. DATA MONITORING COMMITTEE 70

XIX. ADVERSE EVENT MONITORING 71

XX. HUMAN SUBJECTS PROTECTION & ADDITIONAL ETHICAL CONSIDERATIONS 72

1. Institutional Review Board/Institutional Research Ethics Committees 72
2. Informed Consent 73
3. Recruitment 73
4. Potential Study Risks and Risk Management 73
5. Potential Study Benefits 74
6. Subject Confidentiality 75
7. Study Withdrawal 76
8. Expense to Study Participants and Compensation for Participation 76
9. Participant Remuneration 76
10. Management of Vulnerable Volunteers 76
11. Modifications of the Protocol 77
12. Protocol Deviations 78
13. Study Monitoring 78
14. Reporting of Expedited/Serious Adverse Events and Other Reporting Requirements 79

A. U.S. Army Medical Research and Materiel Command's Human Subjects Research Review Board: 79
B. Walter Reed Army Institute of Research Division of Human Subjects Protection: 79
C. Kenya Medical Research Institute: 79
15. Research Findings 81
16. Role of US Military HIV Research Program 82
17. Role of Consultants 82
XXI. RESPONSIBILITIES OF THE PROTOCOL PRINCIPAL INVESTIGATORS .. 83
XXII. REFERENCES 84
XXIV. APPENDICES…………..(Attached)
1. Appendix I. CLADE Schema
2. Appendix II. Study Sites and Allocated Enrollment
3. Appendix III. Pre-Study HIV Clinic Patient Flow and Study Enrollment
4. Appendix IV. Routine HIV/ART Clinic Flow and Study Encounters
5. Appendix V. CLADE Viral Load/Treatment Failure Algorithm
6. Appendix VI. PEPFAR PHE Technical Team Resistance Testing Sample Size Calculations
7. Appendix VII. Costing Information

I. INVESTIGATORS AND INSTITUTIONAL AFFILIATION

Kenya Medical Research Institute/Walter Reed Project HIV Program
Kericho, Kenya

1. Prof. Samuel Sinei, MBChB, MMED
2. Frederick Sawe, MBChB, MMED (Protocol Co-Principal Investigator)
3. Jonah Maswai MBChB (Lead Associate Investigator, Clinical Outcomes)
4. Milton Omondi, MBChB
5. Margaret Bii, BS (Lead Associate Investigator, Cost Effectiveness)
6. Rither Chepkemoi, RN
7. Jemutai Tarus, BPharm (Pharmacy Coordinator)
8. Raphael Lang'at, H.Dip, MLS (Laboratory Coordinator)
9. Ignatius Kiptoo, BSc, MS (IT Coordinator)
10. Peter Yegon, BSc, MSc (Data & Database Management)
11. Khamadi Samoel Ashimosi PhD
12. Kibet Shikuku, MBChB, MMED
13. Apolonia Aoko (PEPFAR Clinical care Manager)
14. Fredrick Kirui, MBChB, MMED
15. Noah Tarus, BPharm, Msc

Kenya Ministry of Health
Kericho, Kenya

1. Eunice Obiero, MBChB, MMED (Lead Associate Investigator, Clinical Care)

United States Army Medical Research Unit-Kenya
Kericho, Kenya

1. Douglas N. Shaffer, MD, MHS (Protocol Co-Principal Investigator)

Division of Retrovirology/Walter Reed Army Institute of Research
Rockville, MD, USA

1. Nelson Michael, MD, PhD
2. Mary Marovich, MD, MTMDH
3. Merlin Robb, MD
4. Tiffany Hamm, PhD

EmpiriStat
Mt Airy, MD, USA
Nicole Close, PhD (Study Statistician)

II. STUDY CONSULTANTS

Boston University School of Public Health Center for International Development
Boston, MA, USA

1. Sydney Rosen, PhD
2. Bruce Larson, PhD

III. STUDY MANAGEMENT

The study will be managed by the Kenya Medical Research Institute/Walter Reed Project HIV Clinical Research Center in collaboration with the Kenya Ministry of Health and United States Military HIV Research Program Clinical Operations Office. Key contact information for each are listed below:

<u>Kenya Medical Research Institute/Walter Reed Project HIV Clinical Research Center</u>

1. Douglas N. Shaffer, MD, MHS, FACP
   Director, United States Army Medical Research Unit-Kenya/Walter Reed Project HIV Program
   Kericho, Kenya
   Office: (254-52) 30686, 30388
   Fax: (254-52) 30546
   Cell: (254) 724-255620
   Mail: Director, Walter Reed Project HIV Program
   USAMRU-K, Unit 64109
   APO AE 09831-4109
   Email: DShaffer@wrp-kch.org

2. Frederick K. Sawe, MBChB, MMED
   Deputy Director for HIV Research and Treatment
   Kenya Medical Research Institute/Walter Reed Project HIV Program
   Office: (254-52) 30686, 30388
   Fax: (254-52) 30546
   Cell: (254) 724-255-623
   Mail: Walter Reed Project Clinical Research Center
   Hospital Road
   PO Box 1357-20200
   Kericho, Kenya
   Email: FSawe@wrp-kch.org

3. Samuel Sinei, MBChB, MMED
   Deputy Director for Program Management and Operations
   Kenya Medical Research Institute/Walter Reed Project HIV Program
   Office: (254-52) 30686, 30388
   Fax: (254-52) 30546
   Cell: (254) 727-531-396
   Mail: Walter Reed Project Clinical Research Center
   Hospital Road
   PO Box 1357-20200
   Kericho, Kenya
   Email: SSinei@wrp-kch.org

4. Mary Lelgo, BSN
Department Chief, Department of Research Support
Kenya Medical Research Institute/Walter Reed Project HIV Program
Office: (254-52) 30686, 30388
Fax: (254-52) 30546
Cell: (254) 722-941-525
Mail: Walter Reed Project Clinical Research Center
Hospital Road
PO Box 1357-20200
Kericho, Kenya
Email: MLelgo@wrp-kch.org

Kenya Ministry of Health

1. Bernard Osore, MBChB, MMED
Director of Medical Services
The Provincial Medical Office
Rift Valley Province
Office: (254 51) 2216710
Cell: (254) 721-760400
Fax: (254 51) 2210350
Mail: The Provincial Medical Officer, Rift Valley Province
Office of the Provincial Commissioner/Rift Valley Provincial Headquarters
Block B, 2nd Floor
Nakuru 20100, Kenya
Email: pmorvp@yahoo.com

United States Military HIV Research Program

1. Deline Glover, Clinical Trials Specialist
Clinical Operations Office
Office: (301) 251-5043
Fax: (301) 762-7460
Mail: U.S. Military HIV Research Program
Henry M Jackson Foundation
1600 E. Gude Drive
Rockville, MD 20850
Email: dglover@hivresearch.org

IV. ABBREVIATIONS

3TC	Lamivudine
ABC	Abacavir
AE	Adverse Event
AFB	Acid Fasting Bacilli
AIC	African Inland Church
ALT	Alanine Aminotransferase
ART	Antiretroviral Therapy
AZT	Zidovudine
BU	Boston University
CBC	Complete Blood Count
CDC	Centers for Disease Control and Prevention
CEA	Cost-Effectiveness Analysis
CLADE	Clinic-based ART Diagnostic Evaluation
CRC	Clinical Research Center
CRF	Case Report Form
CXR	Chest X-Ray
DMC	Data Monitoring Committee
d4T	Stavudine
ddI	Didanosine
DoD	Department of Defense
EFV	Efavirenz
GCP	Good Clinical Practice
HAART	Highly Active Antiretroviral Therapy
HIV	Human Immunodeficiency virus
HIVDR	HIV Drug Resistance
ICF	Informed Consent Form
IT	Information Technology
IRB	Institutional Review Board

KDH	Kericho District Hospital
KDHS	Kenya Demographic and Health Survey
KEMRI	Kenya Medical Research Institute
LFT	Liver Function Test
MOU	Memorandum of Understanding
NRTI	Nucleoside Reverse Transcriptase Inhibitor
NNRTI	Non- Nucleoside Reverse Transcriptase Inhibitor
NVP	Nevirapine
PARTO	Provincial ART Officer
PMO	Provincial Medical Officer
PMTCT	Prevention of Mother to Child Transmission
PHE	Public Health Evaluation
SID	Study Identification Number
SOPs	Standard Operating Procedures
TDF	Tenofovir
USAID	United States Agency for International Development
USAMRMC	United States Army Medical Research and Materiel Command
USAMRU-K	United States Army Medical Unit-Kenya
USMHRP	United States Military HIV Research Program
VCT	Voluntary Testing and Counseling
VDRL	Venereal Disease Research Laboratory
VL	Viral Load
WHO	World Health Organization
WRAIR	Walter Reed Army Institute of Research
WRP	Walter Reed Project

V. ABSTRACT/SCHEMA

BACKGROUND — The Kenya Ministry of Health (MoH) guidelines for antiretroviral therapy (ART) and manual for ART providers recommend targeted viral load (VL) monitoring in ART management. While only limited use of VL monitoring exist due to limitations in technical and financial resources, the superiority, feasibility, and cost-effectiveness of VL monitoring has not been prospectively studied in ART roll-out at the clinic level.

DESIGN — "**CL**inic-based **A**RT **D**iagnostic **E**valuation" (CLADE) is an unblinded, randomized (1:1), prospective, observational, cohort public health evaluation (PHE) aimed at evaluating the superiority and cost-effectiveness of two recommended MoH ART diagnostic evaluation approaches at the clinic level in adult treatment naive patients beginning MoH approved first-line ART: "routine care," the most common approach to ART roll-out where clinical (World Health Organization (WHO)) staging and immunological (CD4) monitoring are the primary baseline and follow-up evaluations and targeted viral load monitoring; and, "viral load guided care", where routine VLs are included with clinical and immunological evaluations.

DURATION — The study is expected to take 2 ½ years. It is anticipated full enrollment will take approximately 6 months to 1 year. Each participant will be followed for 18 months (1 ½ years) from time of initiating ART. Data analyses are anticipated to continue for 2 years after the last participant has completed the final study visit.

SAMPLE SIZE — 820 adult patients starting ART; 410 in each of the 2 PHE arms.

Routine Care/Arm A (n=410): will receive MoH standard of care monitoring consisting of baseline CD4 and WHO staging every 6 months, or as clinically indicated, with CD4 and WHO staging criteria guiding care and treatment in addition to routine clinical evaluations. In addition, MoH criteria for targeted viral load monitoring will be used.

Viral Load Guided Care/Arm B (n=410): patients will receive MoH standard of care as in Routine Care/Arm A but also have routine VL monitoring at baseline and every 6 months, or as clinically indicated, to guide care and treatment.

<u>REGIMEN</u> Each arm will receive Kenya MoH first-line ART. Participants meeting MoH criteria for treatment failure will begin second line ART.

<u>STUDY SITES</u> AIC Litein Mission Hospital
Kapkatet District Hospital
Kapsabet District Hospital
Kericho District Hospital
Kisumu West District Hospital
Nandi Hills District Hospital
Tenwek Mission Hospital

VI. HYPOTHESES

1. Primary Hypotheses

1. Adult patients enrolling in ART clinics who are followed by routine VL monitoring in addition routine CD4 and clinical care monitoring (VL guided care/Arm B) will have significantly less viral failures after 18 months of ART monitoring compared to those patients who are followed by routine CD4 and clinical care monitoring (routine care/Arm A) with targeted viral load monitoring.
2. Routine VL monitoring will be more cost-effective relative to routine care without VL monitoring when comparing actual incurred costs.

2. Secondary Hypotheses

1. Routine VL monitoring can be incorporated in district-level ART clinics in rural Kenya.
2. Patients followed by VL guided care compared to routine care will have less AIDS progression as defined by 2 new WHO Stage III events or 1 new WHO Stage IV event.
3. Patients followed by VL guided care will be classified as treatment failure less often than patients followed by routine care.
4. Baseline sociodemographic factors prior to ART initiation exist that are predictive of viral failures.
5. Even after adjusting for baseline sociodemographic factors prior to ART initiation, baseline HIV genotypic resistance mutations exist that are predictive of viral failure.
6. An 80% 18-month follow-up rate can be accomplished in patients initiating ART.
7. A multi-center prospective cohort study following good clinical practices (GCP) can be conducted in a non-research setting as is the case in common district-level ART clinics in rural Kenya.
8. At least 10% of participants with viral failure using a cut-off value of > 400 copies/ml would be missed if a cut off value of >1,000 were used.

9. WHO HIVDR Early Warning Indicators (EWIs) will occur in less than 10% of study participants at 6, 12, and 18 months after initiating first-line ART.
10. Baseline sociodemographic, medical, and laboratory parameters will be identified that are predictive of primary and secondary endpoint outcomes (e.g. lost to follow-up, treatment failure, death).
11. Clinical, immunological, and virological outcomes differ between participants beginning ART under previous guidelines (i.e. d4T or AZT + 3TC + NVP or EFV) compared to ART under more recent guidelines (i.e. TDF or AZT + 3TC + NVP or EFV).

VII. OBJECTIVES

1. PRIMARY OBJECTIVE

1. To compare proportions of viral failures at 18 months of follow-up among adult patients initiating ART who are followed by either 1) routine CD4 and clinical care monitoring with targeted viral load monitoring (Routine care/Arm A); or 2) routine VL, CD4 and clinical care monitoring (VL guided care/Arm B).
2. To evaluate the cost-effectiveness of routine VL monitoring in addition to CD4 and clinical monitoring in clinic-based ART management by measuring clinical and laboratory actual health outcome costs.

2. SECONDARY OBJECTIVES

1. As part of routine program implementation under the PEPFAR program, to introduce VL monitoring in district level ART clinics in rural Kenya and evaluate the feasibility of doing so by KAP (knowledge, attitude, and practice) survey of ART clinicians as well as patient/clinical care parameters (e.g. VLs being ordered correctly; VL results being returned to patients; MOH algorithm for use of VL results in evaluating treatment failure).

2. To compare proportions of AIDS progression between patients followed by VL guided care compared to routine care using a combined clinical outcome (as defined by 2 new WHO Stage III events or 1 new WHO Stage IV event).
3. To retrospectively describe the degree of agreement between persons considered not failing ART in routine guided care (Arm A) to those same individuals by conducting retrospective VLs on stored samples from persons enrolled in Arm-A on samples when a CD4 count was obtained.
4. To describe the relationship between VL and subsequent HIV resistance testing in persons evaluated for treatment failure.
5. To describe population-based HIV genotypic resistance patterns and sub-type diversity in a random sample of 200 prior to initiation of ART and on all viral failures at the final study visit.
6. To describe HIV genotypic resistance patterns and sub-type diversity in patients failing first-line ART.
7. To describe adherence to GCP as experienced in the conduct of CLADE at district-level ART clinics in rural Kenya.
8. To determine the average clinic and per-patient costs of VL ART monitoring in addition to routine clinical and CD4-based monitoring.
9. To evaluate retrospectively the presence of viral suppression (defined as VL $\leq$ 1000 copies/ml or a 1-log decrease from baseline) in persons starting ART after 3 months.
10. To quantify proportions viral failures using differing HIV-1/RNA cut-offs including > 400 copies/ml, >1,000 copies/ml, and >5,000 copies/ml and compare relative costs of each.
11. To evaluate 6, 12 and 18 month post-ART WHO HIVDR Early Warning Indicators (EWIs).
12. To evaluate baseline sociodemographic, clinical, and laboratory parameters with regard to their relationship to endpoint outcomes (e.g. lost to follow-up, treatment failure, death).
13. To compare clinical, immunological, and virological outcomes between participants beginning ART under previous guidelines (i.e. d4T or AZT + 3TC + NVP or EFV)

and those starting ART under more recent guidelines (i.e. TDF or AZT + 3TC + NVP or EFV).

VIII. INTRODUCTION AND BACKGROUND

1. BACKGROUND AND SIGNIFICANCE

With a goal of supporting 270,000 Kenyans starting ART by the end of the first 5-year funding cycle of the President's Emergency Plan for AIDS Relief (PEPFAR), approximately 180,000 Kenyans had started ART by the end of December 2007 (1, 2, 3). Beginning largely in 2004, this ambitious roll-out of ART in Kenya is supported largely by the Government of Kenya (GoK)/Ministry of Health (MoH), PEPFAR, the Global Fund to fight AIDS, Tuberculosis, and Malaria (GFATM) and other donors such as the Clinton HIV/AIDS Initiative (CHAI) and Médecins Sans Frontières (MSF) (1, 4, 5, 6, 7). ART facilities have now been opened in nearly all of Kenya's 147 districts. Given the advanced degree of ART roll-out in Kenya, attention is being given to issues around pharmacotherapy including ART education and adherence, toxicity diagnosis and management, and diagnoses and management of ART treatment failure.

While Kenya's MoH guidelines recommend viral load (VL) monitoring where available in ART management, such monitoring is done only on a small scale and sporadically throughout Kenya given limited technical capacity and resource availability (8, 9). Attention is warranted to more directly consider the feasibility and utility of VL monitoring in the setting of ART clinics given the degree of advanced ART roll out in Kenya including large treatment programs with patients moving to third line therapy (10). Such attention to VL monitoring at this point in ART roll out in sub-Saharan Africa is also supported by recent guidance and caution presented in the *Journal of Clinical Infectious Diseases* (11). While VL monitoring is now standard of care in the United States (US), extrapolation of such standards to settings throughout sub-Saharan Africa without proper costs, feasibility, and superiority evaluation and assessment may not be appropriate (12).

To date, limited, if any, prospective data are available directly relating to the utility of VL monitoring in the context of clinic-based ART roll-out as ongoing throughout sub-Saharan Africa with PEPFAR support. Four programs/studies in part sponsored by PEPFAR have offered some insight in to the utility of VL monitoring (13, 14, 15, 16). Three presentations were made at the 2007 PEPFAR Implementer's Meeting. Dr. Shumbusho presented results from a small (n=75), retrospective study evaluating agreement between clinical and immunological criteria for assessing treatment failure and change to second line therapy. The research team found only 5 of 75 patients retrospectively had viral failure and concluded very few would have benefited from regimen change (13). From a larger retrospective study of 1657 patients with suspected immunological failure, Dr. Magembe presented results where repeat CD4 counts were conducted to ascertain agreement and, on a sub-sample where VL measurements existed, true failures by VL. The team found 60% of suspected cases with repeat CD4 had confirmed immunological failure, of which, only 18% were found to be true failures. Dr. Magembe and colleagues concluded VL assays enhance accuracy of defining ARV first line failure (14).

While both of these studies suggest supportive evidence overall for the role of VL monitoring (already incorporated in to Kenya MOH guidelines), they are limited in their retrospective approach, small sample (13), and are not designed to prospectively test the questions of utility including cost-effectiveness, acceptability, and superiority of VL monitoring in the context of actual ART provision more broadly at the clinic level in differing settings. Furthermore, both were retrospectively conducted at larger treatment centers with considerable support from US Government and/or academic collaborators. CLADE aims to prospectively and systematically address these larger questions (i.e. superiority, cost-effectiveness of adding VL monitoring) relevant to ART roll-out on a large scale at the clinic-level in more rural settings. Thus, CLADE presents the advantage of data collected prospectively in the settings that likely are more generalizable to roll out of ART where the majority of persons in sub-Saharan Africa will be treated.

Two additional studies warrant attention with regard to treatment monitoring strategies. First, results from the DART study were presented at the 2007 Implementers Meeting. This large, multi-country clinical endpoint trial was designed to evaluate treatment monitoring strategies, structured treatment interruptions, and, later in the study, second-line simplification (15). One portion of DART specifically compared clinical and laboratory monitoring (CD4) to clinical monitoring only. For this objective, the study concluded clinical monitoring to be safe with little differences in death and disease progression between the two groups. Second, Mermin and colleagues recently published results from two prospective cohort studies in rural Uganda to evaluate the effect of a home-based ART programme on mortality, hospital admissions, and orphanhood in HIV infected persons and their families (16).

Results of this landmark Ugandan study demonstrated a home-based ART and co-trimoxazole program as part of a Home Based AIDS Care (HBAC) program was associated with a greater than 90% reduction in mortality in adults with HIV living in rural Uganda. In addition, the same study found persons receiving ART had high viral suppression (91-96% at 3-24 months) (16). While the DART and Ugandan home-based care studies demonstrate relative safety of clinical monitoring in certain scenarios, neither were primarily designed to prospectively evaluate the added utility of viral load monitoring in a clinic-based setting following standard-of-care, MOH guidelines more reflective of how ART is largely delivered in sub-Saharan Africa. Given costs of VL evaluations are falling and VL monitoring is recommended in Kenya and elsewhere throughout East Africa, prospectively studying the added utility of viral load monitoring as a primary research question is warranted. In fact, such studies are suggested by both the DART and Uganda home-based care presentation and manuscript, respectively. The DART presentation notes for public health policy recommendations that caution should be exercised in decisions based upon one trial alone (15). Additional potential limitations in HBAC due to referral biases and frequent (weekly) home visits are also addressed within the CLADE study design (16).

Closely related to the role of VL monitoring in ART roll-out and management at this stage in Kenya is the role of HIV resistance testing, now standard of care in the US (12) and also recognized and recommended by the Kenya MoH and WHO (8, 9, 17). While the role of patient-oriented resistance testing has been recognized by the MoH since 2006, the WHO, in collaboration with CDC, has recognized the importance of HIV resistance testing particularly on a population basis (8, 17). The WHO has published draft guidance for monitoring HIV drug resistance in ART roll-out in resource limited settings (17). Similarly, development of local capacity to conduct HIV genotypic resistance testing has been stressed as essential to maintaining the value of ART in the future (11).

Currently, very limited capacity and use of HIV resistance testing (primarily genotypic) exists in Kenya. As few as 3 laboratories currently have the capacity to conduct HIV genotypic resistance testing: Aga Khan Hospital, Nairobi; KEMRI/Nairobi; and, KEMRI/CDC/Kisumu, with the latter 2 conducting resistance testing primarily for epidemiological or research purposes as opposed to real-time, clinic-based testing. Due to limited availability in Kenya, clinicians often ship specimens out of Kenya for patient related HIV resistance testing (mostly to South Africa and the US).

Limited data exist within Africa regarding background and treatment-related ARV resistance. Many such studies are limited by small sample, cross-sectional methods, and/or conducted within the context a randomized controlled trial setting (18-22). Studies conducted outside of sub-Saharan Africa have demonstrated a higher likelihood of developing drug-resistant virus to be related to prevalence of drug resistance in persons engaging in high-risk behavior (ranging from 6-16% resistance to 1 ARV class and 3-5% resistance to more than one ARV class) (12, 23-27). In addition, a higher prevalence of nucleoside reverse transcriptase inhibitor (NRTI), non-nucleoside reverse transcriptase (NNRTI), and protease inhibitor (PI) resistance in non-clinical trial settings would suggest background resistance may be notable in areas of Kenya now 3-5 years in the roll-out of ART and at a point of possible 2nd line treatment failures (12, 28).

The importance of HIV resistance surveillance will be critical to not only support public health policy development by key stakeholders but to also aid in designing education and prevention programs to minimize the development of HIV resistance and support rational ART uses as PEPFAR approaches its second phase and re-authorization. Finally, given the advanced degree of ART roll-out in Kenya, real-time HIV genetic resistance testing should be available locally, and clinicians need to become familiar with its appropriate use – just as the case with CD4 monitoring earlier in PEPFAR and now more so with VL monitoring. As a secondary objective, CLADE offers the unique opportunity to collect baseline and 18-month HIV genotypic resistance testing on a population level consistent with WHO guidelines in a cohort that will be followed prospectively in a clinic-based ART setting (17). Additionally, real-time resistance testing will be available as recommended for treatment failures prior to switch to second-line therapy thereby offering improved standard of care as well as local capacity development (8, 11).

CLADE offers the opportunity to evaluate secondary questions regarding ART monitoring and roll-out as well as the feasibility of conducting such research in district-level ART clinics. In addition to limitations in resources, routine VL monitoring has not been widely implemented in many areas of rural Kenya. By utilizing KAP (knowledge, attitude, and performance) questionnaires as well as indices around VL monitoring and patient care, CLADE can evaluate the feasibility of rolling out VL monitoring (itself a routine program activity under PEPFAR care and treatment). By design, CLADE offers the chance to describe not only the degree of agreement between clinical/CD4 and VL criteria for assigning treatment failure but also the relationship between VLs obtained in the work-up of treatment failure and resulting HIV resistance testing results. Such data will be valuable in future policy decisions regarding VL monitoring and resistance testing. Finally, CLADE offers the chance to examine the feasibility of bringing such observational research in to busy ART clinics in rural Kenya, an issue that is important as larger public health evaluations are considered and planned.

With regard to HBAC and other PEPFAR PHEs, the following outlines strengths of CLADE and underscores the importance of this PHE in comparison to HBAC and other PEPFAR PHEs or studies of ART monitoring approaches:

A. HBAC was a home-based intervention using frequent visits (interventions). CLADE is the only known prospective clinic-based evaluation looking at MOH recommended evaluating strategies. Such evaluation (including cost-effectiveness analyses (CEAs) and feasibility) has been identified as a key priority in Kenya by the Kenya USG PEPFAR team, MOH, and Kenya ART National Stakeholders meeting participants.
B. The treatment and follow-up for CLADE more closely resembles that for routine care as opposed to the weekly home-based monitoring approach used in HBAC.
C. Clinics participating in CLADE represent rural, routine MOH clinics (representing the burden HIV disease and treatment) as opposed to larger treatment centers often with direct USG or academic support.
D. CLADE includes both population-based and real-time HIV resistance evaluations.
E. CLADE methods are more robust than previous PHEs using retrospective or cross-sectional methods.
F. CLADE involves well-developed and robust cost-effective methods. In contrast to other CEAs,
    i CLADE CEA analyses will have real health outcomes measured (i.e. between the two arms, both will be compared by the clinical and lab indicators of viral failure).
    ii Other studies have measured health outcomes based on morbidity, mortality and DALYS (disability adjusted Life years). Theses estimates have less weight than the actual clinical and lab indicators that will be used in CLADE (e.g. viral failure, OIs, WHO staging, viral resistance, treatment failures, etc).
    iii Other studies have calculated the incremental cost effectiveness ratio (ICER) per DALYS. CLADE will use a cost-effectiveness analysis using more robust data collected over time and covering more specific areas (as

opposed to calculating the cost-effectiveness based on an estimated measures of outcomes).

iv The health gains for CLADE will be clearly defined by the different categories of the end-points.

Consistent with recommendations from the Institute of Medicine and under the auspices of the PEPFAR program, CLADE offers the opportunity to prospectively incorporate data for critical CEAs while simultaneously evaluating the superiority of VL monitoring in clinic-based ART roll-out (29). Incorporation of key CEAs is possible based upon the longstanding and productive collaboration between KEMRI, WRP, and the Boston University (BU) School of Public Health's Center for International Development (30-36). Primary and secondary data generated from CLADE will offer both policy and ART implementing stakeholders critical information in future planning and roll-out of ART, key as the second round of PEPFAR authorization is underway with plans already proceeding for continued and expanded ART roll-out.

2. HIV/AIDS AND CARE AND TREATMENT IN KENYA AND SOUTHERN RIFT VALLEY PROVINCE

HIV/AIDS remains in epidemic proportion throughout much of Sub-Saharan Africa and with significant impact worldwide. In 2007, there were approximately 33.2 million (30.6–36.1 million) persons worldwide living with HIV/AIDS. Of note, this UNAIDS figure represents a 16% reduction compared with the estimate published in 2006 (39.5 million [34.7–47.1 million]). Among these decreases in HIV/AIDS prevalence in sub-Saharan Africa, 70% of the reductions are due to changes in six countries: Angola, India, Kenya, Mozambique, Nigeria, and Zimbabwe. Evidence suggests in Kenya that a proportion of the decline reflects a reduction of the number of new infections, in part due to a reduction in risky behaviors (37).

In addition to marked attention to prevention activities likely in part responsive for the decreases in HIV prevalence, attention continues to focus on HIV care and treatment of infected persons. A result of the focused attention on provision of antiretroviral therapy

(ART) throughout Kenya, it is estimated that 180,000 Kenyans were receiving ART in December, 2007. Furthermore, it is projected that nearly 230,000 persons will be receiving ART by June, 2008 (3). While initial ART-roll out was strategically designed with a relatively small slope to allow for capacity (human resources, physical infrastructure, and commodity procurements), the slope of Kenya's trajectory has been markedly greater with rapid scale up for the last several years. Overall GOK ART goals supported by PEPFAR have been/will be: 2005=38,000; 2006=75,000; 2007=125,000; 2008=205,000; and, 2009=270,000 (2).

The Kenya Medical Research Institute/Walter Reed Project (KEMRI/WRP) HIV program in Kericho, Kenya, in close collaboration with the MoH helped roll-out ART throughout the southern Rift Valley Province of Kenya in April 2004 covering a catchment area of approximately 2.5 million. Since then, more than 30,000 Kenyans have enrolled in newly opened HIV clinics and almost 10,000 have started ART (Figure 1). Leveraging upon existing laboratory and clinical research supported by the US Department of Defense (US DoD)/United States Military HIV Research Program (USMHRP), an early rapid response consistent with the Emergency Plan roll-out with attention to safety monitoring and capacity building was successfully launched and continues throughout the southern Rift Valley Province. Following consultation under the auspices of the Nyanza Provincial Medical Officer (PMO), US DoD/USMHRP now also works in collaboration with the Centers for Disease Control and Prevention and the United States Agency for International Development in Nyanza Province and supports HIV care and treatment services in Kisumu West District (38, 39, 40).

The KEMRI/WRP HIV program is currently supporting HIV care and treatment in eight districts of the southern Rift Valley Province (Bureti, Bomet, Kericho, Kipkelion, Nandi South, Nandi North, Sotik, and Transmara). Comprehensive HIV care and treatment including ART is provided at 11 district hospital level facilities (with KDH serving as a tertiary referral) including 7 MoH facilities, 2 faith-based facilities, and 2 plantation hospitals. Working through the network model and in developing lower rural health

centers and dispensaries, over 250 facilities are now included in the care and treatment network.

Figure 1. HIV Clinic and ART Uptake in the Southern Rift Valley Province

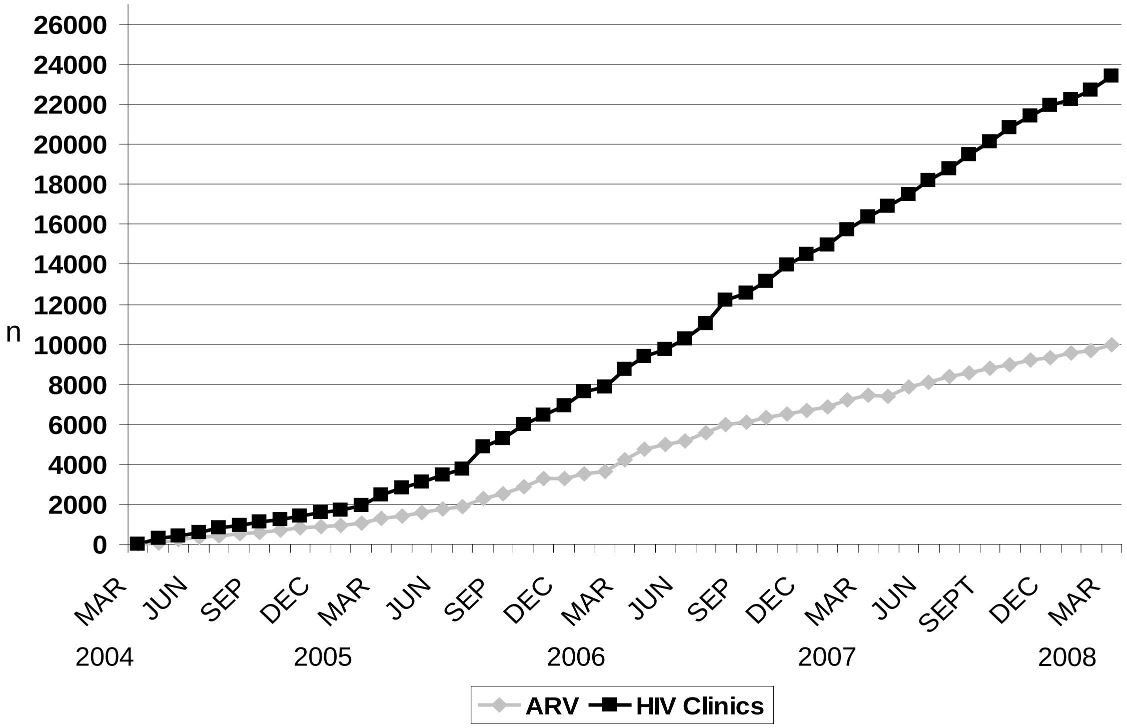

In addition to HIV care and treatment, the KEMRI/WRP HIV program supports one of the largest PMTCT programs in Kenya. Since August 2001, the KEMRI/WRP HIV Program has been implementing Prevention of Mother-to-Child Transmission of HIV infection (PMTCT) programs in the southern Rift Valley Province. The number of PMTCT sites has increased from 3 to more than 170. To date, more than 160,000 women have received PMTCT counseling and testing with > 99% receiving their results. Finally, Counseling and Testing (>100,000 tested) and smaller Abstinence-Be Faithful, Orphans and Vulnerable Children, and Home-Based Care Program are also supported.

3. STUDY AREA

The CLADE study will be conducted in western Kenya largely in 5 Districts (Bomet, Bureti, Kericho, Nandi North, and Nandi South) of Kenya's southern Rift Valley Province with one site in Kisumu West District of Nyanza Province adjacent to Lake

Victoria. The African Highlands of the southern Rift Valley Province are approximately 2300 meters above sea level about 90 km southeast from Kisumu/Lake Victoria and are known for rolling tea fields. Many in the region live on subsistence farming, with primary industries in the region including tea and, at lower elevations towards Kisumu, sugarcane. While national estimates for HIV prevalence in adults aged 15-49 for rural, Rift Valley are between 5.3 and 5.6%, regional variability exist (41). On a tea plantation outside of Kericho, a prospective cohort study of 2,801 plantation workers and dependents aged 18-55 noted a considerably higher baseline prevalence, 14.3% (19.1% women and 11.3% men) (42). Variability may, in part, be explained by differing methods used and regions tested.

The study site along Lake Victoria is in Kombewa, a small town near the northeast shore of Lake Victoria and 40 km northwest of Kisumu. Most inhabitants of Kombewa live by subsistence farming. HIV prevalence in and around Kisumu, including Kombewa, is among the highest in Kenya with a prevalence of 15.1% in adults aged 15-49 and as high as 26.1% in earlier antenatal clinic estimates (41, 43). The ethnic tribe largely predominate in Kisumu is the Luo. In contrast, the ethnic tribe most common in the southern Rift Valley is the Kalenjin (with Nandi and Kipsigiis sub-tribes). Other tribes common in the southern Rift Valley include the Kisii, Luhya, and Luo among others with smaller presence.

4. COLLABORATING INSTITUTIONS

CLADE brings together expertise from both Kenya and US institutions built upon decades of collaboration. The KEMRI was established in 1979 to represent the national body responsible for carrying out health science research in Kenya (44). The 13 Research Centres of KEMRI are intended to focus on certain specific areas of national and/or strategic importance covering all infectious and non-infectious research categories. The WRAIR/USAMRU-K (also known as the Walter Reed Project/WRP) has had a long collaborative relationship with KEMRI after coming to Kenya in on a temporary basis in 1969 at the invitation of the Government of Kenya to study trypanosomiasis (45, 46). Given the breadth and depth of HIV recognized in Kenya combined with a primary

mission of development of a globally effective HIV vaccine, the WRAIR/Division of Retrovirology/USMHRP in close collaboration with KEMRI developed a research field station in Kericho, Kenya, in 1999 (29).

Building upon international expertise in HIV epidemiology, cohort, and vaccine research, the USMHRP has worked in Kericho with KEMRI to conduct Kenya's largest prospective HIV cohort study to date, the first and largest Phase I/II HIV vaccine study ever outside of Nairobi, and the largest and fastest enrolling site in an US National Institutes of Health (NIH)/Adult AIDS Clinical Trials Group (ACTG) multi-African-country Phase III therapeutics study in addition to rolling out HIV care and treatment in the larger southern Rift Valley Province (47, 48, 49). Finally, the USMHRP/WRP in Kericho has had a > 5 year collaborative relationship with BU in conducting pivotal health economics research related to HIV/AIDS (33-39). Together, these institutions bring together expertise to conduct CLADE with all PHE aspects (clinical, laboratory, and data analyses) being conducted in-country reflecting the commitment and success in technology transfer and capacity development in Kenya.

IX. EVOLVING TREATMENT AND MONITORING GUIDELINES

From protocol development to study initiation (2007-2010), updated and new guidelines for treatment and monitoring have been developed as to be expected. Revised guidelines for ART in adults have been published by the WHO and the Kenya Ministry of Health has subsequently provided memoranda for implementation (50, 51). Most notable for ART, the CD4 count for initiating ART has been increased to 350 cells/mm^3 with certain caveats where ART may be initiated at higher CD4 counts (e.g. Tb regardless of CD4 count). In addition, tenofovir has been added as a first line agent. For purposes of the CLADE study, eligibility focuses upon the primary care clinician intending to start first line ART and such changes anticipated to be distributed equally between both arms by randomization will not impact the primary or secondary endpoints.

In addition, the use of “targeted” viral load monitoring has been introduced in Kenya by the MoH. Given viral load monitoring was standard of care in evaluation of treatment failures in the study region, such monitoring had been previously recognized and ongoing in this study (See Section XIII.3.b.). For research purposes, the CLADE study will have an opportunity to estimate the impact of differing guideline definitions of virological failure (e.g. WHO defines viral failure at >5,000 copies/ml and the Kenya MoH guidelines define virologic failure as >1,000 copies/ml). For clinical purposes for this study following Kenya MoH guidelines standard of care, virological failure will be defined as >1,000 copies/ml.

An additional WHO guidance documents warrants discussion and has been incorporated into the study. Growing attention to the magnitude of ART roll-out in Africa and elsewhere with the associated risk of HIVDR has prompted the WHO to provide guidance for monitoring HIVDR “Early Warning Indicators.” (52). The purpose of HIVDR EWI monitoring is to assess the extent to which ART programmes are functioning to optimize prevention of HIVDR. EWIs measure ART site factors known to be associated with good programme functioning and the prevention of the emergence of HIVDR. The guidelines provide indicators for both pediatric and adult populations. While the guidelines provide 6 recommended EWIs and 2 optional EWIs, all 8 are being addressed either directly through research purposes of the CLADE study or indirectly through routine HIV care and treatment roll-out by the KEMRI/WRP HIV Program. These 8 EWIs include:

1. ART prescribing practices (determine the proportion of adults prescribed correct first line ART)
2. Patients lost to follow-up 12 months after ART initiation
3. Patients on appropriate first-line ART at 12 months
4. On time ART pick up
5. ART clinic appointment keeping
6. ART drug supply continuity (percentage of months in a designated year which there were no ART stock-outs)
7. Patient ART adherence

8. Viral load suppression 12 months after ART initiation

The CLADE study will have the ability to evaluated the above 8 EWIs and will do so at the 6, 12, and 18 month scheduled follow-ups.

More recently, data from a cluster randomized trial of routine vs. discretionary viral load monitoring among adults starting ART in Zambia were presented by Saag and colleagues at the 19th Conference on Retroviruses and Opportunistic Infections (CROI) held March 5-8, 2012 (53). This study was conducted at 12 primary care clinics in Lusaka, Zambia and compared the effect of routine viral load monitoring to the standard of care where VL is used sparingly to adjudicate discrepancies between CD4 and clinical assessments. The authors concluded routine VL monitoring did not reduce all-cause mortality over the first 36 months of ART. Routine VL, however, resulted in earlier regimen change and may have an important triaging role for targeted adherence support measures, including intensive patient counseling. Finally, given the median time to ART switch was also significantly shorter in the routine VL monitoring vs. standard of care monitoring arm (471 vs. 694 days, $p = 0.0214$), they concluded additional work is needed to understand the implications of delayed switching on ART drug resistance. Of note, the authors also presented data regarding virologic failure/rebound with and without confirmatory testing.

CLADE will provide important data in addition to the Zambia study presented by Saag and colleagues above. Of note, CLADE will be the only study randomizing within each participating site (where the Zambia study utilized cluster randomization). CLADE is also conducted in non-urban setting such as Lusaka. As secondary outcomes, CLADE will have data regarding time to ART switch as well as HIV resistance testing at time of ART switch. This HIV drug resistance data at time of ART switch is needed (as noted by Saag and colleagues above). Furthermore, important secondary analyses from the CLADE study will contribute to the body of knowledge evaluating CD4 testing (i.e. routine care arm, those who at no point had a VL) and the impact of confirmatory VL testing, particularly given the new WHO guidelines of targeted VL monitoring outlined above.

X. DESIGN

CLADE is an unblinded, randomized (1:1), prospective, observational, cohort public health evaluation (PHE) aimed at evaluating the superiority and cost-effectiveness of two recommended MoH ART diagnostic evaluation approaches at the clinic level in adult treatment naive patients beginning MoH approved first-line ART (8, 9) (Appendix I). Two arms exist in the study. Arm A or "routine care" will represent the most common approach to ART roll-out where clinical (World Health Organization (WHO)) staging and immunological (CD4) monitoring are the primary baseline and follow-up evaluations with targeted viral load monitoring. Arm B or "viral load guided care" will incorporate routine VL monitoring with clinical and immunological evaluations.

A total of 820 adult patients initiating first line ART will be recruited. 410 participants entering Arm A/Routine Care will receive MoH standard of care monitoring consisting of baseline CD4 and WHO staging every 6 months, or as clinically indicated, with CD4 and WHO staging criteria guiding care and treatment in addition to routine clinical evaluations. 410 patients entering Arm B/Viral Load Guided Care will receive MoH standard of care as in Routine Care/Arm A but also have VL monitoring at baseline and every 6 months, or as clinically indicated, to guide care and treatment.

Participants entering each arm will receive Kenya MoH recommended standard of care, first and second-line ART as indicated (8, 9, 51). Revised first-line ART regimens in Kenya are shown below in Table 1, and second line ART regimens are shown in Table 2 (51).

Table 1. First Line ART in Kenya

**Tenofovir (TDF) or zidovudine (AZT)**<br>+<br>**lamivudine (3TC)**<br>+<br>**nevirapine (NVP) or efavirenz (EFZ)**

Those qualifying for second-line therapy will receive 2 new NRTIs and a Protease Inhibitor (PI) as recommended by the MoH as below in Table 2.

Table 2. Second Line ART in Kenya

**tenofovir (TDF) + lamivudine (3TC) + ritonavir boosted lopinavir (LPV/r)**<br><br>**or**<br>+<br>**Zidovudine (AZT) + lamivudine (3TC) + ritonavir boosted lopinavir (LPV/r) or ritonavir boosted atazanavir (ATV/r)**

Guidelines note: for patients intolerant of AZT, d4T may be used; and, for patients intolerant of LPV/r, ATV/r may be used.

Prior to enrollment, patients will receive routine standard of care laboratory evaluations (see Section XIII, Laboratory Evaluations) and HIV and adherence counseling as well as routine addition HIV care medicines as clinically indicated (see Section XII, Clinical Care).

Cost-effective analyses (CEA) comparing VL guided care (Arm B) versus routine care (Arm A) will proceed broadly evaluating costs of treatment success (See XV.6.b). Within the prospective design, key secondary analyses will include assessment of the feasibility of rolling out routine viral load monitoring, comparing clinical disease progression between the two monitoring strategies, describing resistance patterns in cases of treatment failure as well as the relationship between the viral load and subsequent resistance patterns demonstrated, and adherence to GCP by clinics participating in CLADE (See XV.6.a). Based upon the availability of the cohort, population-based resistance testing will be conducted (See XV.6.a).

Finally, 2 retrospective analyses will be done: 1. retrospective VLs will be run at time points in Arm-A (routine care) when CD4 counts were obtained in an effort to ascertain

agreement between the CD4 and VLs with regard to treatment failure and viral suppression; and, 2. retrospective VLs will be run on stored samples obtained at the month 3 (week 12, visit 5) visit to assess viral suppression following ART initiation. Both of these evaluations are for research purposes only given these are not part of routine care at this time in Kenya. Therefore, they will be conducted retrospectively (See XV.6.a).

XI. STUDY SITES AND ALLOCATED ENROLLMENT

The CLADE study will be conducted at 7 ART facilities (5 MoH and 2 mission/faith-based) with assigned enrollment proportions based upon facility size, capacity, and HIV burden. Site selection has been conducted in consultation with the MoH, KEMRI, and the actual ART clinics/sites. The aim in site selection was to provide sites already participating in ART rollout in the larger SRV the opportunity to participate in CLADE (including more experience in VL monitoring and treatment failure assessments), obtain a cross representation of clinics and persons in this rural region, and assure adequate sample size enrollment. It is appreciated that no one site will have adequate sample size/power for primary outcome analyses. Table 4 below presents characteristics of CLADE sites, and Appendix II depicts the study sites and enrollment allocations with regard to their regional location.

*Table 4. Characteristics of CLADE Clinic Sites**

Name	District (Population / Catchment) †	HIV Clinic Size (on ART)	HIV Clinic Enrollment (n/mo)	ART Enrollment (n/mo)	CLADE Enrollment
**Kericho District Hospital††**	Kericho (400,000)	7000 (3000)	160	40	150
**Kisumu West District Hospital**	Kisumu West (150,000)	2700 (800)	120	40-100	190
**Kapkatet District Hospital**	Bureti (320,000)	1600 (450)	40	16	90
**AIC Litein Mission Hospital**	Bureti (320,000)	1500 (700)	60	23	90
**Kapsabet District Hospital**	Nandi North (320,000)	2000 (700)	70	23	110
**Nandi Hills District Hospital**	Nandi South (250,000)	1600 (600)	45	20	90
**Tenwek Mission Hospital**	Bomet (280,000)	2500 (700)	60-70	20	100

* HIV clinic estimates (e.g. size, enrollments) based upon routine uptake reports
† See reference #54
†† See reference #55

XII. ANTIRETROVIRAL THERAPY AT STUDY SITES

All ART supplied by PEPFAR used at the study sites have been recommended for use by the Kenya MOH (8, 9) and the World Health Organization (56) and have been approved by the Kenya Pharmacy and Poisons Board for import and use in Kenya (57). In addition, all ART purchased with PEPFAR funds as individual drugs or fixed dose combinations have been approved by the US Food and Drug Administration (FDA) either by routine review and approval or under the expedited review process (58, 59, 60). Finally, all ART utilized at treatment sites and purchased with PEPFAR funds continue to

be recognized for use in the US under the Department of Health and Human Services' Antiretroviral Guidelines for Adults and Adolescents (12).

In addition to a long history of demonstrated safety and efficacy (albeit with recognized toxicities managed in a risk/benefit relative to treatment of HIV under the practice of medicine) in the US (12), all ART components have now been used in Kenya in some areas for almost 5 years as the first 5-year round of PEPFAR comes to an end. Approximate ART use (in patient-years dispensed) in Kenya prior to study opening is provided below in Table 5 based upon information from Management Sciences for Health (MSH) (60).

*Table 5. Experience and Use of ART in Kenya**

**Antiretroviral Drug**	**Use (patient-years) To Date**
stavudine (d4T)	192,000
zidovudine (AZT)	40,000
lamivudine (3TC)	225,000
efavirenz (EFZ)	59,000
nevirapine (NVP)	167,000
tenofovir (TDF)	4,000
didanosine (ddI)	3,000
abacavir (ABC)	2,000
lopinavir boosted ritonavir (LPV/r)	4,000

* including all dosages and formulations; rounded to nearest thousand

XIII. CLINICAL CARE PROVIDED AT STUDY SITES

Clinical care including ART, non-ART, HIV co-morbidities, and non-HIV related medical conditions will fall under the standard of care and practice of medicine in Kenya.

1. PRE-ART (PRE-RANDOMIZATION) CARE

The Kenya MoH ART guidelines recognize the importance of key activities (e.g. HIV education, nutrition counseling, adherence counseling) prior to ART initiation (9). The guidelines emphasize that unnecessary barriers should not exist that may prevent persons in need of ART from receiving such treatment. Three general categories of activities are recommended prior to ART initiation: 1. diagnosis of HIV infection (see A below); 2. patient evaluation and preparation for ART (including medical assessment and WHO staging, laboratory evaluation; and treatment & prophylaxis of opportunistic infections (see B below); and, 3. patient education and psychosocial assessment and preparation for ART (e.g. adherence counseling, HIV and ART education, see C below). Such pre-ART assessments and interventions routinely occur during 1 month prior to ART initiation, although variation is accepted as clinically indicated. Appendix III demonstrates how these activities are conducted independently and equally for persons who may or may not enter CLADE.

A. Diagnosis of HIV infection

While rapid tests are most frequently used in diagnoses if HIV, Kenya MoH guidelines also recognize ELISA (9, 61, 62). For patients presenting to ART clinics, HIV testing has been routinely completed at an outside facility (e.g. Voluntary Counseling and Testing [VCT], mobile VCT, or in/out-patient Provider Initiated Testing and Counseling [PITC]). Rarely, ELISA is used. For purposes of CLADE, confirmation of positive HIV testing is an inclusion criteria. As indicated, clinicians have the opportunity to repeat HIV testing if clinically indicated.

B. Patient evaluation and preparation for ART

Patient evaluation and preparation for ART is recommend and utilized at all treatment sites. Such evaluation and preparation includes routine medical evaluation and WHO staging, laboratory evaluation, and treatment &

prophylaxis of opportunistic infections. Standardized intake encounter forms are used at each site for initial medical evaluations, and WHO staging criteria are used (63).

As part of standard of care, the following laboratory tests are recommended as minimum: hemoglobin (Hgb), alanine aminotransferase (ALT), CD4 count, and pregnancy test in adults and adolescents if EFZ is to be used. Other "desirable" tests include a complete blood count (CBC), liver function tests (LFTs), creatinine (Cr), amylase/lipase, serum lipids, cervical cytology, VDRL, and hepatitis B&C serology. Of note, no sites routinely conduct all of the above desirable tests.

Patients presenting with symptoms suggestive of pulmonary tuberculosis should have sputum sent for Acid Fast Bacilli (AFB) staining and a Chest X-Ray (CXR). Of note, the Kenya MoH guidelines recommend "viral load (VL) where available should be done at baseline; VL is not necessary for treatment initiation." The guidelines emphasize, "Lack of laboratory tests should not be a barrier to initiation of ART in symptomatic patients who qualify for treatment"(9).

Finally, standard of care according to guidelines emphasizes appropriate treatment and/or prophylaxis of opportunistic infections. Specifically, all persons with HIV/AIDS should receive trimethoprim/sulfamethoxazole prophylaxis and start multivitamins daily. Review of immunizations is recommended, and fluconazole prophylaxis is recommended for persons with a history of cryptococcal meningitis as outlined in guidelines (9). Of note, isoniazid preventive therapy (IPT) is recommended in "approved" facilities. IPT is not currently prescribed at any study site consistent with MoH leadership (PARTO, PMO) recommendations. All patients will have patient evaluation and preparation confirmed as part of study inclusion criteria.

C. Patient education and psychosocial assessment & preparation for ART

Patient education and psychosocial assessments/preparation are now standard at all study sites. Dedicate staff, often including Persons Living With HIV/AIDS (PLWA), give attention to these activities before and during ART. Patient education broadly covers HIV disease, ART, opportunistic infections, CD4 counts, disclosure of HIV status, and participation in the patient's management plan. Psychosocial assessments are conducted to assure a patient is ready, willing, and able to start and continue ART. Finally, but not all inclusive, adherence counseling is considered essential and continues at each patient encounter. All patients will have patient education and psychosocial assessment & preparation for ART confirmed as part of standard of care.

2. ART (ON-STUDY) CARE

Kenya MoH guidelines recognize 5 general categories of activities to be conducted in follow-up of patients on ART: 1. clinical monitoring; 2. laboratory testing; 3. adherence assessment and strengthening; 4. psychosocial assessment; and, 5. medication prescribing and dispensing. Appendix IV graphically demonstrates how these activities are routinely conducted broadly for persons regardless of participation in CLADE. While any such guidelines are intended for guidance, it is important to recognize depending upon the clinical scenario that all, few, or more extensive activities may be completed for any one patient or visit.

A. Clinical monitoring

Components of clinical monitoring focus upon review of constitutional symptoms suggestive of illness, ART tolerability and adverse events, and other signs or symptoms suggestive of pathology. Each patient routinely has a focused physical examination, and vitals and patient weight should be recorded on each visit.

B. Laboratory testing

General laboratory testing is outlined in Kenya guidelines; however, discretion and adaptation are recognized as clinically indicated. Hgb, ALT, Cr, urinalysis (UA), and CD4 count are recommended every 6 months with Hgb and ALT being conducted more frequently for persons receiving AZT and NVP, respectively, as outlined. Of note, VL monitoring is recognized in follow-up of patients where available.

C. Adherence assessment and strengthening

Adherence assessment and strengthening occurs at every follow-up for patients receiving ART. While pill counts are recommended, it is recognized by the MoH that this is not feasible at every visit given clinic size and capacity on any one day. In the event pill-counts are not conducted, self-reported adherence is recorded. Efforts are made to understand reasons behind non-adherence with education and reinforcement regarding the importance of adherence (and correlated to clinical [weight] and biological [CD4 count, VL if possible] markers).

D. Psychosocial assessment

Psychosocial assessments at each visit include broadly evaluation of activities of daily living, signs or symptoms of depression, substance abuse, physical activities, and finally disclosure of HIV status where appropriate. Psychosocial assessment occurs at each treatment site and may be conducted by a range of member in the health care team. PLWA as well as HIV support group members are playing increasing rolls in psychosocial support. Both are available at each study site.

E. Medication prescribing and dispensing

ART is prescribed by medical officers and clinical officers in Kenya. Following pill counts and adherence counseling, ART will be prescribed and dispensed. Each study site has a ART pharmacy and dedicated pharmacist and/or pharmacist technician who dispense ART and also provide ART education. As part of ART dispensing, patients are to be counseled regarding avoidance of herbal medicines and/or self-medication. Of note, medication prescribing and dispensing is not conducted on every visit now that sites are moving toward dispensing of multi-month regimens where stocks permit and patients demonstrate appropriateness.

3. TREATMENT FAILURE

Evaluations of persons receiving ART for treatment failures are of marked importance in the management of persons receiving ART and are key components in CLADE. The MoH ART guidelines note that treatment failure should be suspected if there is a clinical or immunological deterioration (9). Where suspected, a review should be conducted of clinical history and CD4 trends. Viral load monitoring is noted to be helpful particularly where clinical and immunological indicators are not conclusive. As part of suspected treatment failures, particular attention is given to the mandatory adherence review. Given limited second line therapies available in Kenya, emphasis is given to maximum longevity of first line therapy. Table 6 below outlines Kenya MoH definitions for treatment failures in adult patients based upon clinical, immunological, and virological parameters (51).

Table 6. Treatment Failure Definition in Adults

Treatment Failure Parameter	Criteria
1. Clinical Failure	New onset of significant OIs or malignancy, usually a WHO stage 3 or 4 condition
	Recurrence of previously treated OIs after at least 6 months of ART

	Downgrading of WHO classification in the course of follow-up
2. Immunological Failure	CD4 count falls to or below pre-ART level
	CD4 counts falls by 30% of more from treatment peak value
	CD4 remains persistently below 100 cell/mm3 after at least 12 months of effective ART
3. Virological Failure	Viral load > 1000 copies/ml

Note: any one criteria for clinical or immunological failure may signify treatment failure. Non-adherence remains the main cause of treatment failure and should be addressed as a priority. If treatment failure is on clinical grounds, then a CD4 should be checked and repeated if necessary in the face of an intercurrent illness with attention to the illness. Targeted viral load monitoring should proceed in the event any of the immunological criteria are observed.

Given limited use and experience of VL monitoring in Kenya overall, objectives of CLADE are to increase awareness of viral load monitoring as it may relate to treatment failure assessments and management. All clinicians and study teams at each treatment site will receive education prior to study opening and ongoing support with regard to VL monitoring. Overall, treatment failure assessments and subsequent management will be addressed similarly in both the Routine Care (Arm A) and Viral Load Guided (Arm B) arms as outlined by MoH guidelines (9). However, introduction of scheduled viral loads with algorithm guided management will be utilized for Arm B.

A. Routine Care (Arm A) Treatment Failure Assessment and Management

Patients presenting with signs and symptoms suggestive of treatment failure will be evaluated based upon clinical and immunological criteria outlined above (Table 5). Reflecting the recognized scenario where routine VL monitoring is not available as is the most common practice in Kenya, subsequent management will rely primarily upon

current MoH guidelines for changing to second line therapy as indicated in Table 6 below (8).

Table 7. Principles and Criteria for Changing to Second Line ART

Do not rush to second line treatment
Discuss patients in a multi-disciplinary team meeting
Assess adherence and address issues prior to changing to second line. If adherence can not be improved, attempt directly observed therapy
In patient with weight loss, consider tuberculosis
If pulmonary tuberculosis is present, look for other clinical, immunological (and virological) evidence
Development of Immune Reconstitution Inflammatory Syndrome (IRIS) should not be considered as treatment failure
When initiating second line therapy, review ART history for preventive therapy for Mother to Child Transmission
The new regimen should include as may active new drugs as possible; change the entire regimen where possible
Review all other medications for drug interactions with new ARV drugs
Do not discontinue ART regimen until the new regimen becomes available

Of note, while the premise of the primary hypothesis is equipoise exist with regard to routine VL monitoring in preventing viral failures (and secondarily changes to second line therapy), the study team recognizes the ethical obligation to offer VL testing to patients in Arm A given the availability and potential finding that VL monitoring decreases viral failure (and subsequent change to second line therapy). To that end, all patients classified as treatment failures in Arm A (by clinical and/or immunological criteria as above) will be censored for primary endpoint analyses and offered VL monitoring as further discussed in Sections XV (Statistical Considerations) and XVIII

(Human Subjects Protection). All patients in Arm-A will have targeted viral load monitoring based upon most recent MoH guidelines (51). In addition, all patients on Arm-A will have viral load monitoring at the 18-month, final study visit also addressed further in Sections XV.3 and 7 (Statistical Considerations, Endpoints, Analyses).

The rational for censoring subjects in Arm-A is based upon the position of the study team that any person on Arm-A who is suspected of having treatment failure based upon clinical and immunological criteria (Table 5) ethically must have VL testing (Appendix V). Currently, VL monitoring is offered to all clinicians in the region who suspect patients of having treatment failure. In addition, Arm-B utilizes routine VL monitoring per MOH guidelines. Therefore, not offering VL to patients receiving ART would be a sub-standard of care.

For purposes of the study, those on Arm-A who receive a VL as part of evaluation for treatment failure will continue on Arm-A and not receive additional VL monitoring as part of the study. Regarding censored subjects and analyses, three end-point analyses will be conducted to demonstrate the impact and potential biases imparted by censoring: first, an analysis excluding these censored subjects; and second and third, analyses including these subjects based upon VL (see Statistical Considerations, Section XV).

Real-time resistance testing will be conducted on all persons changing to second line therapy. For patients categorized as having first-line ART treatment failure, the CLADE team will meet with the ART treatment team to review all clinical and laboratory data prior to any switch to second-line ART.

B. Viral Load Guided Care (Arm B) Treatment Failure Assessment and Targeted Viral Load (Arm A) Algorithm and Management

Patients in Arm B will follow the same initial assessment and management principles as those in Arm A; however, they will also use routine VL monitoring as well as use an algorithm modified from MoH guidelines to guide VL monitoring, assessments, and

subsequent clinical decisions (9) (Appendix V). Viral load monitoring will occur routinely on Arm B and by following targeted viral load monitoring for Arm A. Two successive viral load failures (defined as > 1000 copies/ml), with the second occurring 3 months after the initial failure, confirms treatment failure. At that point, a patient is prepared for switch to second line ART with continued adherence attention (51). Real-time resistance testing will be conducted on all persons changing to second line therapy. For patients categorized as having first-line ART treatment failure, the CLADE team will meet with the clinic ART treatment team to review all clinical and laboratory data prior to any switch to second-line ART. The respective MoH Multidisciplinary Team will be convened prior to switch to second line therapy per MoH guidelines (51). In addition, the DMC will be presented with summaries of all second-line therapy changes (see XVII, Data Monitoring Committee).

4. Additional Clinical Care

Additional HIV and non-HIV related clinical care services are provided at all sites participating in CLADE. HIV-related care is supported by MoH and PEPFAR. Services offered at each treatment site participating in CLADE include diagnosis and treatment of common opportunistic infections (e.g. cryptococcal meningitis, pulmonary tuberculosis) and chemoprophylaxis (e.g. trimethoprim/sulfamethoxazole, fluconazole). All patients in HIV clinics are offered these services. Non-HIV related clinical care is also provided at all study sites at standards in Kenya for district-level facilities, although some charges apply. Examples of non-HIV related care offered at the study sites include treatment of malaria and other common non-HIV related infections (e.g. diarrhea), cardiovascular diseases (e.g. diabetes, hypertension), and general surgery.

XIV. LABORATORY EVALUATIONS PROVIDED AT STUDY SITES AND ADDITONAL STUDY EVALUATIONS

1. HIV pre-ART and Routine Follow-up Laboratory Evaluations

While ELISA is recognized in Kenya's ART guidelines, HIV rapid tests are used most commonly in the diagnoses of HIV as outlined in Kenya guidelines (9, 61, 62). Currently, a sequential testing algorithm is used with Determine followed by Bioline rapid HIV tests and Unigold used as the tie-breaker. All sites participating in CLADE use HIV rapid tests for HIV diagnosis. HIV status confirmation by ART clinics may be a referral letter to the HIV clinic, counseling and testing (VCT) documentation, or other documents accepted by the MOH. The option of repeat HIV testing is available in all HIV clinics.

All HIV clinics participating in CLADE have the capability to conduct basic chemistry and CBC laboratory evaluations. The Kisumu West District Hospital HIV clinic is currently receiving support from the KEMRI/WRP Muritti-Wellde CRC laboratory approximately 100 meters away from the hospital. As part of standard of care, the following laboratory tests are conducted at each facility prior to ART initiation: Hgb, ALT, CD4 count, and pregnancy test in adults and adolescents of reproductive potential if EFZ is to be used. As clinically indicated, additional tests may include a CBC, LFTs, Cr, amylase/lipase, serum lipids, VDRL, and hepatitis B&C serology.

Routine follow-up laboratory evaluations are conducted at all treatment sites participating in CLADE as outlined in MoH ART guidelines. Hgb, ALT, Cr, and UA are evaluated at baseline and every 6 months for persons considered to be stable or as clinically indicated consistent with MoH guidelines. A UA is recommended at month 3. Hgb and ALT are recommended more frequently for persons receiving AZT and NVP, respectively. For patients receiving NVP, ALT is recommended at 2 weeks and 1, 2, 3, 6, and 12 months and every 6 months thereafter unless clinically indicated otherwise (9). For patient's receiving AZT, Hgb is recommended at 1, 3, 6, and 12 months and every 6 months thereafter unless clinically indicated otherwise (9).

2. CD4 TESTING

CD4 evaluations are conducted at baseline and every 6 months for all persons on ART as recommended (9). The Kericho KEMRI/WRP CRC Laboratory initially conducted all CD4 evaluations for all sites. This capacity has been transferred to local hospitals (e.g. Kericho District Hospital, Nandi North District Hospital, Tenwek Mission Hospital), and the KEMRI/WRP PEPFAR program continues to develop capacity at other facilities (e.g. Kapkatet District Hospital, Kisumu West District Hospital). CD4 evaluations will be conducted at such local hospitals where capacity exists. Where the capacity does not yet exist, the KEMRI/WRP CRC laboratory will continue to conduct CD4 evaluations. The KEMRI/WRP CRC Laboratory uses the BD FASCaliber for CD4 evaluations and has been enrolled in CD4 external quality assurance programs (College of American Pathologists, Canadian QASI, and UKNEQAS) for approximately 3 years. The KEMRI/ WRP CRC Laboratory continues to provide QA/QC support and act as the regional reference laboratory for all the PEPFAR Sites.

3. VIRAL LOAD MONITORING

Viral load monitoring will occur as targeted monitoring (Arm A) and routine (Arm B) based upon Kenya MoH ART guidelines (8, 9, 51). The KEMRI/WRP CRC Laboratory provides VL monitoring for the larger southern Rift Valley Province as the recognized reference and referral laboratory. Retrospective VL monitoring will be conducted on baseline and CD4 count stored plasma from participants in Arm-A as well as at month 3 for both arms (See Section XV, Statistical Considerations). The KEMRI/WRP CRC Laboratory currently conducts VLs by HIV-1 reverse transcriptase PCR reaction using the Amplicor HIV-1 Monitor Test, v1.5, Roche Diagnostic, Indianapolis, Indiana. Given the Amplicor v1.5 is being phased out of production, the KEMRI/CRC laboratory is moving to new 2 platforms: Abbott (certified and in use) and Roche Taqman (anticipated to be in use in approximately 6 months). For all clinical and research activities supported by the KEMRI/WRP CRC Lab, VLs will be run using a viral load platform approved in the KEMRI/CRC Laboratory under the College of American Pathologist (CAP) and related external quality assurance programs. The laboratory has been enrolled in VL external quality assurance programs (Walter Reed Army Institute of Research, VQA, and

College of American Pathologists) for approximately 5 years and has achieved CAP accreditation twice (most recently June 2010).

4. HIV GENOTYPIC RESISTANCE TESTING

For epidemiological, population-based purposes, HIV genotypic resistance testing will be conducted on a random sub-set of patients at baseline as further discussed in the Analyses section (Section XV.7a). In addition, real-time HIV genotypic resistance testing will be performed on all treatment failures prior to initiating second line therapy. HIV resistance testing will be conducted at the KEMRI/CDC/Kisumu laboratory. The KEMRI Kisumu and Kericho laboratories along with the Academic Model for the Prevention and Treatment of HIV/AIDS (AMPATH) laboratory in Eldoret have a 3-way memorandum of understanding to serve as back-up laboratories for each other. Such HIV genotypic resistance testing will proceed under the auspices of that MOU. Currently, the KEMRI-Kericho laboratory conducts viral load testing for AMPATH for a Phase III clinical trial. Similarly, it is anticipated that the KEMRI-Kisumu laboratory will conduct HIV genotypic resistance testing for the CLADE study until capacity is available in the KEMRI-Kericho laboratory (anticipated in early 2009).

In the CDC/KEMRI HIV Research laboratory, the Viroseq HIV-1 genotyping system (Celera Diagnostics, Alameda, CA, USA and Abbott, Wiesbaden, Germany) is utilized for detection of drug resistance mutations. The Viroseq assay amplifies a 1.8 kb fragment in the *pol* region of HIV-1 genome that comprising the entire protease gene and most of the reverse transcriptase gene. Genotypic analysis of this region of HIV-1 facilitates the study of the relationship between mutations and viral resistance to anti-retroviral drugs, specifically the protease and RT inhibitors.

Plasma samples will be processed according to CDC's HIV-R SOP 204 to get plasma that is subjected to genotyping utilizing SOP HIV-R 464 genotyping. Briefly, the assay involves RNA extraction and reverse transcription and subsequent polymerase chain reaction (PCR) amplification. For detection, 1.8 kb PCR products are visualized with UV

after ethidium bromide staining. The PCR products are sequenced using custom primers (six or seven different primers) formulated with the Big Dye Terminator sequencing chemistry. The sequencing products will be analyzed on the ABI PRISM 3100 Genetic Analyzer (Applied Biosystems, USA).

The sequences obtained from the ABI 3100 will be analyzed for drug resistance mutation employing SOP HIV-R 467 (Sequence data analysis using the Viroseq HIV-1 genotyping software V2.6) after initial analysis by DNA sequencing analysis software V3.7 for DNA base calling. Briefly, the ViroSeq software V2.6 is used to import and assemble sequence data into a consensus sequence, which is then compared to the reference strain to determine mutations present in the sample. The software generates a drug resistance report which provides supplemental information regarding the potential HIV-1 susceptibility to anti-retroviral drugs. The report will be submitted to clinician for making clinical decisions for treatment.

The SQUAT program (SOP HIV-R 468) will be used as Sequence Quality Assessment tool. (SQUAT) assesses the quality of a HIV genome fasta sequence (or a set of sequences) prior to sequence data analyses. Protease and/or RT sequences are loaded into SQUAT either individually or in batches. The tool aligns, translates, and screens the nucleotide sequence for frame shifts, insertions, deletions, stop codons, ambiguous characters and atypical mutations. Lastly, pairwise genetic distances are measured to identify outliers. The SQUAT output includes a summary report indicating approved and non-approved sequences with detailed information based on the screening categories, a histogram of pairwise genetic distances with phylogenetic tree, and amino acid sequence files for subsequent analyses. The Stanford HIV-Drug resistance database is further utilized to confirm the genotypic profile results and mutation detection from the sample fasta sequence. The current (International AIDS Society–USA Update of the Drug Resistance Mutations in HIV-1 is utilized as a guide to inform the current mutation classification for various drug categories (Johnson et al., 2008*)*.

The KEMRI/WRP Kericho laboratory is developing such HIV genotyping capacity as the reference laboratory for the Rift Valley Province. These HIV genotyping analyses will be conducted at the KEMRI/WRP laboratory once capacity is available. Both the KEMRI Kisumu and KEMRI Kericho laboratories will continue to serve as back-up laboratories (including HIV genotype resistance testing) under the existing MOU (including the AMPTH laboratory in Eldoret as well).

XV. CRITERIA FOR STUDY DISCONTINUATION

Participation in CLADE is entirely voluntary. Any participant may discontinue study participation at any time, and the study may be terminated at the discretion of the sponsor or regulatory bodies.

Reasons for study discontinuation include:

1. For any participant, the participant may withdrawal from the study at any time he/she would not like to participate and have data extracted from his/her medical records. Data already extracted and up to the time of withdrawal will continue to be kept in the study database for analyses.
2. For any participant, discontinuation may proceed at the request by the clinic ART team if the team thinks the study is no longer in the best interest of the subject.
3. The study overall may be terminated at the discretion of the IRBs (KEMRI, WRAIR,), the Kenya Ministry of Health (MoH), the study sponsor (Office of the Global AIDS Coordinator/PEPFAR), or the study Co-Chairs based upon recommendations from the Data Monitoring Committee (See XVII) or other scientific or safety information that becomes available that may, at the discretion of these institutes or individuals, warrant study discontinuation.

XVI. STATISTICAL CONSIDERATIONS

1. General Design

CLADE is an unblinded, randomized (1:1), prospective, observational, cohort public health evaluation. As a primary objective, CLADE is designed to prospectively test if routine use of VL monitoring decreases subsequent viral failure, which has not been done within the Emergency Plan roll-out in Africa in the clinic setting under routine, standard of care conditions. Additionally, CLADE will evaluate the cost-effectiveness of routine VL monitoring in addition to CD4 and clinical monitoring in clinic-based ART management relative to using CD4 and clinical monitoring alone.

2. Inclusion and Exclusion Criteria

A. Inclusion Criteria

1. Male or female $\geq$ 18 years of age at time of consent
2. Identified by clinic staff as intending to start ART at the next clinic visit
3. Provision of informed consent in either Kiswahili, Luo, or English

B. Exclusion Criteria

1. Any reason (medical, physical location of home relative to clinic, or other) existing that the ART team or study team feel will prohibit the volunteer from coming for routine ART clinic visits (to be noted on inclusion/exclusion CRF)
2. Any reason (medical, social, or other) existing that the ART team or study team feel may present a risk to the participant that outweighs the benefit of participating in the study.
3. Pregnancy (confirmed or suspected) at time of enrollment.

3. ENDPOINTS

A. Primary Endpoint

1. The study primary endpoint is viral failure at the 18 month follow-up visit as defined by VL >1000 copies/ml by a viral load platform approved in the KEMRI/CRC Laboratory under the College of American Pathologist (CAP) and related external quality assurance programs.
2. Relative cost-effectiveness of routine VL monitoring in addition to CD4 and clinical monitoring compared to CD4 and clinical alone in clinic-based ART management.

B. Secondary Endpoints

1. Combined clinical outcome as defined by 2 new WHO stage III events or 1 new WHO stage IV event
2. Death (as confirmed by medical records or death certificate, a reportable event captured in data collection to be reported to IRBs)
3. Hospitalization (for any illness, a reportable event captured in data collection to be reported to IRBs)
4. Opportunistic infections (onset after study entry)
5. Adherence (as routinely captured by clinics)
6. Lost to follow-up (defined as missing 2 consecutive appointments and inability to have final study visit)
7. HIV genotypic resistance in all treatment failures and all viral failures at the final study visit
8. Feasibility of rolling out viral load monitoring in rural district-level ART clinics (based upon knowledge, attitudes, and performance indicators)
9. Costs of viral load monitoring (in addition to CD4 and clinical care monitoring as actual costs collected during 18 months of follow-up on ART)

10. Adherence to GCP (as an observational research study is incorporated in to ART clinics and based upon GCP indicators)
11. Proportion of participants with virological failures using cut-offs of > 400, > 1000, and > 5000 copies/ml.
12. HIVDR Early Warning Indicators (EWI)

4. RANDOMIZATION AND STRATIFICATION

Randomization (1:1) codes to either Routine Care (Arm A) or Viral Load Guided Care (Arm B) will be generated in blocks of 20 for each site with each site having 50% of patients enrolling being assigned to each arm. The randomization codes will be computer generated by the KEMRI/WRP CRC Information Technology (IT) Department. After informed consent has been signed and the inclusion/exclusion criteria have been met, the Site Coordinator at each site will call the KEMR/WRP IT Department Manager or Assistant Manager for study arm assignment. After the study Site Coordinator provides the IT Department with the patient's clinic number, the IT Department will inform the site Study Coordinator with the study arm assignment and random 4-digit subject identification number (SID) (See Section VII, Data Management). Both the randomization arm and SID will be recorded on a randomization CRF.

For analyses, participants will be stratified by arm using their screening CD4+ cell count as follows: <50 or ≥50 cells/mm^3. This stratification will occur in order to consider the the impact of advanced HIV/AIDS upon the primary outcome of viral load.

5. SAMPLE SIZE

Since CLADE is an observational study evaluating 2 recommended laboratory diagnostic approaches in monitoring ART, viral failure will be the primary endpoint for analyses. In addition, AIDS morbidities as a combined clinical outcome will be analyzed as a secondary endpoint. Below are sample size calculations for the primary endpoint as well as power calculations for a combined clinical endpoint.

For the primary endpoint analyses, a total of 734 subjects (367 per arm) are required in order to achieve 80% power (alpha=5%) and a two-sided z-test with continuity correction to test the null hypothesis. This sample size will allow performance of 1 interim analysis that will be conducted when half of participants have had 18 months viral load (final study visit, week 72, visit 10) and a final analysis at the end of the study using the O'Brien-Fleming spending function. The lower and upper boundaries for the interim analysis will be z (lower) = -2.9626 and z (upper) = 2.9626, with a p-value = 0.0031. For the final analysis the boundaries will be z (lower) = -1.9686 and z (upper) = 1.9686, with a p-value = 0.0495. Two inflations to the sample size have been made. Based upon experiences in the study regions, a liberal estimate of 5% will be lost to follow-up. Additionally in consideration of censoring in Arm-A (see "Censoring Analyses below"), an additional 5% will be added to the sample size. This 10% inflation results in an overall study sample size of 818. A total of 820 participants (410/arm) will be enrolled in CLADE.

The following assumptions and parameters were used in sample size calculations (done by PASS 2008, Dec. 3, 2007).

<u>Null hypothesis:</u> *p*VFrc = *p*VFvc
Probability of viral failure/routine care = probability viral failure/viral load care

1. Probability viral failure via routine care (*p* VFrc) = 12%
   (based upon early screening of viral failures in Kericho District)
2. Probability viral failure via viral load-guided care (*p* VFvc) = 1%
   (based upon experience in Phase III therapeutics study in Kericho)

Estimated "most conservative": delta = 5%; *p*VFrc = 8.0% & *p*VFvc = 3.0%

<u>Sample Size Given Varying Failure Probabilities*</u>

Parameters: Binomial distribution; two-samples; two-sided with continuity correction; Power=0.80; significance level (alpha)=0.05; sample allocation ratio=1; 1 interim analysis and 1 final analysis.

In determining the final study sample size, ranges around the above estimates and resulting sample sizes were explored as noted below in Table 8A.

Table 8A. Sample Sizes Based Upon Varying Viral Failure Rates

		***p* VFrc (probability (%) of failure on routine care arm)**				
		**6.0**	**8.0**	**10.0**	**12.0**	**16.0**
***p* VFvc (probability (%) of failure on viral load arm)**	**0.1**	167 (5.9%)	123 (7.9%)	97 (9.0%)	80 (11.9%)	59 (15.9%)
	**0.5**	199 (5.5%)	140 (7.5%)	97 (9.9%)	87 (11.5%)	62 (15.5%)
	**1.0**	252 (5.0%)	166 (7.0%)	123 (9.0)	**97 (11.0%)**	68 (15.0%)
	**3.0**	818 (3.0%)	**367 (5.0%)**	224 (7.0%)	157 (9.0%)	95 (13.0%)
	**5.0**	8,389 (1.0%)	1,130 (3.0%)	477 (5.0%)	278 (7.0%)	140 (11.0%)

*Key per cell: n, per arm; and, delta in %

Based upon initial assumptions resulting in a delta of 11%, a total of 194 patients would be needed. Given variability in these estimates and assumptions, more conservative delta (5%) was accepted resulting in a total of 734 subjects (367 per arm) being required.

After adjusting for two inflations (10%), 820 subjects (410 per arm) will be enrolled in this study.   The study is well powered to test the primary hypothesis.

Given ART reduces HIV morbidity and CLADE aims to compare two diagnostic approaches used to monitor ART, a combined clinical outcome of AIDS progression defined as 2 new WHO stage III events or 1 new stage IV event will be evaluated as a secondary outcome/analyses.   Power calculations are described below in comparing possible differences in the combined clinical outcome.

Modified from an ongoing Adult AIDS Clinical Trials Group therapeutics study (A5221), the tables below show the power of CLADE to detect differences in the combined clinical outcome. Given likely variability in the secondary endpoint/outcome and aim to assure a clinical outcome can supplement the primary endpoint/outcome, power calculations are based upon a binomial secondary endpoint with a conservative sample size of 360/arm (noting the primary CLADE sample size is 367/arm).

The impact of an assumed difference on the rate of AIDS progression in comparing VL monitoring to routine care monitoring is shown in the left part of the table, while the power to detect the difference is shown in the right part of the table. Results are provided for the VL arm being better than the routine care arm (Table 8B) and for the VL arm being worse than the routine care arm (Table 8C). For example, assume an effect size of 30% (the second column of effect sizes) and a rate in the conventional arm of 35% (the forth row). If VL monitoring is better than routine care monitoring, then the combined clinical outcome rate in the VL arm would be only 24.5% compared to 35% in the routine care arm, and there is 86% power to detect this difference. If VL monitoring is worse than routine monitoring, then the combined clinical outcome rate in the VL arm would be 45.5% compared to 35% in the routine care arm, and there is 80% power to detect this difference. These results are underlined in Tables 8B and 8C.

Table 8B and 8C. Power Estimates in Comparing VL-Monitoring (Arm-B) vs. Routine Monitoring (Arm-A) as Rate of Combined Clinical Endpoint and Difference Between Groups Varies

*Table 8B. VL Arm Sees Reduction in Combined Clinical Endpoint * §*

Arm-A Rate of Combined Clinical Endpoint	Assumed Reduction in Baseline Rate				Assumed Reduction in Baseline Rate			
	20%	30%	40%	50%	20%	30%	40%	50%
	Rate of Combined Endpoint				Power to Detect Difference			
20%	16%	14%	12%	10%	27%	55%	82%	96%
25%	20%	17.5%	15%	12.5%	34%	67%	91%	99%
30%	24%	21%	18%	15%	42%	78%	96%	99%
35%	28%	24.5%	21%	17.5%	50%	86%	98%	99%
40%	32%	28%	24%	20%	59%	92%	99%	99%

*Table 8C. VL Arm Sees Increase in Combined Clinical Endpoint * §*

Arm-A Rate of Combined Clinical Endpoint	Assumed Increase in Baseline Rate				Assumed Increase in Baseline Rate			
	20%	30%	40%	50%	20%	30%	40%	50%
	Rate of Combined Endpoint				Power to Detect Difference			
20%	24%	26%	28%	30%	23%	46%	69%	86%
25%	30%	32.5%	35%	37.5%	30%	58%	82%	94%
30%	36%	39%	42%	45%	38%	70%	91%	98%
35%	42%	45.5%	49%	52.5%	47%	80%	96%	99%
40%	48%	52%	56%	60%	56%	89%	98%	99%

* Calculations used $\alpha=0.045$ to allow for interim analyses

§ Combined Clinical Endpoint = AIDS progression defined as 2 new WHO Stage III events or 1 new WHO Stage IV event

These estimates show that we have reasonable (82%) to excellent power (99%) to detect a 40% reduction in the combined clinical endpoint for a rate of 20% or higher in the routine monitoring (Arm-A) arm. Similarly, we would reasonable (78%) to excellent power (92%) to detect a 30% reduction in the combined endpoint if the rate in the routine monitoring (Arm-A) arm is 30% or higher. For increases in the combined endpoint, we have reasonable (82%) to excellent power (98%) to detect a 40% increase, if the rate of the combined endpoint in the conventional arm is 25% or higher and reasonable power (80% to 89%) to detect a 30% increase if the rate of the combined endpoint in the conventional arm is 35% or higher.

Several secondary objective analyses will be conducted within subsets of CLADE (e.g. comparing agreement between CD4 and retrospective VLs in Arm-A, secondary objective #3). Table 8D below describes a range of sample sizes of subsets (assuming equal sample sizes in groups for two-sample tests).

In column 2, the largest difference between two proportions is presented that would need to be true for there to be a minimum of 80% power (2-sided $\alpha = 0.05$). The power is lowest when the proportions are near 0.50. If the true proportions are closer to 0 or 1.00, there will be higher power to detect the same absolute difference. For example, with 200 participants per group for a secondary analysis, we would have 80% power to detect a statistically significant difference if the true rates were .427 and .573 in the two groups.

In column 3, the difference between the means of two normally distributed samples (e.g., square root of CD4) that can be detected with 80% power (2-sided $\alpha = 0.05$). The difference is expressed as a proportion of the common standard deviation of the variable.

In column 4, the widest width of a 95% confidence interval (CI) around an observed proportion for the given sample size in one group. This width is widest at an observed proportion of 0.50. If observed proportions are closer to 0 or 1.00, the width is narrower for the same sample size. For example, for 300 participants, the 95% confidence interval around an observed proportion of 0.50 is 0.441-0.559, so the width is 0.118.

Table 8D. Detectable Differences for Comparisons Between Groups or Accuracy of Estimates Within a Group

N per group	2-sample binomial	2-sample normal	1-sample binomial
400	[.449,.551]	.203	0.102
300	[.441,.559]	.234	0.118
250	[.435,.565]	.257	0.128
200	[.427,.573]	.288	0.144
150	[.416,.584]	.331	0.166
100	[.396,.604]	.409	0.204

CLADE will have greater than or equal to reasonable power ($\geq$ 80%) for secondary analyses between the two arms (n approximately 400/arm) or analyses within an arm (n approximately 200). As routine, power will drop for additional sub-analyses with lower numbers of participants.

Based upon recommendations from the O-GAC Care and Treatment PHE Technical Team, 200 participants randomly selected at baseline and all persons with viral failure at the final study visit will have HIV genotypic resistance testing. Sample size calculations provided by the Care and Treatment PHE Technical Team are provided in Appendix VI.

6. ACCRUAL AND REPLACEMENT OF STUDY PARTICIPANTS

Full study enrollment (n=820) is anticipated to take approximately 1 year based upon estimates of routine enrollment (see Section X, Study Sites) as demonstrated below (Table 9), although it is possible accrual may occur more rapidly.

Table 9. Estimated Study Accrual Time in Months per Site

Site	Allocation	ART Uptake/mo	50% Study Participation	Months For Full Accrual
Kericho	150	45	22.5	6.2
Kisumu West	190	40	20.0	9.5
Kapkatet	90	16	8.0	10.0
AIC Litein	90	23	11.5	7.0
Nandi North	110	23	11.5	8.7
Nandi South	90	20	10.0	8.0
Tenwek	100	20	10	9.0

As demonstrated above, using conservative rates of 50% of patients starting ART participating in CLADE per site, full study accrual could occur in as early as 6 months with an average overall of 7.8 months. The range across all sites is 6-10 months. Given accrual at the beginning is anticipated to be slower, a conservative estimate of 12 months for full accrual is considered. Study accrual will be tracked closely by site per month. In consultation with the MOH based upon accrual trends, the protocol team may reallocate up to 30% of the allocated enrollment quotas after every 3 months of study duration. Study accrual will be reviewed by the Data Monitoring Committee (DMC; Section XVII, Data Monitoring Committee) every 6 months.

Total follow-up per participant will be 18 months after initiation of ART. The overall study follow-up will be approximately 1,230 patient-years (18 months x 1 year/12 months x 820 participants = 1,230 patient-years).

Patients withdrawing from CLADE or lost to follow-up will not be replaced.

7. Analyses

A. Primary and Secondary Endpoints

For the primary endpoint (viral failure), the log rank Chi-square or Fisher's exact test will be used to compare proportions of viral failures in each arm. Primary endpoint analyses will be conducted on participants with final study visit viral loads at 18 months follow-up. Persons who have died or are lost to follow-up will not be included in primary endpoint analyses. Accordingly, the primary per-protocol analysis will be limited to those participants completing the final study visit within the protocol defined, +/- 21-day study window (see Table 10). A secondary, intent-to-treat analysis will also include all participants completing the study with evaluable VL data (i.e. those out of window but who received a VL) in light of more recent targeted VL monitoring guidelines (50, 51).

Additional viral and treatment failure analyses will be conducted. Failure curves will be estimated using the Kaplan-Meier product limit method and compared between arms using the Mantel (log-rank) test. Hazard rate ratios (HRs) derived from Cox proportional hazards regression analyses will be used to estimate the risk of failure between arms. Multivariable analysis will be performed to determine the potential association between viral failure status and socio-demographic and behavioral factors. One-way interaction terms will be introduced to assess effect modification (including clinic site), and the proportional risk assumption will be tested. To consider impact of baseline disease, baseline CD4 will be evaluated as a categorical variable (less than or equal to/greater than 50 cells/mm^3). The magnitude of the association of predictors for viral failure defined by VL > 1000 copies/ml will be expressed as odd ratios (OR) with 95% confidence intervals. Univariate and multivariate logistic regression analysis for correlated data (random effects and generalized estimation equations) will be used to estimate OR and adjusted odds ratios (AOR) respectively.

For the primary endpoint, four end-point analyses (one per-protocol and two "intent to treat") will be conducted with regard to censoring subjects on Arm-A. First, a per-protocol end-point analysis will be conducted without censored subjects. Second, an end-point analysis will be conducted including those subjects who did have viral failure. Third, an analysis will be conducted including those subjects who did not have viral failure. Finally, an analysis will be conducted excluding any participant on Arm A who

received a VL at any time (i.e. the subset who only had clinical and CD4 monitoring). Results will be presented for each analyses. Such analyses will present the opportunity to evaluate the impact of censoring.

The magnitude of such censoring and potential biases imparted can be considered a-priori. In four years of treatment in this region, less than 1.5% of patients are on second line ART. After 1 year of treatment, less than 0.8% of patients are on second line. Based upon this experience in the region, it may be estimated that 3-6 persons may be evaluated for treatment failure in Arm-A and receive a VL. By current sample size estimates prior to inflation for censoring and interim analyses, it may be estimated that 30 viral failures will occur in Arm-A and 11 viral failures in Arm-B.

The magnitude of censoring may result in up to 6 persons being excluded from end-point analyses. If all are viral failures (the likely scenario), then the result may be a lower proportion of viral failures (end points) in Arm A (30-6=24; or 6.3%, new delta of 3.3%). This may result in a bias toward the null. On the other hand, if the VLs are found to be suggestive of viral suppression, then the failure rate may be artificially high (i.e. X number Arm-A failures / 367 – censored). This may result in incorrectly rejecting the null hypothesis.

Given the overall designation of treatment failure and switch to second line remains low (3% in Kenya as presented at the June 2008 National ART Stakeholders meeting, which is high compared to the study region), adjustment in sample size is made in effort to mitigate potential bias or limitation in power by censoring.

Two retrospective analyses are intended on stored samples. First, retrospective VLs will be conducted on all persons in Arm A with 6-month CD4 counts to assess the degree of agreement between clinical and immunological criteria for treatment failure and concurrent VL. Based upon clinical management, patients will be categorized as having clinical treatment failure or not. The retrospective, concurrent VL will be evaluated for agreement (categorized as viral failure [> 1,000 copies/ml] or not). McNemar and Kappa

statistics will be used for analyses to express degree of agreement. Second, retrospective VLs will be run on stored samples for the 3 month visit in both study arms (week 12, visit 5) in order to determine proportions of study participants starting ART who have viral suppression defined as a VL $\leq$ 1000 copies/ml or a 1-log drop from baseline VL. Of note, these 3 month and Arm-A concurrent VL analyses are conducted retrospectively since such testing is currently not included as routine in MOH guidelines. Such data will be informative in future MOH considerations about VL testing.

For HIV genotypic resistance testing, routine descriptive statistics will be used to describe proportions of viral failures overall and by drug class (e.g. NRTI, NNRTI, PI). For determining the baseline sample of 200 for genotypic resistance testing, only participants with a baseline VL>1000 copies/ml will be eligible for genotypic resistance testing consistent with HHS guidelines for genotypic resistance testing (12). Of those eligible, a random sample of 200 will be selected for genotypic resistance testing. For reporting proportion of genotypic resistance, the numerator will be those with HIV genotypic resistance to any class of ART. The denominator will be 200 (representing the random sample). Accepted International AIDS Society-USA (IAS-USA) drug resistance mutation figures will be used in description of HIV genotypic testing resistance findings (67, 68). Finally, persons with viral failure suggestive of treatment failure will be analyzed to compare VL with resistance testing at time of treatment failure.

Two specific secondary endpoint analyses will be conducted for evaluation of feasibility of introducing routine VL monitoring in to ART clinics and adherence to GCP. Metrics for feasibility will involve two quantitative and 1 qualitative criteria. A VL Knowledge, Attitudes, and Performance (KAP) survey has been created. This questionnaire contains quantitative (true/false, multiple choice) questions regarding use of VL. Qualitative questions regarding attitudes for VL use are also included. This questionnaire will be administered during early PEPFAR education about VL monitoring (under routine ART program implementation) and at 12 months (+/- 1 month) after initial randomization. In addition, a VL Performance Indicator CRF has been created to track logistics around VL

monitoring (e.g. ordered on time, received at lab appropriately, laboratory analyses conducted appropriately, results returned to chart and patient).

A-priori definition of “feasible” with regard to routine use of VL monitoring in ART clinics will include reaching each of the following milestones: A. an overall 80% correct response on the knowledge portion of the KAP questionnaire at year-1; B. a significant ($p<0.05$) overall improvement in scores between baseline and year-1 (paired students t-test); and, C. 80% of VL CRF indicators adhered to during the first 12 months of the study. Adherence to GCP will be evaluated based upon GCP indicators that have been created. For final determination of GCP adherence, three categories are defined: A. perfect adherence (100%); B. strongly adherent (80-99%); and needing improvement (<80%).

For additional secondary analyses (e.g. comparisons of WHO stage clinical progression), the Chi-square or Fisher’s exact test will used to compare categorical variables. For continuous variables, the Student-*t* test or the Mann-Whitney U test will be applied. For secondary objectives and endpoints, analyses will be conducted overall (after randomization) and after the first week-24/6-month evaluations (after first scheduled routine viral load).

At the request of the independent Data Monitoring Committee, additional information will be ascertained for secondary analyses regarding cause of death (secondary hypothesis #10 and related secondary objective #12, secondary endpoint #2). Currently, all-cause mortality (number overall and by study arm) is captured and presented along with the source of death report (e.g. report from clinic staff, confirmed burial). To present additional detail, death reports previously submitted to the IRBs will be reviewed for assigning cause(s) of death on using a dedicated CRF. The CRF will include only the subject identification number and dates of death and IRB report so the data may be merged with the existing data noting overall death. This will allow additional information regarding the cause and reported location of death to be added to the database along with an assigned degree of validity/certainty based upon information

available in the death report. This analysis will be limited to the IRB death reports with no additional contact or information sought.

For all analyses, all p-values will be two-sided. All statistical analyses will be performed in Kenya at the KEMRI/WRP CRC Information Technology Department using STATA version 9 (Stata Corporation, College Station, TX) and/or PC SAS (SAS Institute, Carey, NC).   Data analyses are anticipated to continue for 2 years after the last participant has completed the final study visit.

B. Cost Effectiveness Analyses

A cost-effectiveness analysis of the two clinical care strategies (Routine Care/Arm A and Viral Load Guided Care/Arm B) will also be completed as part of this study. There is a vast literature on the use of cost-effectiveness analysis (CEA) in healthcare decision making, in both developed and developing countries. Relevant CEA methods are thoroughly described in standard textbooks (65, 66) and recent papers provide many examples and references (34, 35, 36). The WRP/KEMRI/BU collaboration is already conducting a cost-outcomes analysis in three KEMRI/WRP-supported treatment sites in the general study region (39). The cost-effectiveness analysis for this study will use the basic costing methodology already in place as part of the on-going “Cost and Outcomes of models for delivering antiretroviral therapy for HIV/AIDS in Kenya” protocol underway as part of the KEMRI/BU/WRP research program, which has also been successfully used in several other studies completed by BU in South Africa and elsewhere (see Rosen et al, 36). Costing information to be extracted from charts is provided in Appendix VII.

CEA is an especially valuable source of information for evaluating clinical care strategies when the outcomes to be evaluated are identical, as is the case with this study. For the CLADE study, the primary outcome, virologic failure, is defined as a confirmed plasma HIV-1 RNA level that is >400 copies/mL. For the cost-effectiveness analysis, however, we will use virologic success defined as a confirmed plasma HIV-1 RNA level that is

$\leq$400 copies/mL. Some patients may die or be lost for other reasons and not complete the full study period. These patients will be defined as not-in-care at the end of the study period. The basic criterion is to evaluate the average cost per treatment success for the two study arms. With n patients at a site, each with a treatment cost of c_i , i = 1, ….n, and a treatment outcome, y_i = 1 if success, else 0, this cost-effectiveness criteria can be written as:

$$ce^k = \sum_{i=1}^{n} c_i^k / \sum_{i}^{n} y_i^k = c_{mean}^k / p^k$$

where $c_{mean}^k = \Sigma_i c_i^k/n$ is the simple average of individual costs c_i^k and $p^k = \Sigma_i y_i^k$ is the proportion of successes out of the total (all patients completing the study period and suppressed viral load), where k = cp represents current practice and k = vl represents viral-load guided care. The basic hypothesis of the CLADE study is that $p^{vl} \geq p^{cp}$, although this hypothesis will be tested as part of the study.

The proportion of patient successes is provided directly by the study data. The costing methodology used by previous Boston University Center for International Health and Development (CHID) cost/outcomes studies will be used to document resources and per unit costs to estimate per patient costs of all resources used as part the study-period care and treatment program.

For this study, the cost-effectiveness analysis is based on data extracted on all resources used for all patient encounters during the 72-week study period. Clinical record data are used to identify the quantities of resources used during each patient encounter at the site during the study period. The CHID and Wits Health Consortium, Health Economics Research Office (HERO) costing database created in CSPro (a free software available through the US Census Bureau) provides the basic structure for inputting all information from patient records. For each outpatient visit by a study subject during the period from medical eligibility for ART to 18 months after initiation of ART, we will extract from existing medical records for as many of the following fields as are available: age, sex, height, visit date, primary reason for visit, types of professionals seen, group sessions attended, CD4 count, viral load, weight, opportunistic infections or other AIDS- or ART-

related conditions, other AIDS- or ART-related lab tests and x-rays performed, medications prescribed and provided or not provided by site pharmacy, patient fees paid, relevant care received from other providers, admissions for and details of inpatient care since previous visit.

Once all primary information for all patient visits are inputted into the CSPro database, the CIHD/HERO, a SAS program is used to aggregate information from all patients visits into a summary data base on the quantities of key resources used during the study period for each patients. The key resources include the number of patient encounters with various staff (e.g., doctors, medical officers, nurses, counsellors, etc.), pharmacy visits, all tests included viral load tests, units of all medications prescribed, and so on. The results of this SAS analysis are outputted to an excel spreadsheet file, that is then used as an input file in the CIHD/HERO "costing model lite" that was created previously by the CIHD/HERO team and is being used as part of existing cost-outcomes studies implemented by the KERMI/BU/WRP collaboration. Experience with this model shows that adjustments are needed to allow for the specific circumstances of facilities and treatment protocols across countries. One of the original authors of these costing databases and models is Mr. Lawrence Long from the CIHD and HERO. He is a co-investigator on the existing cost-outcomes work completed by the KEMRI/BU/WRP collaboration, and he will be available to the study team to facilitate any adjustments to the model needed to complete the analysis.

Clinical record data will not be extracted for any subject before 18 months after that subject initiated ART, so that all data pertaining to a single subject can be collected during a single access to the CLADE study folder.

Besides information on all quantities of resource used for managing each patient are developed, it is necessary to develop financial cost data from the site to develop unit costs of resources used to complete the costing analysis. For these variable costs, we will collect information on amounts paid for relevant inputs at the most recent date of procurement. Unit costs of drugs, diagnostics, lab charges, and other variable inputs will

be recorded from existing invoices. Information on the purchase, maintenance, and operating costs of infrastructure and equipment will be taken from financial reports or, if necessary, from data on relevant market prices (e.g. for vehicles). Personnel costs will be collected from each site's payroll records. These data will not pertain to individual subjects or employees. They will be obtained through interviews with site managers and review of documents and facility-level records.

A similar process is used to develop estimates of facility level annual fixed costs, which are then apportioned equally to all patients receiving care and treatment at the site to develop an estimate of annual per patient fixed costs. These fixed costs are then added on to the patient's variable costs to estimate total costs per patient. For patients who die or drop out of the study, these annual fixed costs are reduced proportionately by time in the study. For example, if annual (52 week) fixed costs are F and a client remains in the study for 37 weeks out of the total 48 week study period, fixed costs for this patient will be estimated as F(37/48)(48/52). While annual costs per patients are identical for the same site, the two study arms may have differing numbers of patients who do not complete the entire study period, so that fixed costs per patient are not identical for all patients.

Once average costs per study arm estimated for each study patient, we will estimate ce^{cp} and ce^{vl} for each study arm. Because these variables are ratios of random variables, it is not possible to use simple mean difference tests to estimate is these outcomes are significantly different. Bootstrapping methods, which are provided in most statistical problems (e.g. STATA) or can be programmed into Excel, will be used to estimate confidence intervals for each cost-effectiveness outcome for each study arm and test the null hypothesis of no-difference in treatment arms.

XVII. DATA MANAGEMENT

1. DATA STORAGE AND COLLECTION AT STUDY SITES

Each participant entering CLADE will have a study folder kept at the ART treatment site/clinic in a locked filing cabinet with access limited only to study staff. The CLADE study folder consists of case report forms (CRFs) mirroring the participant's clinical record with data extracted as necessary for study analyses. The CLADE study folder will have no personal identifying information. The folder will be identified by the patient clinic "HAART" number (assigned at all clinics sequentially as patients enter clinics) and 4-digit random SIN. A summary of data to be collected on the CRFs is presented in Table 10 below.

Procedurally, extraction of relevant clinic data and population of the CLADE study database will occur in three steps. First, relevant data will be recorded on visit CRFs at the time of routine HIV clinic evaluations. Second, every 2 weeks or at a frequency necessary depending upon site accrual, members of the IT data extraction team will report to each study site to electronically enter all study CRF data onto CLADE study laptops. Three CLADE laptops are planned to covered the study sites and geographic region. The CLADE study laptops will be password protected with all data encrypted. Finally, data will be downloaded to the CLADE study database at the KEMRI/WRP CRC IT Department daily or at a frequency no greater than once weekly (i.e. in the event 2 adjacent sites such as Nandi North and Nandi South are visited in one trip for extraction and the team spends the night away from the CRC IT Department). After data is downloaded to the CLADE Study Database at the KEMRI/WRP CRC IT Department, the data on the CLADE laptop will be erased. No identifying information will be kept on the CLADE laptops. The CLADE laptop will be kept in the KEMRI/WRP CRC IT Department when not being used at sites for data retrieval.

All CLADE data will be managed and analyzed in Kenya at the KEMRI/WRP CRC IT Department. The study database is a relational database management system (RDBMS) with integrated data management and analysis software. The application will be a single entry system with programmed logic checks and prompts. The CLADE database will be stored on a password protected, secured folder in the CRC research servers. Access to the CLADE database will be restricted to the IT Manager, IT Assistant Manager, and data

entry clerks. The CRC IT area is secured with limited access controlled by an electronic door lock. The server room is temperature controlled with sensor phones for alert of any temperature deviations. All research servers are backed up daily and a copy of the backup saved securely in a fireproof safe at an offsite location. The CRC IT Department is within the larger CRC complex approved by the US Embassy Regional Security Officer (RSO), guarded 24 hours a day, and protected by a surrounding concrete fence.

Table 10. Routine Ministry of Health Data to be Collected on Case Report Forms and in the Study Database[1]*

	Pre-ART[11] [v-1]	Study Entry[12] (Week 0) [v1]	Week 2 [v2]	Week 4 [v3]	Week 8 [v4]	Week 12 [v5]	Week 24 [v6]	Week 36 [v7]	Week 48 [v8]	Week 60 [v9]	Week 72[13] [v10]
HIV clinic registration	X										
HIV documentation	X										
Counseling & education	X	X	X	X	X	X	X	X	X	X	X
Baseline medical history[2]	X										
Physical exam[2]	X	X	X	X	X	X	X	X	X	X	X
Clinical assessment[3]	X	X	X	X	X	X	X	X	X	X	X
Adherence ed. & assessment	X	X	X	X	X	X	X	X	X	X	X
OI diagnostic tests	As clinically indicated										
Laboratory Chemistries[4]	X			X			X		X		X
Laboratory CBC[5]	X			X			X		X		X
Urinalysis		X		X		X	X		X		X
Pregnancy test[6]	As clinically indicated										
CD4 Count	X						X		X		X
Chest X-ray	X	As clinically indicated									
CLADE education[7]	X	X									
Informed consent form signing	X										
Sputum for AFB	As clinically indicated										
Inclusion/Exclusion criteria		X									
Informed consent confirmed[8]		X									
Enrollment form confirmed		X									
Randomization		X									
Viral Load (Arm B)		X					X		X		X
HIV resistant testing[9]		X		As clinically indicated							
Determination of secondary endpoints[10]		At each clinical encounter									

Notes:

1. All data extracted are consistent with standard of care as recognized by the Kenya MOH. For purposes of data extraction, visits during the first two months of ART (v2-4) will be considered +/- 1 week of the calendar date as would be routine. All subsequent visits (v5-10) will be +/- 3 weeks of the calendar date. Such windows are necessary to accommodate for routine clinic and patients preferences in scheduling routine ART follow-up. The table notes tests or activities consistent with MoH guidelines and not necessarily data that must be collected for this observational study given some variation exists at different clinics and with different clinicians.
2. Data from the clinic's baseline medical examination/encounter form to be extracted will include sociodemographic information, significant past medical history including medicines, and WHO staging. Only information on the routine clinic encounter form will be extracted. No identifying information (i.e. name, physical address) will be extracted.
3. Data from routine clinic follow-up forms for clinical evaluations (e.g. adherence, vitals, weight, WHO stage, pertinent physical examination findings, new or changes in medications, and referrals) will be extracted for each visit. Only information on the routine clinic encounter form will be extracted. No identifying information (i.e. name, physical address) will be extracted.
4. Laboratory chemistries are conducted consistent with MOH guidelines and as clinically indicated. The extent of chemistries ordered is at the discretion of the clinician. Only data from chemistries conducted will be collected. In addition, alanine aminotransferase (ALT) is recommended at weeks 2, 4, 8, and 12 for patients receiving nevirapine.
5. Laboratory complete blood counts are conducted consistent with MOH guidelines and as clinically indicated. The extent of complete blood counts ordered is at the discretion of the clinician. Only data from complete blood counts will be collected. In addition, hemoglobin is recommended at weeks 4 and 12 for patients receiving nevirapine.
6. A pregnancy test is recommended for women who may start efavirenz therapy.
7. Introduction to the CLADE study will occur at the first clinical encounter as part of the pre-ART work-up. An IRB-approved handout regarding CLADE as well as the informed consent document will be provided to interested clinic patients.
8. Data from pre-ART (visit 1) will be extracted only after informed consent has been signed.
9. HIV genotypic resistance testing will be conducted on all treatment failures prior to second line therapy initiation and on a random sample of 200 study participants initiating ART who have a baseline VL>1000 copies/ml.
10. Secondary endpoint data to be extracted from follow-up records include: WHO staging, death, hospitalization, opportunistic infections, and adherence, and loss to follow-up.
11. Pre-ART represents retrospective data collection after consent has been obtained at study entry.
12. Once a determination has been made by the ART clinic that a patient will start ART, the patient will be asked about interest in participating in CLADE. If so and informed consent is obtained and the patient meets study inclusion/exclusion criteria, the enrollment will proceed. Those randomized to Arm-B (VL-guided care) will have VL drawn prior to starting ART.
13. The final study visit where data will be extracted is visit 10 (approximately 1 ½ years on ART following MOH guidelines).

2. Data Ownership and Storage After Study Completion

Data for this study will be jointly owned by the Kenya Medical Research Institute and Walter Reed Army Institute of Research. After completion of study-related visits, all CLADE study related data including patient study folders with informed consent and CRFs will be kept at the KEMRI/WRP CRC in Kericho following the study for periods of time as required by the Kenya Medical Research Institute and Walter Reed Army Institute of Research. This period of time for the CLADE study will be 6 years based upon WRAIR Policy Letter (07-13, records management, 27 November 07).

XVIII. DATA MONITORING COMMITTEE

A data monitoring committee (DMC) consisting of Kenyan and international representation has been created for CLADE with detailed operational logistics in the CLADE DMC Terms of Reference document. The CLADE DMC consists of 7 persons with international expert representation covering HIV/AIDS treatment in Kenya, HIV/AIDS clinical research, biostatistics, and human subjects protection/bioethics. In addition, a representative from the Kericho HIV Community Advisory Board will be included. The primary tasks of the DMC are to review interim and final primary and secondary outcome study data as well as other data regarding study conduct (e.g. accrual, metrics of viral load monitoring utility) and make recommendations to the study principal investigators, study sponsor, and regulatory bodies.

After meeting to review the protocol prior to/or during IRB reviews, the DMC will be scheduled to meet every 6 months (+/- 1 month) after first randomization and within 60 days of the final study data set being available for review. However, the frequency of meetings may be altered to a more frequent interval at discretion of the DMC and DMC Chair. Written DMC recommendations will be submitted to the study sponsor and protocol Co-Chairs within 10 working days after DMC meetings. Data to be reviewed by the DMC will include study accrual patterns, cumulative and interim primary and secondary endpoint data, and all treatment failures with or without switch to second line therapy. HIV resistance testing (both population and real time) will

also reviewed by the DMC. Cost-effectiveness data will only be made available to the DMC at the end of the study.

Recommendations regarding study conduct will be largely at the discretion of the DMC and focus upon accrual, safety signals including second line therapy switches, and cumulative primary and secondary endpoints. One a-priori criteria for recommendation to discontinue CLADE is specially noted. One interim primary endpoint analyses of viral failure between the two arms is planned after half of the participants (410) have reached the final 18-month study visit (visit 10; week 72) to determine if a significant difference between the two arms exists. If so, the DMC may recommend termination of the study overall based upon the primary viral failure endpoint, continue the study overall per protocol, or continue components of the study. This is further addressed in the CLADE Data Monitoring Committee Terms of Reference.

For this observational study of MOH ART care, the DMC recommendations would be considered binding for the CLADE study unless determined appropriate otherwise by the study co-Chairs and with agreement of the IRBs and sponsor. Clinical information and findings would be shared with the Ministry of Health for their consideration and determination with regard to how such DMC findings and recommendations may impact clinical care.

XIX. ADVERSE EVENT MONITORING

Given CLADE is an observational study of standard of care based upon MoH guidelines utilizing data extraction without direct patient contact, routine adverse event monitoring and reporting in the context of laboratory abnormalities or therapeutic adverse events will not be conducted. Serious adverse events including death and hospitalization that are identified during data extraction will be flagged by the CLADE database. Such events will be reported to the KEMRI and WRAIR IRBs during continuing reviews and reported to the CLADE DMC for review at the scheduled 6-month interval meetings. In addition, information regarding all observed treatment failures and patients loss to follow-up will be reported in IRB continuing

reports. Finally, any social harm to a CLADE participant discovered or reported to the study team will be reported to all IRBs as an expedited adverse event.
All unanticipated problems involving risk to subjects or others, serious adverse events related to participation in the study and all subject deaths should be promptly reported by phone (301-319-9940) or by facsimile (301-319-9961) to the WRAIR Human Use Review Committee. A complete written report should follow the initial notification. In addition to the methods above, the complete report will be sent to the Director, Division of Human Subjects Protection (DHSP), Walter Reed Army Institute of Research, 503 Robert Grant Ave., Silver Spring, MD 20910-7500. Similarly, this report will be submitted simultaneously to the KEMRI IRB (Section XVIII.12).

Any unanticipated problems involving risk to subjects or others, serious adverse events related to participation in the study and all volunteer deaths, will be promptly reported by phone (301-319-9940), by email ("DHSP WRAIR-Wash DC" <WRAIRDHSP@amedd.army.mil>) or by facsimile (301-319-9961) to the WRAIR IRB. A complete written report should follow the initial telephone call within 10 working days. The WRAIR IRB will forward any reported unanticipated problems or serious adverse events to the U.S. Army Research and Materiel Command's Human Subject Research Board.

XX. HUMAN SUBJECTS PROTECTION & ADDITIONAL ETHICAL CONSIDERATIONS

1. INSTITUTIONAL REVIEW BOARD/INSTITUTIONAL RESEARCH ETHICS COMMITTEES

This study will fall under the auspices of both local and international Institutional Review Boards/Institutional Research Ethics Committees. Kenya Medical Research Institute (FWA #000 02066; expires March 13, 2011) will serve as the local IRB. In addition, the Walter Reed Army Institute of Research Division of Human Subjects Protection (FWA #000 00015; expires November 7, 2009) will have oversight. The CLADE protocol will undergo review at both institutions with approval prior to commencement.

2. INFORMED CONSENT

All participants in CLADE will provide informed consent by signature or mark/finger print (for illiterate subjects as outlined in KEMRI/WRP Standard Operating Procedures for consenting illiterate subjects) prior to study entry. The informed consent document will be reviewed by the appropriate KEMRI and WRAIR regulatory/ethical research committees. Informed consent forms will be available in Kiswahili, Luo, and English. Research in the larger Kericho/Rift Valley Province region routinely uses Kiswahili and English consent forms. Given research in the Kisumu region uses these but also routinely Luo consent forms, Luo informed consent forms will also be available. Additional informed consents may be added based upon local uptake experiences at each site. If any site feels an additional informed consent in an different language is needed, it will be developed and approved by the relevant IRBs prior to use.
No study procedures will be conducted prior to informed consent.

3. RECRUITMENT

CLADE recruitment will occur primarily by the word of mouth and supplemented by a study summary handout and the informed consent form. Clinic staff will be asked to ask patients eligible for ART if they would like to learn about CLADE. For those interested, a 1-page, IRB approved study summary handout will be provided as well as the IRB approved informed consent form. Open education sessions as well as informed consent sessions will be offered to interested persons. Patients who express interest to participate will proceed to formal consenting by the study nurse.

4. POTENTIAL STUDY RISKS AND RISK MANAGEMENT

The primary risks to study participants are potential social harm from study participation, very minimal risks of physical harm from additional phlebotomy for study participation, and a small risk of clinical data being lost or exposed in transporting data from the treatment sites to the KEMRI/WRP CRC IT Department. Given both the drugs (ART) and laboratory procedures (e.g. CD4. viral load) are recommended by the Kenya MOH and are considered part of standard of clinical care

in Kenya, CLADE itself does not impart any risks related to ART or laboratory monitoring.

Efforts are in place to mitigate any study related risks. All study sites have had ongoing ART for at least 3 years to date. All sites have identified HIV clinics, and stigma and potential for social harm while real are far less than pre-ART or in the earlier days of opening HIV clinics. Risks for physical harm from phlebotomy are present to all patients being monitored for ART regardless of study participation. Additional phlebotomy for CLADE is minimal: approximately 5 mls for HIV genotypic resistance testing at baseline and in the event of confirmed treatment failure; 5 mls at the time of CD4 (baseline and every 6 months thereafter) for persons in Arm A (Routine Care) for retrospective analyses to evaluate clinic/CD4 and VL agreement; and, 5 mls at month 3 visit (week 12, visit 5) to evaluate viral suppression at 3 months after starting ART. Patients as part of routine care or study participation can refuse phlebotomy at any time.

Clinical data will be transported from treatment/study sites to the KEMRI/WRP CRC Information Department. There is a small possibility that in doing so, clinical data may be lost or exposed. Mitigating steps include by design no patient identifying information will be extracted from clinical records or transported to the KEMRI/WRP CRC (as outlined in Section X, Data Management). In addition, no data will be stored on the CLADE laptops used to extract and transport data. Such data will be erased upon uploading to the CLADE database in the KEMRI/WRP CRC. Finally, security measures are in place at the KEMRI/WRP CRC and IT Department to assure data safety and confidentiality (as outlined in Section X, Data Management).

5. POTENTIAL STUDY BENEFITS

There may be no direct benefit to study participants. However, potential study benefits may extend to study participants. Use of viral load monitoring may improve clinical outcomes (decrease viral failures). While such benefit is inherent to persons participating in the Viral Load Guided Care (Arm B), persons who are classified as treatment failures in Routine Care (Arm A) will also be offered viral load monitoring. In addition, HIV resistance testing will be offered to all persons with treatment

failures. Therefore, study participants may receive benefit from these tests (VL, resistance testing).

Benefits to those health care providers participating in CLADE sites as well as KEMRI will include a better understanding of the viral load monitoring and evaluations for treatment failures (a secondary study objective). Additional benefit to the clinical and research communities will include preparedness and understanding for viral load monitoring in the context of ART management. Finally, in addition to research findings that may improve ART management in the future, critical epidemiological information regarding HIV genotypic resistance rates for both wild-type HIV as well as in the scenario of treatment failures will be generated.

6. SUBJECT CONFIDENTIALITY

It is the role of the principal investigators to ensure that participant's confidentiality is maintained. In addition to procedures put in place to assure participant confidentiality (see Section X, Data Management), participants will not be identified in any reports on this study. Any copies of source documents or case report forms & data forms bearing any identifiers (e.g. SID) will be kept in locked, secured areas. In respect to electronic records, these shall be held in separate confidential, password-protected databases. Records will be retained for six years after the completion of the study or as directed by the sponsors or the IRB\IEC or as required by local procedures.

Study records including participant files with case report form data and the study database will be made available for review when required by the study sponsor, DoD/WRAIR, USAMRMC, KEMRI, or other authorized individuals as outlined in Kenya and US policies. Direct access includes examining, analyzing, verifying, and reproducing any records and reports that are important to the evaluation of the study. The investigator is obligated to inform the participants that the above named representatives will review their study-related records should need arise and they should consent to such procedures prior to recruitment into the study. Therefore, during the consent process, participants will be informed that the information they provide may be compared to similar information of participants but confidentiality will be strictly maintained.

7. Study Withdrawal

Participation in the study is voluntary. Participants may withdraw from the study anytime for any reason without any loss of benefits. Any person withdrawing from the study will continue to receive ART care and management consistent with Kenya MOH guidelines.

8. Expense to Study Participants and Compensation for Participation

Any undue expense for study participation is avoided. Given the importance of study visits for both data collection as necessary to test study hypotheses as well as general clinical well-being, study participants will be compensated for travel and any additional time that may be imparted by study participation. Compensation for those participants attending routine clinical/study visits will be Ksh 400-500 (approximately $5.00-$7.50). Such compensation is consistent with that approved by the KEMRI for similar study related compensation and will be subject to IRB review and approval. Such compensation will be at the discretion of the participating site in collaboration the site leadership as well as the Ministry of Health or relevant Faith Based Organization oversight where applicable and include consideration such as expenses for transportation to and from the clinic (varies upon clinic location) and time away from labor or work.   This will be reviewed by the KEMRI IRB (and WRAIR IRB).

9. Participant Remuneration

For any additional time added to clinic visits in order to obtain necessary clinical data, participants will receive 400-500 KSH (approximately $5.00-$7.50) for each study defined visit and 150 KSH (approximately 2.30 USD) for each unscheduled visit. This will be at the discretion of the site as outlined above (IX.8).

10. Management of Vulnerable Volunteers

In the event that the status of an enrolled volunteer changes during the course of their enrollment in the study and that the volunteer's ability to exercise free choice could be limited in some way, the volunteer is recognized as a vulnerable participant. A

vulnerable volunteer is any individual whose willingness to volunteer in a clinical trial may be unduly influenced by the expectation, whether justified or not, of benefits associated with participation; or of a retaliatory response from senior members of a hierarchy in case of refusal to participate.

The volunteer that is likely to be vulnerable to coercion or undue influence, might include individuals such as minors, pregnant women, prisoners, soldiers, the physically handicapped, or mentally incompetent persons. Other vulnerable volunteers could include persons in an emergency situation like refugees, persons living on streets, and very sick persons who are incapable of giving consent or providing continuing consent.

If a change in status of a volunteer already enrolled in the study should occur, it is the responsibility of the investigators to assure that appropriate safeguards are in place to protect the rights, safety and welfare of all study subjects. The principal investigator shall notify all Institutional Review Boards (IRB) and/or Ethical Committees (EC) associated with this study in the continuing review report (CRR). The IRB/EC must decide what types of special protections are required and provide direction to the investigator. Short-term medical care for any injury resulting from participation in this research will be provided by the project. The U.S. Federal Government will not provide long-term (over 6 months) medical care or financial compensation for research-related injuries.

11. MODIFICATIONS OF THE PROTOCOL

Amendments to the protocol will be made only after consultation and agreement between the sponsor and protocol Co-Principal Investigators. All protocol modifications (including but not limited to changes in the protocol Principal Investigators, inclusion/exclusion criteria, overall study sample size, modifications in existing or addition of new study objectives and analyses) must be submitted as a written amendment for KEMRI and WRAIR IRB approvals before implementation of the changes.

12. PROTOCOL DEVIATIONS

A protocol deviation is defined as an isolated occurrence involving a procedure that did not follow the study protocol or study specific procedures. The KEMRI ERC and WRAIR IRB will be notified of any deviations/ departures from the protocol that may have an effect on the safety of volunteers and integrity of the study as they occur. Deviations that pose immediate hazards to study subjects will be reported to the WRAIR Division of Human Subject Protection (DHSP) within 48 hours, by telephone or email, upon becoming aware of the event. The PI will then submit a written report to WRAIR DHSP of the deviation within 10 working days. All deviations will be reported in the KEMRI and WRAIR continuing review reports and the final study report. Given CLADE is an observational cohort study of routine standard care, significant protocol deviations resulting in harm to the participant are not anticipated, although monitoring for such will occur with reporting as outlined above.

Non-Emergent/Minor deviations are routine departures that typically involve a volunteer's failure to comply with the protocol. Examples include missing scheduled visits or failing to return diary cards. Minor deviations that occur in minimal risk and greater than minimal risk protocols will be reported to the sponsor (as required) and DHSP/IRB in a summary report with the annual continuing review report. Given CLADE is an observational cohort study of routine standard care, minor deviations resulting from routine clinic missed visits will only be captured via data extraction and not real-time in the clinic.

A cumulative deviation report will be submitted to the DHSP with each protocol continuing review report or with the final report, whichever comes first.

13. STUDY MONITORING

Interim (+/- 4 months from 50% accrual) and final, closeout (within 6 months following final participant completing the final visit) will be conducted by the Division of Retrovirology Regulatory Office Center. Given largely the observational nature of this study, monitoring will focus upon, but not be limited to, informed

consent, patient confidentiality, adverse event reporting (i.e. social harms), regulatory components (e.g. IRB approvals), and DMC and IRB reports.

14. REPORTING OF EXPEDITED/SERIOUS ADVERSE EVENTS AND OTHER REPORTING REQUIREMENTS

Serious Adverse Events to be reported in an expedited manner (as outlined in Section XVII, Adverse Event Monitoring) as well as any unanticipated problems involving risk to volunteers or others should be promptly reported to the U.S. Army Medical Research and Materiel Command's Human Subjects Research Review Board, the Walter Reed Army Institute of Research Division of Human Subjects Protection, and the Kenya Medical Research Institute IRB at the addresses below. Complete written reports should follow the initial notifications.

A. U.S. Army Medical Research and Materiel Command's Human Subjects Research Review Board: phone (301-619-2165), email (hsrrb@amedd.army.mil), facsimile (301-619-7803); mailing address: U.S. Army Medical Research and Materiel Command, ATTN: MCMR-ZB-P, 504 Scott Street, Fort Detrick, Maryland 21702-5012.

B. Walter Reed Army Institute of Research Division of Human Subjects Protection: phone (301-319-9940), facsimile (301-319-9961), mailing address: Director, Division of Human Subjects Protection (WRAIRDHSP@amedd.army.mil), Walter Reed Army Institute of Research, 503 Robert Grant Ave., Silver Spring, MD 20910-7500.

C. Kenya Medical Research Institute: Christine Wasunna (cwasunna@kemri.org) and Caroline Kithinji (ckithinji@kemri.org).

D. Additional reporting requirements specific to USAMRMC ORP HRPO include the following:

<u>Principal Investigator Reporting Requirements to the IRB</u>

The Principal Investigator is responsible for reporting to the WRAIR IRB by phone (301 319-9940), by email (WRAIRDHSP@amedd.army.mil), or by facsimile (301-319-9961) to the Division of Human Subjects Protection, Walter Reed Army Institute of Research, 503 Robert Grant Ave., RM 1W30, Silver Spring, Maryland, 20910-7500.

WRAIR IRB Reporting Requirements for Headquarters Level Review (for Greater Than Minimal Risk Studies or otherwise requested by Headquarters)
The following protocol life-cycle actions reviewed by the WRAIR IRB require prompt (within 10 working days) reporting to the USAMRMC ORP HRPO. The below will be forwarded by the WRAIR IRB to USAMRMC ORP HRPO. These may be reported by email (hsrrb@det.amedd.army.mil), or the complete report can be sent to the U.S. Army Medical Research and Materiel Command, ATTN: MCMR-ZB-PH, 504 Scott Street, Fort Detrick, Maryland 21702-5012.

(1) All unanticipated problems involving risk to subjects or others, serious adverse events related to participation in the study and subject deaths related to participation in the study.

(2) Any deviation to the protocol that may have an adverse effect on the safety or rights of the subjects or the integrity of the study must be reported to the USAMRMC ORP HRPO as soon as the deviation is identified.

(3) Suspensions (to include continuing review lapses), clinical holds (voluntary or involuntary), or terminations of this research by the IRB, the institution, the Sponsor, or regulatory agencies are promptly to the USAMRMC ORP HRPO.

(4) Any modification (amendment) to a research protocol previously reviewed by USAMRMC ORP HRPO that increases risk to subjects or any amendment to a protocol initially approved as minimal risk but now determined by the WRAIR IRB to present greater than minimal risk must be submitted to the USAMRMC ORP HRPO for concurrence prior to implementation. All other

amendments are submitted with the continuing review report to the USAMRMC ORP HRPO for acceptance.

(5) The knowledge of any pending compliance inspection/visit by the FDA, OHRP, or other government agency concerning clinical investigation or research, the issuance of Inspection Reports, FDA Form 483, warning letters or actions taken by any Regulatory Agencies including legal or medical actions and any instances of serious or continuing noncompliance with the regulations or requirements are reported immediately to USAMRMC ORP HRPO.

The following protocol lifecycle actions initially reviewed by the WRAIR IRB require periodic reporting for HQ-level review by the USAMRMC ORP HRPO.

(1) Modifications (amendments) to the research protocol which do not increase risk to subjects must be submitted to the WRAIR IRB for approval. These amendments are submitted with the continuing review report to the USAMRMC ORP HRPO for acceptance.
(2) A copy of the approved continuing review report and the WRAIR IRB approval notification are submitted to the USAMRMC ORP HRPO as soon as these documents become available. A copy of the approved closure study report and local IRB approval notification will be submitted to the USAMRMC ORP HRPO as soon as these documents become available.
(3) WRAIR IRB Monthly Meeting Minutes are sent to USAMRMC ORP HRPO upon receipt of final, official signatures.

The following WRAIR DHSP SOPs are referenced for guidance regarding reporting requirements to the WRAIR IRB: UWZ-C-636, UWZ-C-611.01, and UWZ-C-621.0 and Guidance on Reporting Deviations (dated 17 November 2008) on the website www.wrairdhsp.com.

15. RESEARCH FINDINGS

Research findings in aggregate form will be made available to the study sponsor (Office of the Global AIDS Coordinator), Kenya Ministry of Health (MOH), all participating institutions, and at the requests of regulatory bodies (WRAIR DHSP, KEMRI IRB, BU IRB). Formal presentation of research findings will be made to the Kenya PEPFAR PHE and ART/treatment committees. It is anticipated that study findings will be presented at international meetings and submitted for peer reviewed publication. Appropriate polices and procedures from the WRAIR and KEMRI as well as other appropriate policies will be followed in this process.

16. ROLE OF US MILITARY HIV RESEARCH PROGRAM

The Division of Retrovirology / US MHRP oversees all research and HIV prevention, care, and treatment activities in its international network. In addition to expert technical assistance and support of the CLADE study, MHRP will also provide protocol management as it does for all MHRP research. MHRP's Regulatory Office Center will monitor CLADE.

17. ROLE OF CONSULTANTS

Study consultants participate in the overall protocol concept and design (including planned analyses) and in interpretation of aggregate results. Study consultants will not participate in the actual implementation of the study or have access to any participant data. Dr. Sydney Rosen and Dr. Bruce Larson of Boston University will serve as study consultants.

XXI. RESPONSIBILITIES OF THE PROTOCOL PRINCIPAL INVESTIGATORS

- Promptly report changes or unanticipated problems in a research activity.
- Immediately report, by telephone, any serious or unexpected adverse experiences which occur to the human subject or others as outlined in Section XVIII above.
- To promptly report any change of investigators.
- To prepare annual continuing review reports at intervals designated by the WRAIR Human Use Review Committee and a final report in accordance with Title 21, Code of Federal Regulations, Part 312.33.
- To immediately report to WRAIR HURC knowledge of a pending compliance inspection by other outside governmental agency concerning clinical investigation or research.

With my signature I, as a Protocol Co-Chair or Principal Investigator, acknowledge that I have read the responsibilities and will comply with them. I understand that if I fail to comply with any of these responsibilities, all projects for which I am an investigator may be suspended.

____

Frederick Sawe Date

____

Douglas Shaffer Date

XXII. REFERENCES

1. United States Leadership against HIV/AIDS, Tuberculosis, and Malaria Act of 2003. Public Law 108-25. 108th Congress, 1st Sess. (2003).
2. Office of the US Global AIDS Coordinator. The President's Emergency Plan for AIDS Relief: US Five Year Global HIV/AIDS Strategy. Washington, D.C.; 2004.
3. Kenya ARV Stock Fact Sheet. President's Emergency Plan for AIDS Relief Coordinating Office. US Embassy. Nairobi. March 3, 2008.
4. Kenya Ministry of Health. Available at: http://www.health.go.ke/. Accessed March 10, 2008.
5. The Global Fund to Fight Aids, Tuberculosis and Malaria. Available at: http://www.theglobalfund.org/en/. Accessed March 10, 2008.
6. Clinton HIV/AIDS Initiative. William J. Clinton Foundation. Available at: http://www.clintonfoundation.org/cf-pgm-hs-ai-home.htm. Accessed March 10, 2008.
7. Médecins Sans Frontières (MSF). Available at : www.msf.org. Accessed March 10, 2008.
8. National AIDS and STD Control Programme. Guidelines for Antiretroviral Drug Therapy in Kenya. Ministry of Health, Kenya. 2005.
9. National AIDS and STD Control Programme. Kenya National Clinical Manual for ART Providers. A Concise and Practical Guide to ART Provision. 2nd Edition. Ministry of Health, Kenya. 2007.
10. Kimaiyo S. Program Manager, Academic Model for the Prevention and Treatment of HIV/AIDS. Personal communication. February 2008.
11. Smith D, Schooley R. Running With Scissors: Using Antiretroviral Therapy Without Monitoring Viral Load. Clin Infect Dis. 2008 May 15;46(10):1598-600.
12. Panel on Antiretroviral Guidelines for Adult and Adolescents. Guidelines for the use of antiretroviral agents in HIV-1-infected adults and adolescents. Department of Health and Human Services. January 29, 2008; 1-128. Available at http://www.aidsinfo.nih.gov/ContentFiles/AdultandAdolescentGL.pdf. Accessed March 27, 2008.

13. Shumbusho F. Viral Load Testing Before Advancing to 2nd line or Salvage ART Regimens: Observation In 75 Rwandan Patients Experiencing Clinical and/or Immunological failure. The President's Emergency Plan for AIDS Relief Implementers Meeting; Kigali, Rwanda. June 16-19, 2007.
14. Magembe G. Role of Combined, Repeat CD4 Count and Viral Load Tests in Defining First Line ARV Failure in Resource Poor Countries. The President's Emergency Plan for AIDS Relief Implementers Meeting; Kigali, Rwanda. June 16-19, 2007.
15. Gilks C. DART: *An Equivalence Trial of Treatment Monitoring Strategies in Africa*. Uganda and Zimbabwe. The President's Emergency Plan for AIDS Relief Implementers Meeting; Kigali, Rwanda. June 16-19, 2007.
16. Mermin J, Were W, Ekwaru J, et al. Mortality in HIV-infected Ugandan adults receiving antiretroviral treatment and survival of their HIV-uninfected children: a prospective cohort study. Lancet. 2008;371:752–59.
17. O-GAC Care and Treatment PHE Committee. Received July 1, 2008.
18. Ball T, Ji H, Kimani J, et al. Polymorphisms in IRF-1 associated with resistance to HIV-1 infection in highly exposed uninfected Kenyan sex workers. AIDS. 2007 May 31;21(9):1091-101.
19. Derache A, Traore O, Koita V, et al. Genetic diversity and drug resistance mutations in HIV type 1 from untreated patients in Bamako, Mali. Antivir Ther. 2007;12(1):123-9.
20. Church J, Hudelson S, Guay L, et al. HIV type 1 variants with nevirapine resistance mutations are rarely detected in antiretroviral drug-naive African women with subtypes A, C, and D. AIDS Res Hum Retroviruses. 2007;23(6):764-8.
21. Novitsky V, Wester C, DeGruttola V, et al. The reverse transcriptase 67N 70R 215Y genotype is the predominant TAM pathway associated with virologic failure among HIV type 1C-infected adults treated with ZDV/ddI-containing HAART in southern Africa. AIDS Res Hum Retroviruses. 2007;23(7):868-78.
22. Seyler C, Adjé-Touré C, Messou E, et al. Impact of genotypic drug resistance mutations on clinical and immunological outcomes in HIV-infected adults on HAART in West Africa. AIDS. 2007;21(9):1157-64.
23. Little S, Holte S, Routy J, et al. Antiretroviral drug resistance among patients recently infected with HIV. N Engl J Med. 2002;347(6):385-94.

24. Weinstock HS, Zaidi I, Heneine W, et al. The epidemiology of antiretroviral drug resistance among drug-naive HIV-1-infected persons in 10 US cities. J Infect Dis. 2004;189(12):2174-80.
25. Wensing AM, van de Vijver DA, Angarano G, et al. Prevalence of drug-resistant HIV-1 variants in untreated individuals in Europe: implications for Clinical management. J Infect Dis. 2005;192(6):958-66.
26. Cane P, Chrystie I, Dunn D, et al. Time trends in primary resistance to HIV drugs in the United Kingdom : multicenter observational study. BMJ. 2005;331(7529):1368.
27. Bennett D, McCormick L, Kline R, et al. US surveillance of HIV drug resistance at diagnosis using HIV diagnostic sera. 12th Conference on Retroviruses and Opportunistic Infections; Boston, MA, February 22-25, 2005..
28. Moore R, Keruly J, Gebo K, et al. An improvement in virologic response to highly active antiretroviral therapy in clinical practice from 1996 through 2002. J Acquir Immune Defic Syndr. 2005;39(2):195-8.
29. Committee for the Evaluation of the President's Emergency Plan for AIDS Relief (PEPFAR) Implementation. PEPFAR Implementation: Progress and Promise. National Academy of Sciences. 2007.
30. Center for International Health Development. Boston University School of Public Health. Available at: http://sph.bu.edu/index.php?option=com_content&task=view&id=289&Itemid=377. Accessed March 10, 2008.
31. Fox M, Rosen S, MacLeod W, et al. The impact of HIV/AIDS on labour productivity in Kenya. Trop Med and Int Health. 2004;9:318-24.
32. Larson B, Fox M, Rosen S, et al. Early effects of antiretroviral therapy on work performance: preliminary results from a cohort study of Kenyan agricultural workers. AIDS. 2008;22 (3):421-5
33. Rosen S, Long L, Sanne I. The outcomes and costs of different models of antiretroviral treatment delivery in South Africa. Boston University Center for International Health and Development, Boston MA. 2007.
34. The Impact of Highly Active Antiretroviral Therapy on Labor Productivity in the Rift Valley Province, Kenya. RV199. Boston University/Kenya Medical Research Institute/United State Army Medical Research Unit-Kenya (Simon / Wasunna / Shaffer). July 2005.

35. Economic Outcomes of Antiretroviral Therapy in the Southern Rift Valley Province, Kenya. RV216. Boston University/Kenya Medical Research Institute/United State Army Medical Research Unit-Kenya (Larson / Wasunna / Shaffer). May 2006.
36. Costs and Outcomes of Models for Delivering Antiretroviral Therapy for HIV/AIDS in Kenya. RV232. Boston University/Kenya Medical Research Institute/United State Army Medical Research Unit-Kenya (Larson / Wasunna / Shaffer). April 2007.
37. UNAIDS. Joint United Nations Programme on HIV/AIDS. 2007 AIDS Epidemic Update. Available at: http://www.unaids.org/en/KnowledgeCentre/HIVData/EpiUpdate/EpiUpdArchive/2007/. Accessed March 10, 2008.
38. U.S. Military HIV Research Program. Available at: http://hivresearch.org/. Accessed March 10, 2008.
39. Centers for Disease Control and Prevention. Department of Health and Human Services. Available at: http://www.cdc.gov/. Accessed March 10, 2008.
40. U.S. Agency for International Development. Available at: http://www.usaid.gov/. Accessed March 10, 2008.
41. National AIDS and STI Control Programme, Ministry of Health, Kenya. AIDS in Kenya. 7th ed. Nairobi, Kenya: NASCOP; 2005.
42. Foglia G, Sateren W, Renzullo P, et al. High prevalence of HIV infection among rural tea plantation residents in Kericho, Kenya. Epidemiology and Infection. 2008;136:694-702.
43. Ayisi J, van Eijk A, ter Kuile F, et al. Risk factors for HIV infection among asymptomatic pregnant women attending an antenatal clinic in western Kenya. Int J STD AIDS. 2000;11(6):393-401.
44. Kenya Medical Research Institute. Available at: http://www.kemri.org/home.html. Accessed March 10, 2008.
45. Walter Reed Army Institute of Research. Available at: http://wrair-www.army.mil/. Accessed March 10, 2008.
46. United States Army Medical Research Unit-Kenya. Available at: http://www.usamrukenya.org/. Accessed March 10, 2008.

47. HIV-1 Prevalence, Incidence, Cohort Retention, and Host Genetics and Viral Diversity in High Risk Cohorts in East Africa. RV217b, WRAIR #1373, KEMRI TBD.
48. A Phase I/II Clinical Trial to Evaluate the Safety and Immunogenicity of a Multiclade HIV-1 DNA Plasmid Vaccine Boosted by a Multiclade HIV-1 Recombinant Adenovirus-5 Vector Vaccine in HIV Uninfected Adult Volunteers in East Africa. http://clinicaltrials.gov/ct/show/NCT00123968?order=1. Accessed November 14, 2007.
49. Optimal Combination Therapy After Nevirapine Exposure (OCTANE). http://clinicaltrials.gov/ct/show/NCT00089505?order=1. Accessed November 14, 2007.
50. Antiretroviral therapy for HIV infection in adults and adolescents: recommendations for a public health approach. – 2010 rev. World Health Organization. Available at: http://www.who.int/hiv/pub/arv/adult2010/en/index.html. Accessed 13 June 13, 2011.
51. Guidelines for antiretroviral therapy in Kenya. 4th Edition 2011. Ministry of Medical Services, Republic of Kenya.
52. HIV Drug Resistance Early Warning Indicators. World Health Organization Indicators to Monitor HIV Drug Resistance Prevention at Antiretroviral Treatment Sites. June 2010 Update. Available at: http://www.who.int/hiv/topics/drugresistance/hiv_dr_early_warning_indicators.pdf. Accessed 13 June 2011.
53. Saag M, Westfall A, Luhanga D, et al. A Cluster Randomized Trial of Routine vs. Discretionary Viral Load Monitoring Among Adults Starting ART: Zambia. Abstract 6C, Session 24. 19th Conference on Retroviruses and Opportunistic Infections. March 5-8, 2012.
54. 1999 Kenya Census of Population and Housing: Tabulations General. Available at www.statssa.gov.za/events/conference/downloadfiles/presentations/session%203/Mike%20Levin.pdf. Accessed February 22, 2008.
55. Obiero E, Langat B, …, Shaffer D, et al. Scaling Up An HIV/AIDS Clinic In Rural Kenya: Experience From The Kericho District Hospital. Oral Abstract

Session. The President's Emergency Plan for AIDS Relief Second Annual Field Meeting; Addis Ababa, Ethiopia. May 22-27, 2005.

56. Antiretroviral Therapy for HIV Infections in Adults and Adolescents in Resource-Limited Settings: Towards Universal Access. World Health Organization. 2006 Revision. Available at: http://www.who.int/hiv/pub/guidelines/artadultguidelines.pdf. Accessed March 10, 2008.
57. Pharmacy and Poisons Board, Ministry of Health. Kenya. Available at: http://www.pharmacyboardkenya.org/. Accessed March 10, 2008.
58. U.S. Food and Drug Administration. Department of Health and Human Services. Available at: http://www.fda.gov. Accessed March 10, 2008.
59. Guidance for Industry Fixed Dose Combinations, Co-Packaged Drug Products, and Single-EntityVersions of Previously Approved Antiretrovirals for the Treatment of HIV. U.S. Department of Health and Human Services. Food and Drug Administration Center for Drug Evaluation and Research (CDER). Procedural

    October 2006. Available at: http://www.fda.gov/cder/guidance/6360fnl.pdf. Accessed March 10, 2008.
60. U.S. Food and Drug Administration. President's Emergency Plan for AIDS Relief. Approved and Tentatively Approved Antiretrovirals in Association with the President's Emergency Plan. Available at: http://www.fda.gov/oia/pepfar.htm. Accessed March 10, 2008.
61. ARV Usage Data. Management Sciences for Health. Nairobi, Kenya. March 19, 2008.
62. National AIDS and STD Control Programme. National Guidelines for Voluntary Counseling and Testing. Ministry of Health, Kenya. 2001.
63. National AIDS and STD Control Programme. Guidelines for HIV Testing in Clinical Settings. Ministry of Health, Kenya. 2004.
64. WHO case definitions of HIV for Surveillance and revised clinical staging and immunological classification of HIV related diseases in adults and children. World Health Organization. 2007. Available at: http://www.who.int/hiv/pub/guidelines/HIVstaging150307.pdf. Accessed March 10, 2008.

65. Drummond M, O'Brien B, Stoddart G, et al. Methods for the Economic Evaluation of Health Care Programmes. Second ed. Oxford: Oxford University Press; 1997.
66. Drummond M, McGuire A. Economic Evaluation in Health Care: Merging Theory with Practice. Oxford: Oxford University Press; 2001.
67. Shafer R, Rhee S, Pillay D, et al. HIV-1 protease and reverse transcriptase mutations for drug resistance surveillance. AIDS. 2007;21:215-233.
68. Johnson V, Brun-Vezinet F, Clotet B, et al. Update of the drug resistance mutations in HIV-1: 2007. Top HIV Med. 2007;15(4):119-25.
