# Supplementary material for: Kericho CLinic-Based ART Diagnostic Evaluation (CLADE): Design, Accrual, and Baseline Characteristics of a Randomized Controlled Trial Conducted in Predominately Rural, District-Level, HIV Clinics of Kenya
Source: PLoS One. 2015 Feb 23;10(2):e0116299. doi: 10.1371/journal.pone.0116299 (PMC4338154; doi:10.1371/journal.pone.0116299)
Supplement: S1 Appendices — (PDF) [file pone.0116299.s007.pdf]

Appendix I. CLADE: Clinic-based ART & Diagnostic Evaluation

A Public Health Evaluation of Routine vs. Viral Load Guided ART in ART Clinics in Rural Kenya

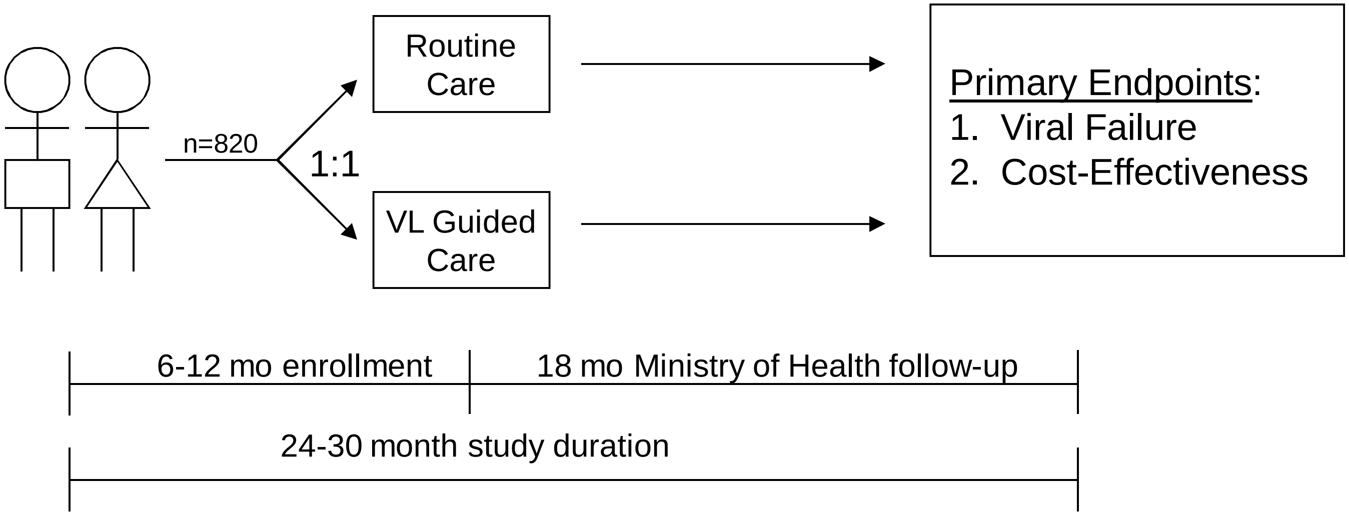

Secondary Endpoints:

1. Combined clinical outcome (AIDS progression as defined by 2 new WHO III or 1 new WHO IV event)
2. Death
3. Hospitalization
4. OIs
5. Adherence
6. Lost-to-follow-up
7. Viral resistance in treatment failures and all viral failures at the final study visit
8. Feasibility of VL monitoring
9. Costs of VL monitoring
10. GCP adherence
11. WHO HIVDR Early Warning Indicators
12. Long-term, 5-year follow-up of clinical, immunological, and virological (including HIVDR testing) parameters in a subset of participants.

Eligibility:

- Male or Female $\geq$ 18 y/o
- Identified by clinic staff as intending to begin ART

Baseline:

- Clinical exam
- Routine Labs
- WHO staging
- CD4, Arm-B Viral Load (VL)
- Resistance testing (population based, random n=200)

Routine Care:

- F/u q6mo with CD4s+WHO Staging and Targeted VL

Viral Load Guided Care:

- F/u q6mo with VL, CD4, WHO Staging or prn

Primary Analyses:

1. Chi Square/Fishers Exact tests and hazards analyses for VL endpoint
2. Cost-effectiveness based upon viral success and variable costs
3. Routine descriptive statistics based upon metrics of feasibility, GCP, and comparative secondary endpoints

CLADE V2.5_Nov 30 2012
O-GAC # KE 07-0044 RV257/WRAIR 1591 HRPO# A-14328 KEMRI # 1717

APPENDIX II. Study Sites and Allocated Enrollment

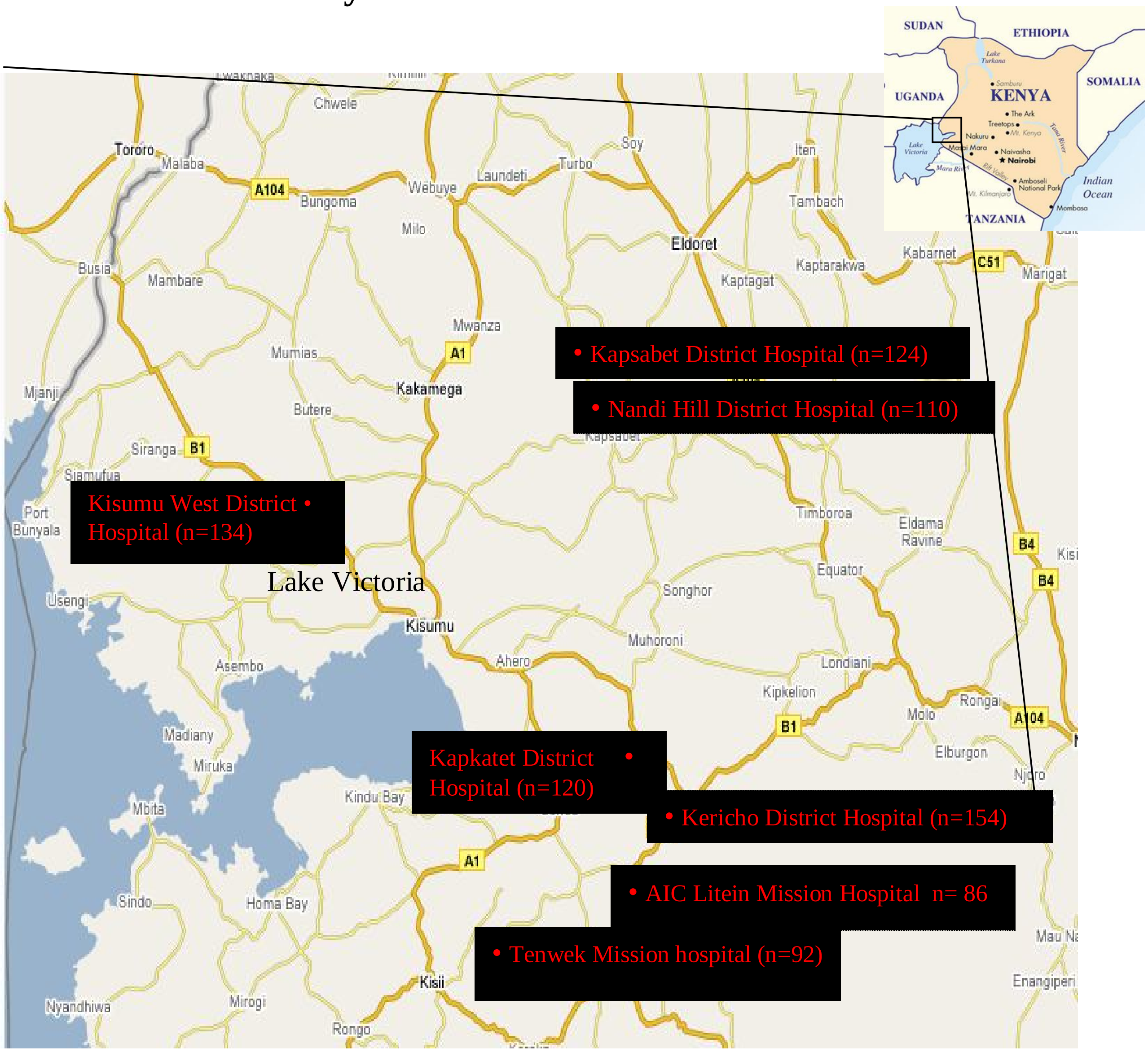

APPENDIX III. Pre-Study HIV Clinic Patient Flow and Study Enrollment

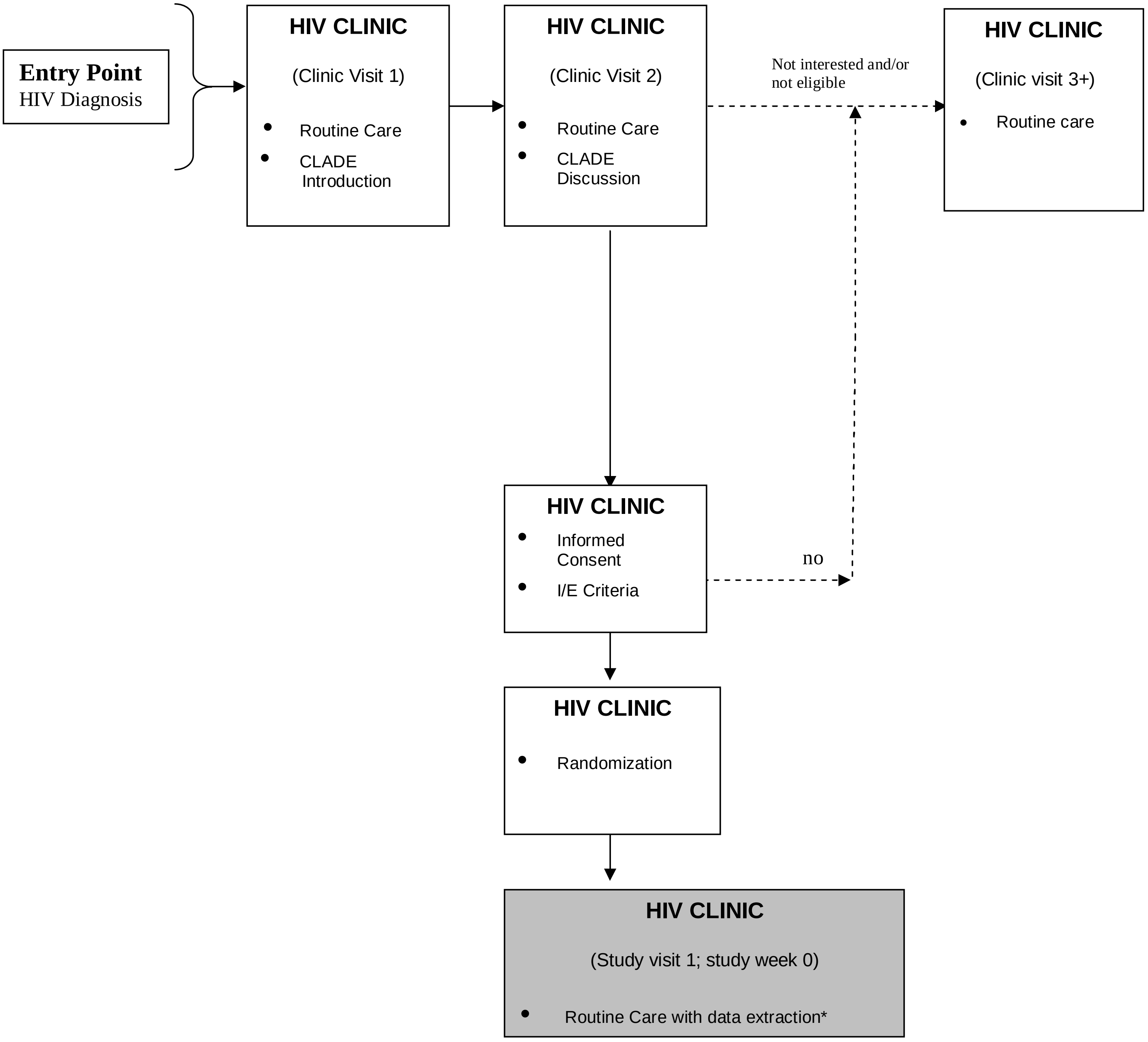

* Please see Appendix III and refer to protocol table 10, *"Routine Ministry of Health Data to be collected on Case Report Forms and in the Study Database"*

APPENDIX IV. Routine HIV/ART Clinic Flow and Study Encounters

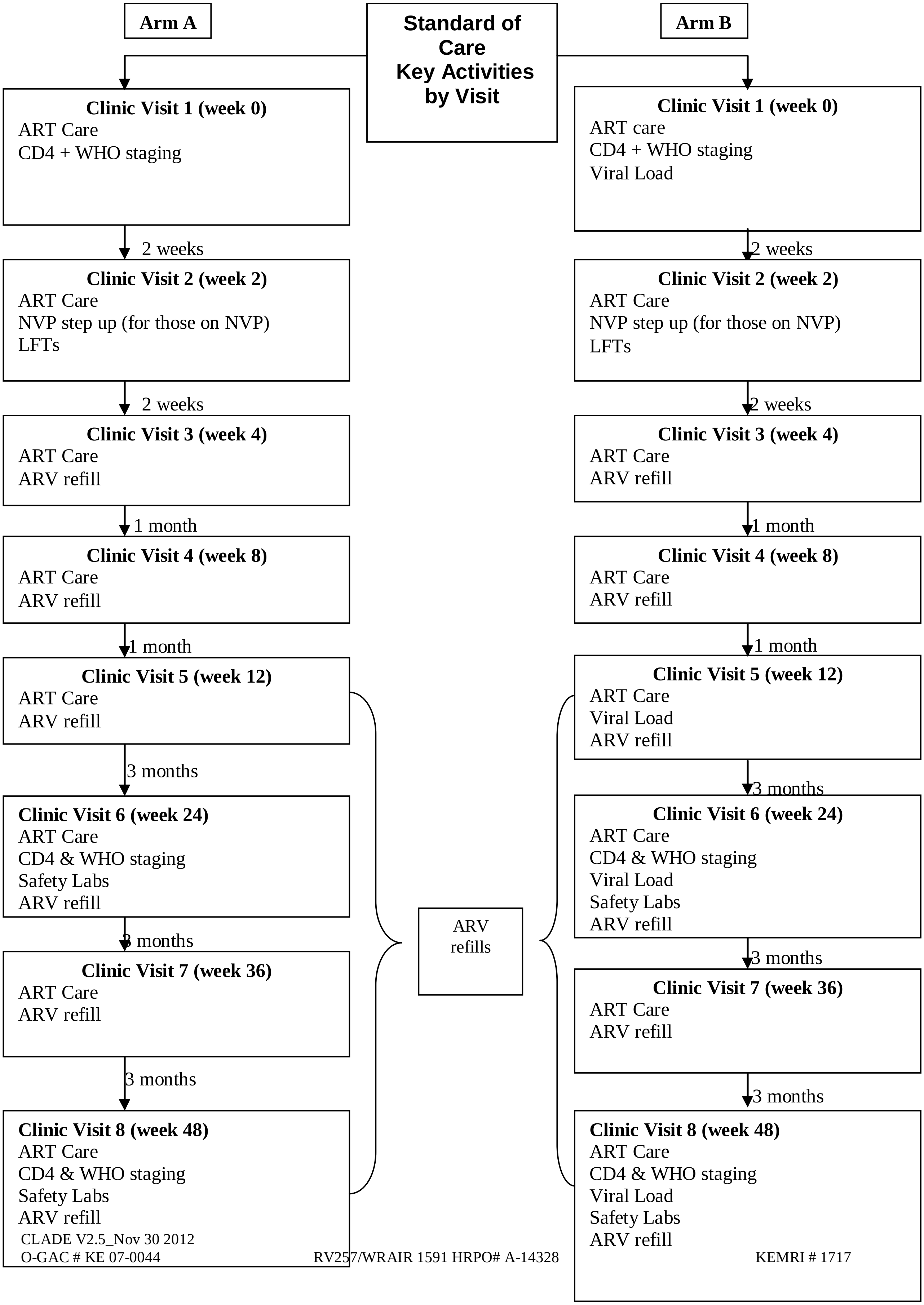

APPENDIX IV. Routine HIV/ART Clinic Flow and Study Encounters continued

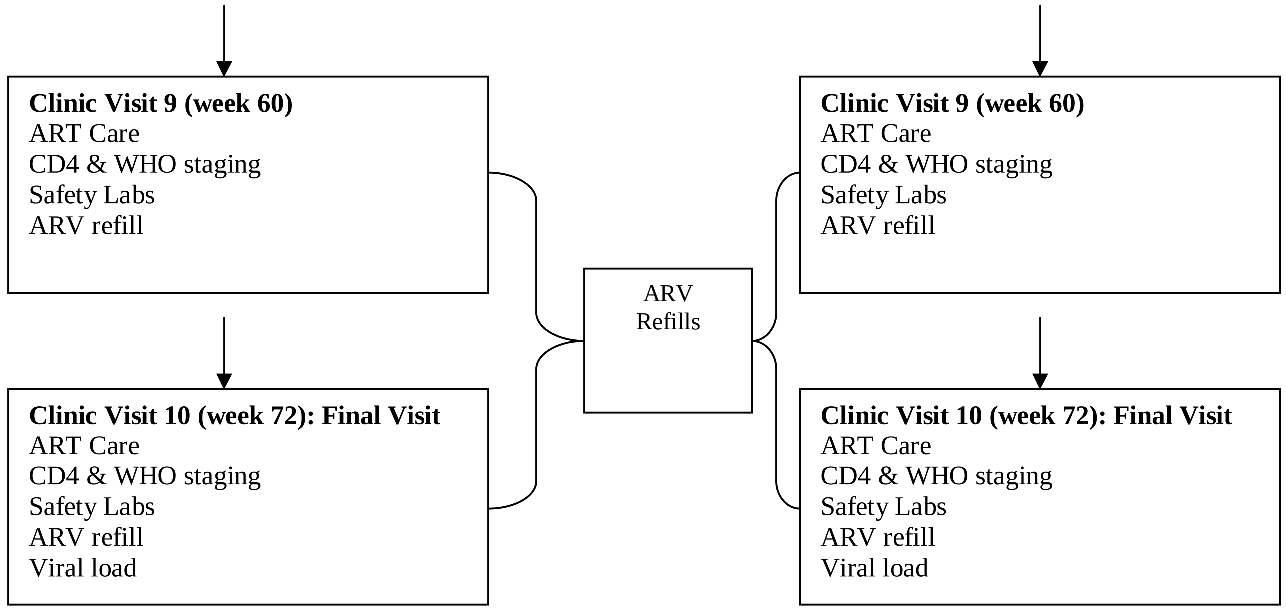

APPENDIX V. CLADE Viral Load/Treatment Failure Algorithm*

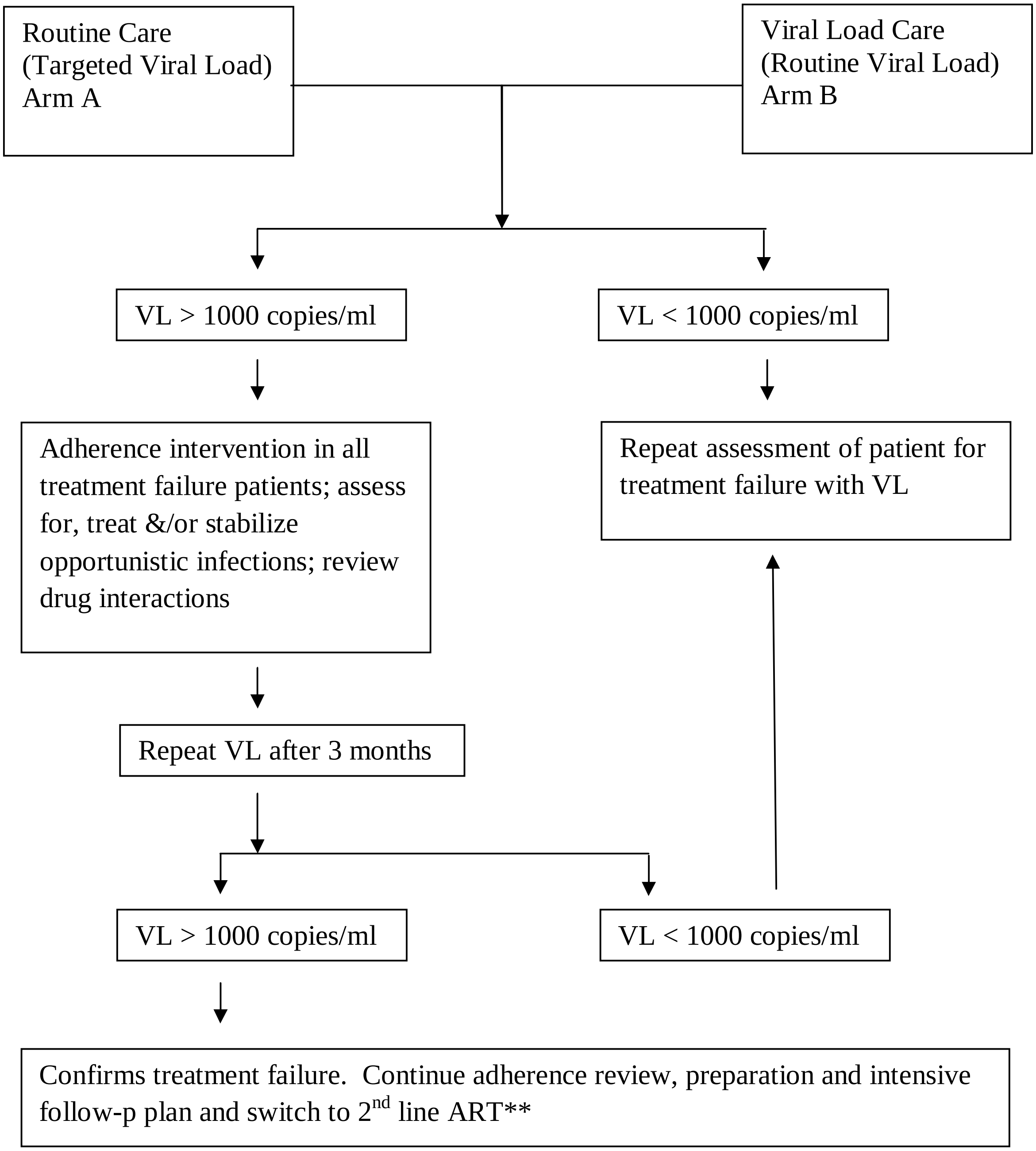

* Based upon Guidelines for antiretroviral therapy in Kenya. 4th Ed. Ministry of Medical Services, Republic of Kenya.

** All participants with treatment failure will have HIVDR testing done as part of the CLADE study.

APPENDIX VI. PEPFAR PHE Technical Team Resistance Testing Sample Size Calculations

Recommendations for number of specimens to test for HIV drug resistance

The WHO recommendations of N=96 to be tested for HIV drug resistance arise from a desire to have error bounds no larger than ± 10% when estimating a proportion. The widest bounds will occur when the proportion being estimated is 50% (i.e. the confidence intervals will be 40% - 60%). However we expect to find very few HIV-DR specimens and we should based the sample sizes considering very few specimens testing positive.

While there is no ‘right’ answer for the number to be tested, the table below may provide some insights. It shows the low and upper bounds on the estimated proportion HIV-DR based on the number of specimens that test drug resistant. It is followed by a graph that shows the upper bounds. It seems to me (at least) reasonable to based the number to be tested on the upper bound on the proportion resistant assuming that no specimens actually test resistant. The reasonable options for the number to be tested are N=100 (close to the 96 recommended by WHO), N=200, and N=300. The respective upper one-tailed bounds are 2.95%, 1.49%, and 0.99% (i.e. roughly 3%, 1.5% and 1%). N=200 seems to offer a reasonable balance between increasing sample size and upper bound estimate and would be my own recommendation for the number to test.

Exact Binomial Confidence Bounds
For the proportion HIV Drug Resistant

Two-sized alpha=0.05 bounds (one-sided bounds when # resistant = 0)

			Estimated Proportion not Resistant	Lower Bound	Upper Bound
			Sum	Sum	Sum
**N (Specimens Tests)**	**# nR (not Resistant)**	**# R (Resistant)**			
**50**	**46**	**4**	0.080000	0.022228	0.192343
	**47**	**3**	0.060000	0.012549	0.165482
	**48**	**2**	0.040000	0.004881	0.137138
	**49**	**1**	0.020000	0.000506	0.106470
	**50**	**0**	0.000000	0.000000	0.058155

			Estimated Proportion not Resistant	Lower Bound	Upper Bound
			Sum	Sum	Sum
**60**	**56**	**4**	0.066667	0.018462	0.161987
	**57**	**3**	0.050000	0.010432	0.139243
	**58**	**2**	0.033333	0.004063	0.115281
	**59**	**1**	0.016667	0.000422	0.089399
	**60**	**0**	0.000000	0.000000	0.048703
**70**	**66**	**4**	0.057143	0.015787	0.139894
	**67**	**3**	0.042857	0.008927	0.120179
	**68**	**2**	0.028571	0.003479	0.099429
	**69**	**1**	0.014286	0.000362	0.077044
	**70**	**0**	0.000000	0.000000	0.041893
**80**	**76**	**4**	0.050000	0.013789	0.123099
	**77**	**3**	0.037500	0.007801	0.105702
	**78**	**2**	0.025000	0.003042	0.087407
	**79**	**1**	0.012500	0.000316	0.067688
	**80**	**0**	0.000000	0.000000	0.036754
**90**	**86**	**4**	0.044444	0.012241	0.109901
	**87**	**3**	0.033333	0.006928	0.094336
	**88**	**2**	0.022222	0.002703	0.077978
	**89**	**1**	0.011111	0.000281	0.060357
	**90**	**0**	0.000000	0.000000	0.032738
**100**	**96**	**4**	0.040000	0.011004	0.099257
	**97**	**3**	0.030000	0.006230	0.085176
	**98**	**2**	0.020000	0.002431	0.070384

			Estimated Proportion not Resistant	Lower Bound	Upper Bound
			Sum	Sum	Sum
	99	1	0.010000	0.000253	0.054459
	100	0	0.000000	0.000000	0.029513
110	106	4	0.036364	0.009995	0.090492
	107	3	0.027273	0.005660	0.077637
	108	2	0.018182	0.002210	0.064138
	109	1	0.009091	0.000230	0.049611
	110	0	0.000000	0.000000	0.026866
120	116	4	0.033333	0.009156	0.083149
	117	3	0.025000	0.005186	0.071323
	118	2	0.016667	0.002025	0.058909
	119	1	0.008333	0.000211	0.045556
	120	0	0.000000	0.000000	0.024655
130	126	4	0.030769	0.008446	0.076907
	127	3	0.023077	0.004785	0.065959
	128	2	0.015385	0.001869	0.054469
	129	1	0.007692	0.000195	0.042113
	130	0	0.000000	0.000000	0.022781
140	136	4	0.028571	0.007838	0.071537
	137	3	0.021429	0.004441	0.061345
	138	2	0.014286	0.001735	0.050651
	139	1	0.007143	0.000181	0.039154
	140	0	0.000000	0.000000	0.021171
150	146	4	0.026667	0.007313	0.066868

			Estimated Proportion not Resistant	Lower Bound	Upper Bound
			Sum	Sum	Sum
	147	3	0.020000	0.004144	0.057334
	148	2	0.013333	0.001619	0.047333
	149	1	0.006667	0.000169	0.036583
	150	0	0.000000	0.000000	0.019773
160	156	4	0.025000	0.006853	0.062771
	157	3	0.018750	0.003884	0.053816
	158	2	0.012500	0.001517	0.044423
	159	1	0.006250	0.000158	0.034329
	160	0	0.000000	0.000000	0.018549
170	166	4	0.023529	0.006447	0.059146
	167	3	0.017647	0.003654	0.050704
	168	2	0.011765	0.001428	0.041850
	169	1	0.005882	0.000149	0.032337
	170	0	0.000000	0.000000	0.017468
180	176	4	0.022222	0.006087	0.055918
	177	3	0.016667	0.003450	0.047932
	178	2	0.011111	0.001348	0.039559
	179	1	0.005556	0.000141	0.030563
	180	0	0.000000	0.000000	0.016505
190	186	4	0.021053	0.005765	0.053023
	187	3	0.015789	0.003268	0.045448
	188	2	0.010526	0.001277	0.037505
	189	1	0.005263	0.000133	0.028974

			Estimated Proportion not Resistant	Lower Bound	Upper Bound
			Sum	Sum	Sum
	190	0	0.000000	0.000000	0.015643
200	196	4	0.020000	0.005476	0.050414
	197	3	0.015000	0.003104	0.043208
	198	2	0.010000	0.001213	0.035655
	199	1	0.005000	0.000127	0.027542
	200	0	0.000000	0.000000	0.014867
210	206	4	0.019048	0.005214	0.048049
	207	3	0.014286	0.002956	0.041179
	208	2	0.009524	0.001155	0.033978
	209	1	0.004762	0.000121	0.026245
	210	0	0.000000	0.000000	0.014164
220	216	4	0.018182	0.004976	0.045896
	217	3	0.013636	0.002821	0.039332
	218	2	0.009091	0.001103	0.032452
	219	1	0.004545	0.000115	0.025064
	220	0	0.000000	0.000000	0.013525
230	226	4	0.017391	0.004758	0.043928
	227	3	0.013043	0.002698	0.037643
	228	2	0.008696	0.001055	0.031057
	229	1	0.004348	0.000110	0.023985
	230	0	0.000000	0.000000	0.012940
240	236	4	0.016667	0.004559	0.042121
	237	3	0.012500	0.002585	0.036094

			Estimated Proportion not Resistant	Lower Bound	Upper Bound
			Sum	Sum	Sum
	238	2	0.008333	0.001011	0.029777
	239	1	0.004167	0.000105	0.022995
	240	0	0.000000	0.000000	0.012405
250	246	4	0.016000	0.004376	0.040457
	247	3	0.012000	0.002482	0.034667
	248	2	0.008000	0.000970	0.028598
	249	1	0.004000	0.000101	0.022084
	250	0	0.000000	0.000000	0.011911
260	256	4	0.015385	0.004207	0.038920
	257	3	0.011538	0.002386	0.033348
	258	2	0.007692	0.000933	0.027509
	259	1	0.003846	0.000097	0.021242
	260	0	0.000000	0.000000	0.011456
270	266	4	0.014815	0.004051	0.037495
	267	3	0.011111	0.002297	0.032126
	268	2	0.007407	0.000898	0.026500
	269	1	0.003704	0.000094	0.020462
	270	0	0.000000	0.000000	0.011034
280	276	4	0.014286	0.003906	0.036171
	277	3	0.010714	0.002215	0.030991
	278	2	0.007143	0.000866	0.025563
	279	1	0.003571	0.000090	0.019737
	280	0	0.000000	0.000000	0.010642

			Estimated Proportion not Resistant	Lower Bound	Upper Bound
			Sum	Sum	Sum
290	286	4	0.013793	0.003771	0.034937
	287	3	0.010345	0.002138	0.029933
	288	2	0.006897	0.000836	0.024689
	289	1	0.003448	0.000087	0.019062
	290	0	0.000000	0.000000	0.010277
300	296	4	0.013333	0.003645	0.033785
	297	3	0.010000	0.002067	0.028945
	298	2	0.006667	0.000808	0.023873
	299	1	0.003333	0.000084	0.018431
	300	0	0.000000	0.000000	0.009936
310	306	4	0.012903	0.003527	0.032706
	307	3	0.009677	0.002000	0.028020
	308	2	0.006452	0.000782	0.023110
	309	1	0.003226	0.000082	0.017841
	310	0	0.000000	0.000000	0.009617
320	316	4	0.012500	0.003416	0.031694
	317	3	0.009375	0.001938	0.027152
	318	2	0.006250	0.000758	0.022394
	319	1	0.003125	0.000079	0.017288
	320	0	0.000000	0.000000	0.009318
330	326	4	0.012121	0.003312	0.030743
	327	3	0.009091	0.001879	0.026336
	328	2	0.006061	0.000735	0.021720

			Estimated Proportion not Resistant	Lower Bound	Upper Bound
			Sum	Sum	Sum
	329	1	0.003030	0.000077	0.016767
	330	0	0.000000	0.000000	0.009037
340	336	4	0.011765	0.003215	0.029847
	337	3	0.008824	0.001823	0.025568
	338	2	0.005882	0.000713	0.021086
	339	1	0.002941	0.000074	0.016277
	340	0	0.000000	0.000000	0.008772
350	346	4	0.011429	0.003122	0.029002
	347	3	0.008571	0.001771	0.024844
	348	2	0.005714	0.000693	0.020488
	349	1	0.002857	0.000072	0.015815
	350	0	0.000000	0.000000	0.008523
360	356	4	0.011111	0.003035	0.028203
	357	3	0.008333	0.001722	0.024159
	358	2	0.005556	0.000674	0.019923
	359	1	0.002778	0.000070	0.015379
	360	0	0.000000	0.000000	0.008287
370	366	4	0.010811	0.002953	0.027447
	367	3	0.008108	0.001675	0.023511
	368	2	0.005405	0.000655	0.019389
	369	1	0.002703	0.000068	0.014966
	370	0	0.000000	0.000000	0.008064
380	376	4	0.010526	0.002875	0.026731

			Estimated Proportion not Resistant	Lower Bound	Upper Bound
			Sum	Sum	Sum
	377	3	0.007895	0.001631	0.022897
	378	2	0.005263	0.000638	0.018882
	379	1	0.002632	0.000067	0.014574
	380	0	0.000000	0.000000	0.007853
390	386	4	0.010256	0.002801	0.026051
	387	3	0.007692	0.001589	0.022315
	388	2	0.005128	0.000622	0.018401
	389	1	0.002564	0.000065	0.014203
	390	0	0.000000	0.000000	0.007652
400	396	4	0.010000	0.002731	0.025405
	397	3	0.007500	0.001549	0.021761
	398	2	0.005000	0.000606	0.017944
	399	1	0.002500	0.000063	0.013850
	400	0	0.000000	0.000000	0.007461

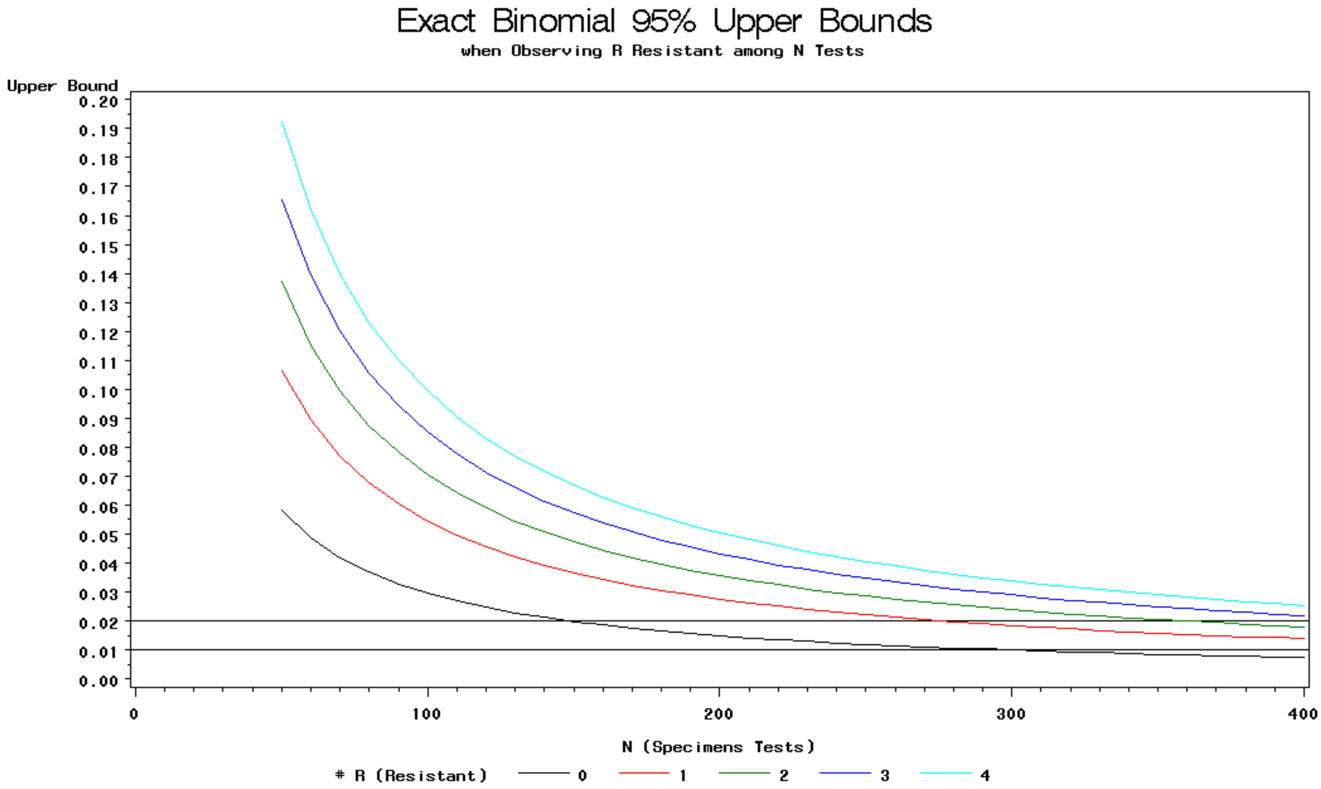
Exact Binomial 95% Upper Bounds
when Observing R Resistant among N Tests
Upper Bound
0.20
0.19
0.18
0.17
0.16
0.15
0.14
0.13
0.12
0.11
0.10
0.09
0.08
0.07
0.06
0.05
0.04
0.03
0.02
0.01
0.00
0
100
200
300
400
N (Specimens Tests)
R (Resistant)
0
1
2
3
4

APPENDIX VII. COSTING INFORMATION

PARAMETERS DATA

SITE PARAMETERS

Full name of site ______________________

Location ______________________

Province ______________________

Setting ______________________

Sector ______________________

Type ______________________

FINANCIAL PARAMETERS

Local currency ______________________

Comparison currency ______________________

Exchange rate ______________________ Date

______________________ Source

Entry currency ______________________

Currency year of results ______________________

Inflation rate ______________________ Date

______________________ Source

STUDY PARAMETERS

Period of study ______________________ Start Date

______________________ End Date

Number of subjects in study ______________________

ART

Number of active patients at site during study period ______________________

Average number of visits per annum ______________________

SUBJECT DATA

The following information needs to be available for each study subject. It should be stored in an electronic database with no identifiers.

BASELINE DATA (one record per subject)

Age (at ARV initiation) ______________________

Date of eligibility (First CD4 count < 200) ______________________

Date of ARV initiation ______________________

First line regimen ______________________

Height (cm) ______________________

Still at site at end of study period ______________________

If not, reason for not being in care ______________________

Date of death (if applicable) ______________________

Date of last visit (if no longer attending) ______________________

Date of transfer (if applicable) ______________________

VISIT DATA (multiple records per subject)

Visit date ______________________

Professionals seen ______________________

Weight (kg) ______________________

WHO III/IV Conditions ______________________

Lab tests done (with results for VL and CD4) ______________________

Drugs prescribed (Name, strength, dosage) ______________________

Drugs dispensed (How much of each drug) ______________________

Support services taken up ______________________

Next scheduled drug pickup ______________________

Next scheduled doctor/nurse visit ______________________

(To be extracted from the main CLADE data-CRF)

STAFF DATA

Title	Employment (MM/YY)		Cost to company	Programme Share
	Start	End	Currency	%

BUILDINGS DATA

Building	Ownership		Current Value	Rental		Square metres	
	Bought	Rented	Currency	Estimated	Actual	Total	Programme share (%)

Operations and Maintenance	
Item	Average cost per period

VEHICLES DATA

Vehicle	Ownership		Value			Rental		Programme Share
	Bought	Rented	Purchase Price	Year	Working Life	Estimated	Actual	%

Operations and Maintenance

Item	Average cost per period

EQUIPMENT DATA

Equipment	Ownership		Value			Rental		Programme Share
	Bought	Rented	Purchase Price	Year	Working Life	Estimated	Actual	%

Operations and Maintenance

Item	Average cost per period

SUPPLIES

Supplies	Description	Unit	Cost			Project Share	Project per month cost
			Unit Cost	Units Consumed	Cost per Month	%	KES
Supply 1							
Supply 2							
Supply 3							
Supply 4							
Supply 5							
Supply 6							
Supply 7							
Supply 8							
Supply 9							
Supply 10							
Supply 11							
Supply 12							
Supply 13							
Supply 14							
Supply 15							
Supply 16							
Supply 17							
Supply 18							
Supply 19							
Supply 20							
Supply 21							
Supply 22							
**Total**							
